# Pyracylenes: Facile Synthetic Access and Continuous Face-To-Face Antiaromatic π‑Stacking

**DOI:** 10.1021/jacsau.5c01515

**Published:** 2026-01-19

**Authors:** Sheng-Yuan Cheng, Jiun-Siang Juang, Yu-Fang Huang, Ting-Yi Lai, Yi-Hung Liu, Fumitaka Ishiwari, Akinori Saeki, Kenji Okada, Ryohei Kishi, Jeffrey M. Farrell

**Affiliations:** † Department of Chemistry, 33561 National Taiwan University, No. 1, Sec. 4, Roosevelt Rd., Taipei 10617, Taiwan; ‡ Department of Applied Chemistry, Graduate School of Engineering, 13013 Osaka University, Suita, Osaka 565-0871, Japan; § Graduate School of Urban Environmental Sciences, Tokyo Metropolitan University, 1-1 Minamiosawa, Hachioji, Tokyo 192-0297, Japan; ∥ Innovative Catalysis Science Division, Institute for Open and Transdisciplinary Research Initiatives (ICS-OTRI), The University of Osaka, 2-1 Yamadaoka, Suita, Osaka 565-0871, Japan; ⊥ Graduate School of Engineering Science, The University of Osaka, 1-3 Machikaneyama, Toyonaka, Osaka 560-8531, Japan; # Center for Quantum Information and Quantum Biology, The University of Osaka, 1-3 Machikaneyama, Toyonaka, Osaka 560-8531, Japan; ¶ Center for Emerging Materials and Advanced Devices, National Taiwan University, No. 1, Sec. 4, Roosevelt Rd., Taipei 10617, Taiwan

## Abstract

Pyracylene is a nonalternant hydrocarbon which has long fascinated chemists, but whose structure is exceedingly rare in experimental reports. Pyracylene’s surprising absence arises from synthetic inaccessibility attributable to its antiaromatic character. Meanwhile, sterically unimpeded polycyclic antiaromatic hydrocarbons have become fascinating candidates for organic materials applications and for antiaromatic π-stacked assemblies. Herein, we report a facile synthesis of pyracylenes via Pd-catalyzed ring contraction of diborinic acids. So-formed pyracylenes were derivatized by bromination and subsequent Pd-catalyzed cross-coupling. All pyracylenes were isolated, fully characterized, and studied by UV–vis absorption spectroscopy, cyclic voltammetry, density functional theory (DFT) calculations, and X-ray crystallography. In the solid state, three pyracylenes formed unprecedented continuous, face-to-face, antiaromatic π-stacks with equidistant molecular spacings and 90° twist angles between monomers, wherein HOMOs of monomers aligned with phase-complementary LUMOs of their neighbors (and vice versa). Nucleus-independent chemical shift (NICS) calculations indicated that this arrangement leads to antiaromaticity reduction of monomers. Solid-state conductivities of the pyracylenes were established using time-resolved microwave conductivity measurements.

## Graphical abstract

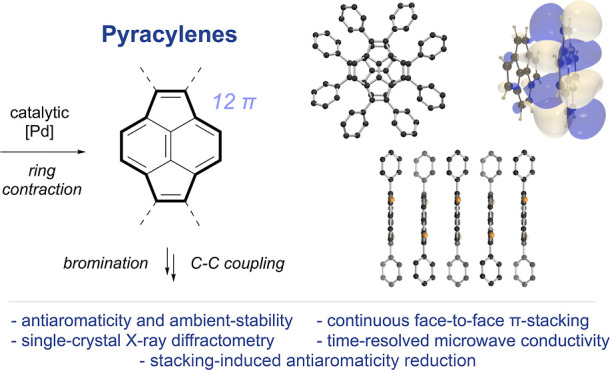

## Introduction

Polycyclic aromatic hydrocarbons (PAHs) are pervasive structures throughout chemistry and materials science. Polycyclic antiaromatic hydrocarbons (PAAHs) are comparatively less explored due to characteristic high reactivities that lead to problematic syntheses and handling, but have experienced growing interest. Unique properties of PAAHs, such as small HOMO–LUMO energy gaps, single-molecule conductivities,^1^ unique excited-state dynamics,^2^ and relationships to diradicals,^3^ could potentially produce transformative functional organic materials.^4^ Indeed, small HOMO–LUMO energy gaps and charge-carrier transport properties of some PAAHs (e.g., Figure 1a) have been leveraged for organic transistors.^5^

**Figure 1:**
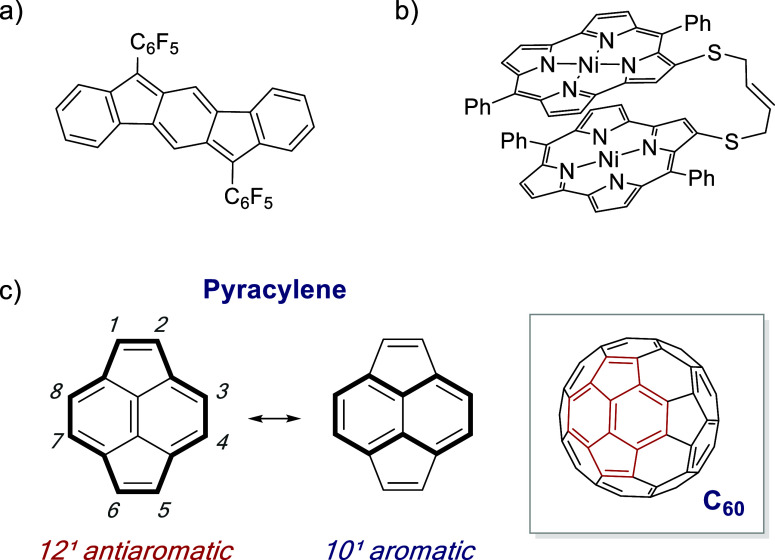
(a) A PAAH used in transistors by Haley, Nuckolls, and co-workers.^5b^ (b) Example of a face-to-face π-stacked norcorrole dimer reported by Shinokubo, Kim, Kowalczyk, and co-workers.^7a^ (c) The structure of pyracylene illustrating (anti)­aromatic circuits and its relationship to fullerene (inset).

The unique antiaromatic electronic structures of PAAH π-systems also suggest fundamentally different π–π interactions than those between PAH π-systems. For example, face-to-face π-stacking of antiaromatic π-systems may diminish or eliminate their antiaromaticities.^6^ Shinokubo and co-workers have recently reported breakthrough experimental evidence for this “stacked-ring aromaticity” in face-to-face π-stacked norcorrole oligomers (e.g., Figure 1b).^7^ However, to our knowledge there have been no reports of related antiaromaticity-stabilizing orbital interactions in continuous, well-defined, face-to-face π-stacks. Indeed, there have been relatively few experimental studies of π-stacked assemblies of PAAHs in general, which has limited understanding of their unique interactions and self-assembly behaviors. Contributing to experimental difficulties, PAAH stabilization via sterics is largely precluded for such studies, as this impedes π–π stacking.

Pyracylene (Figure 1c) is a planar nonalternant hydrocarbon (and fullerene substructure) which can be described as a 10π-electron [4*n* + 2] aromatic naphthalene system bearing two ethylene bridges, or as a 12π-electron [4*n*] antiaromatic system based on its conjugated periphery.^8^ Although pyracylene has intrigued chemists for decades,^9^ this deceptively simple molecule and its derivatives remain nearly absent from experimental accounts. After a failed synthetic attempt by Anderson and Anderson,^8a^ seminal work from Trost and co-workers reported the generation of pyracylenes in solution (Figure 2).^8b,10^ These species eluded isolation, but exhibited comparatively upfield-shifted periphery ^1^H chemical shifts attributable to the effects of paramagnetic ring currents.^8b,10,11^ Pyracylene was finally isolated via flash vacuum pyrolysis by Schaden in low yield,^12,13^ and studies of this molecule by the groups of Wirz^14^ and Minas Da Piedade^15^ indicated that the molecule is energetically stable and readily handled in pure form, despite antiaromatic character on magnetic and electronic grounds. This suggests (1) that pyracylenes bear a combination of stability and antiaromatic character, and (2) that the current dearth of pyracylenes is a problem of synthetic methodology rather than inherent instability. While fusion of aromatic rings at the pyracylene 1,2 and 5,6 positions allows synthetic access to species with fascinating properties,^16^ these are magnetically and electronically distinct from pyracylene, and often lack antiaromatic characteristics. To our knowledge, the lone example of a nonfused pyracylene isolated from a solution-phase synthesis is a 1,2:5,6-bis­(ethylenedithio)­pyracylene synthesized via tellurium tetrachloride mediated ring expansion (Figure 2).^17^

**Figure 2:**
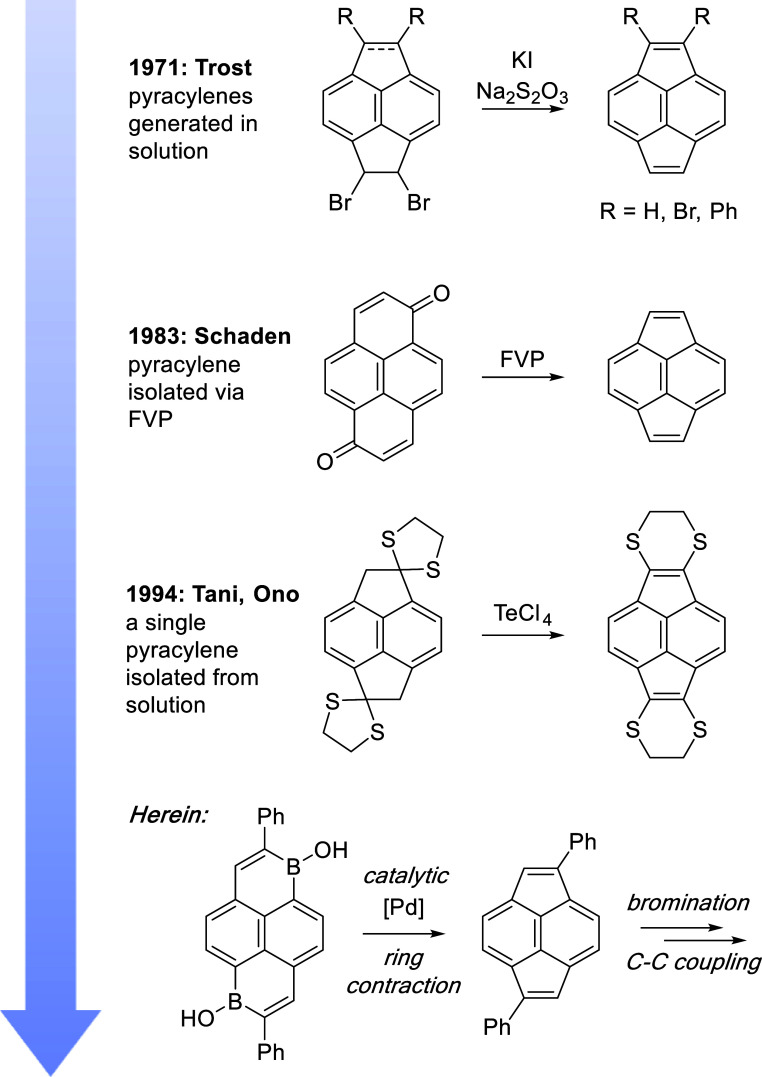
Timeline of progress in the synthesis of pyracylenes and the work presented herein.

Herein we describe a facile, catalytic, solution-phase synthesis of ambient-stable pyracylenes via Pd-catalyzed ring contraction of polycyclic borinic acids. These pyracylenes were readily brominated in high yields, and were subsequently amenable to Suzuki–Miyaura cross-coupling. All pyracylenes herein were studied by ^1^H and ^13^C NMR spectroscopy, high-resolution mass spectrometry (HRMS), UV–vis absorption spectroscopy, cyclic voltammetry, density functional theory (DFT) calculations, and X-ray crystallography. In some cases, the solid-state structures of pyracylenes revealed striking 1D π-stacks with equidistant molecular spacings, wherein adjacent molecules adopted unusual face-to-face positionings with 90° twist angles. DFT calculations indicated that this stacking arrangement aligned HOMOs and LUMOs of adjacent pyracylene monomers and diminished their antiaromaticities. Time-resolved microwave conductivity (TRMC) measurements of crystalline pyracylenes revealed conductivities in the solid state.

## Results and Discsussion

### Synthesis of Pyracylenes **1–3**

We envisioned Pd-catalyzed ring-contraction of previously reported 2,7-diphenyl-1,8-dihydroxy-1,8-diborapyrene (**I**) or 2,7-diphenyl-1,6-dihydroxy-1,6-diborapyrene (**II**) as potential routes to pyracylenes (Figure 2 and Scheme 1). Precursors **I** and **II** are conveniently prepared using a one-pot procedure of Würthner and co-workers.^18^ Moreover, Fu and co-workers have previously reported Pd-catalyzed ring contractions of dibenzoxaborininols^19^ that intimate possible pyracylene synthesis by ring contractions of **I** or **II**.

**Scheme 1:**
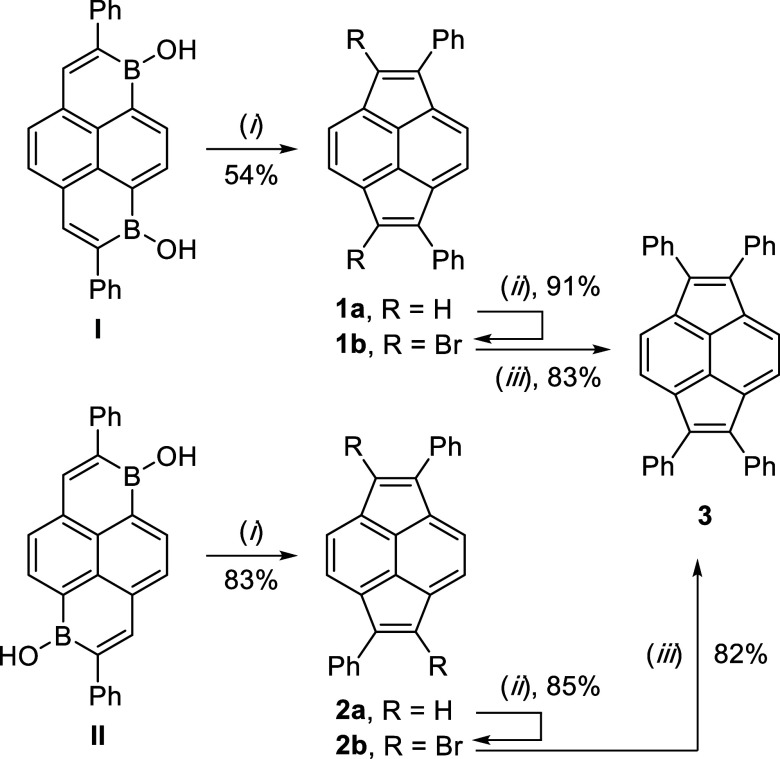
Optimized Catalytic Synthesis of Pyracylenes **1a** and **2a** via Ring Contraction, Synthesis of Brominated Pyracylenes **1b** and **2b**, and Suzuki Coupling Reactions of **1b** and **2b** to Form **3** ^a^

a (i) Pd­(OAc)_2_ (20 mol %), 1,4-benzoquinone (2 equiv), toluene or CH_3_CN, H_2_O, [*n*-Bu_4_N]­[Br], 70 °C, 2 h. (ii) *N*-bromosuccinimide (2 equiv), CHCl_3_, −35 °C—r.t., 3 h. (iii) PhB­(OH)_2_ (2.2 equiv), Pd­(PPh_3_)_4_ (10 mol %), Cs_2_CO_3_, toluene, 100 °C, 16 h.

To explore a ring contraction route to a pyracylene, we probed 1:2 stoichiometric reactions of **I** with Pd­(OAc)_2_ in CD_3_CN. Heating this reaction at 70 °C for 30 min gave rise to distinctive ^1^H NMR singlets consistent with paratropically shifted pyracylene periphery protons (δ = 6.47, 6.76, and 7.18) that we attributed to structure **1a**. An analogous reaction was carried out on a preparative scale in CH_3_CN at 70 °C, from which pyracylene **1a** could be isolated as a green solid in 11% yield after solvent removal in vacuo and purification by column chromatography. The structure of pyracylene **1a** was confirmed by ^1^H and ^13^C NMR spectroscopy as well as HRMS (see Supporting Information). Repeating our stoichiometric NMR-scale reactions of **I** with Pd­(PPh_3_)_4_, PdCl_2_(PPh_3_)_2_, Ni­(OAc)_2_, or Cu­(OAc)_2_ in place of Pd­(OAc)_2_ led to no observable pyracylene formation by ^1^H NMR spectroscopy.

We then sought to develop a catalytic variant of the ring contraction of **I**. While Fu and co-workers’ Pd-catalyzed ring contraction of dibenzoxaborininols involved oxidative cleavage of a B–C bond by I_2_ prior to Suzuki-type coupling,^19^ the C–C bond-forming step of Pd-mediated ring-contraction of **I** apparently requires no exogenous oxidant, indicating a divergent mechanism. Nevertheless, an oxidant would presumably be required to regenerate an active Pd species for a catalytic cycle. Thus, we began catalytic screening by reacting **I** with 20 mol % Pd­(OAc)_2_ in CH_3_CN at 70 °C for 2 h with 2 equiv of 1,4-benzoquinone as a terminal oxidant (see Supporting Information Table S1). Pyracylene **1a** could be isolated from this reaction in 14% yield, indicating probable catalytic turnover. Use of water as a cosolvent (1:10 water/CH_3_CN) led to a lower yield for this reaction, but when used in conjunction with 10 equiv of [*n*-Bu_4_N]­[Br], isolated yields of **1a** increased to 35%. Performing the reaction under N_2_ atmosphere further increased the yield to 54%, although degassing solvent offered no further improvement. Replacing CH_3_CN solvent with toluene offered similar yields of **1a** (56%, or 54% upon scale-up), whereas replacement with DMF, DMSO, or dioxane led to inferior yields. Using otherwise optimized conditions, replacement of 1,4-benzoquinone with Cu­(OAc)_2_, AgOAc, TEMPO, or MnO_2_ gave inferior yields. Our optimized catalytic conditions were then applied to the reaction of precursor **II**. Thus, **II** was reacted in the presence of Pd­(OAc)_2_ (20 mol %), 1,4-Benzoquinone (2 equiv), and [*n*-Bu_4_N]­[Br] (10 equiv) at 70 °C for 2 h in acetonitrile/water. In this case, product isolation was facilitated by the poor solubility of **2a** in this solvent mixture. Pure **2a** was isolated as a dark green solid in 83% yield after solvent washing steps, filtration of a CH_2_Cl_2_ solution through Celite, and solvent removal in vacuo.

To explore possible avenues of derivatization, **1a** and **2a** were each reacted with two equivalents of *N*-bromosuccinimide in CHCl_3_ for 3 h (−35 °C—r.t.) to afford dibrominated pyracylenes **1b** and **2b** in high yields (Scheme 1). In contrast, in situ reactions of pyracylenes with Br_2_ performed by Trost and co-workers led to addition products rather than bromide-substituted pyracylenes.^10b^ Brominated pyracylenes **1b** and **2b** were then probed for Pd-catalyzed C–C coupling reactivity as a possible derivatization route. To our delight, **1b** and **2b** were each effective coupling partners in Suzuki–Miyaura reactions with phenyl boronic acid, catalyzed by 10 mol % Pd­(PPh_3_)_4_ in toluene for 16 h at 100 °C with Cs_2_CO_3_ as a base (Scheme 1). In each reaction, a red precipitate formed that could be isolated by filtration, washing, and recrystallization from hot CHCl_3_ to yield pure 1,2,5,6-tetraphenylpyracylene **3** in 83% or 82% yield from **1b** or **2b**, respectively.

Pyracylenes **1–3** were fully characterized by ^1^H NMR spectroscopy, ^13^C NMR spectroscopy, HRMS, and single-crystal X-ray crystallography. For each, relatively upfield ^1^H chemical shifts were observed for protons on the pyracylene periphery compared to structurally related 1-phenylacenaphthylene, 1,2-dihydropyracylene, or 1,2-diphenyl-5,6-dihydropyracylene (see Supporting Information).^10b,20^ Echoing previous reports of the stability of pyracylene in pure form,^14^ solid samples of **1–3** stored in air for 7 days showed no observable decomposition by ^1^H NMR spectroscopy. Solution-phase stabilities were assessed in CDCl_3_ solutions kept under ambient, aerobic conditions. While **1a** shows onset of decomposition after 1 day by ^1^H NMR spectroscopy, **2a** shows onset of decomposition after 2 days. Remarkably, brominated pyracylenes **1b** and **2b** showed no signs of decomposition after 7 days in solution. The tendency of **3** to precipitate from solution over time prevented accurate assessment of its solution-phase stability. Sublimations of each compound **1**–**3** were possible under vacuum (10^–5^ Torr) at temperatures between 110 °C–160 °C (see Supporting Information for details). Interestingly, Jenneskens and co-workers have noted that unsubstituted pyracylene could not be resublimed after FVP synthesis.^13a^

### UV–Vis Spectroscopy and Voltammetry Studies of **1–3**

UV–vis measurements were performed on ∼10^–5^ M solutions of pyracylenes **1**–**3** in CH_2_Cl_2_ (Figure 3a,b and Table 1). Notably, each compound exhibited a broad absorption band, featuring low absorption intensity in the region of ∼500–800 nm. Such weak long-wavelength absorptions are typical for PAAHs, representing their low-energy, but symmetry-forbidden, HOMO–LUMO transitions.^21^ The UV–vis spectra of **1**–**3** simulated by TD-DFT calculations were in good agreement with the experimental results (Figure S55). The reason for the relatively large absorption intensity in the region of ∼500–800 nm in **1a** is due to a larger symmetry-lowering effect of the substituent introduction (see Supporting Information). Fluorescence spectroscopy indicated no measurable emissions from dilute (∼10^–5^ M) solutions of **1**–**3** in degassed CH_2_Cl_2_ under excitation at various λ_ex_ (each close to respective local absorption maxima of **1**–**3**, see Supporting Information). The nonemissive behavior of these compounds in solution is consistent with unsubstituted pyracylene,^14^ and is common for antiaromatic molecules in general.

**Figure 3:**
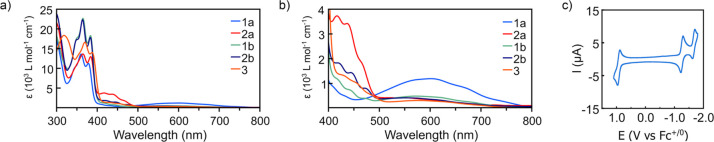
(a) UV–vis absorption spectra of pyracylenes **1–3** (∼10^–5^ M in CH_2_Cl_2_, 298 K). (b) Magnified 400–800 nm regions of UV–vis absorption spectra of pyracylenes **1–3** (∼10^–5^ M in CH_2_Cl_2_, 298 K). (c) Cyclic voltammogram of **1b** (1.6 × 10^–4^ M, 0.1 M [*n*-Bu_4_N]­[PF_6_], in CH_2_Cl_2_, vs Fc^+/0^, 298 K).

**Table 1:** Optical and Electronic Properties of **1**–**3** ^a^

	λ_abs_ [nm] (ε [M^–1^ cm^–1^])	*E*_1/2ox_ [V]	*E*_1/2 red_ 1 [V]	*E*_1/2 red_ 2 [V]	*E*_gap_ ^e^ [eV]
**1a**	600 (1200), 377 (11,100), 360 (13,600)	0.63^c^	–1.47 (−1.50)^b^	- (−1.90)^b^ ^,^ ^c^	2.06^f^
**2a**	514 (400), 436 (3400), 417 (3700), 384 (13,000), 365 (13,600)	0.66^c^	–1.47 (−1.48)^b^	- (−1.87)^b^ ^,^ ^c^	2.10^f^
**1b**	572 (500), 383 (18,300), 365 (22,700)	0.92^d^	–1.26	–1.67^d^	2.18
**2b**	561 (400), 470 (800), 444 (1400), 383 (17,800), 364 (22,300)	0.91^d^	–1.28	–1.67^d^	2.19
**3**	578 (300), 387 (14,000), 370 (16,600), 318 (18,400)	0.67^d^	–1.45 (−1.44)^b^	- (−1.80)^b^ ^,^ ^c^	2.12

a Optical measurements were carried out at 298 K in ∼10^–5^ M in CH_2_Cl_2_. Electrochemical measurements carried out at 298 K in 10^–4^ to 10^–3^ M solutions of 0.1 M [*n*-Bu_4_N]­[PF_6_] in CH_2_Cl_2_ or

b THF. Electrochemical potentials were calibrated with ferrocene as an internal standard and are referenced vs Fc^+/0^.

c Describes peak potential of an irreversible wave.

d Describes a quasi-reversible wave.

e *E*_gap_ is the HOMO–LUMO energy gap in eV estimated by difference (in V) between *E*_1/2ox_ and *E*_1/2 red_ 1 or

f between anodic peak potentials of first reductions and first oxidations. All other measurement details are available in the Supporting Information.

Redox properties of compounds **1**–**3** were studied by cyclic voltammetry in 10^–4^ to 10^–3^ M solutions in 0.1 M [*n*-Bu_4_N]­[PF_6_] CH_2_Cl_2_ (e.g., Figure 3c and Table 1). Facile, reversible one-electron reductions were measured for each compound. These occur at −1.45 V to −1.47 V vs Fc^+/0^ for **1a**, **2a**, and **3**, reflecting their electron-deficient fused five-membered ring-containing π-systems. These also fall in line with unsubstituted pyracylene, for which a first reduction at −1.056 V vs SCE in DMF was reported. For brominated **1b** and **2b**, first reversible one-electron reductions occur at −1.26 V and −1.28 V vs Fc^+/0^, approximately 0.2 V more positive than **1a**, **2a**, or **3**. For comparison the first one-electron reduction potential of popular fullerene derivative PC_61_BM is reported at −1.17 V vs Fc^+/0^ (in *o*-dichlorobenzene).^22^ In addition, second overall quasi-reversible one-electron reductions were observed for **1b** and **2b** within the electrochemical window of CH_2_Cl_2_, both at −1.67 V vs Fc^+/0^. Using THF as a solvent allowed observation of second, irreversible one-electron reductions for **1a**, **2a**, and **3** with peak maxima at −1.90 V, −1.87 V, and −1.80 V vs Fc^+/0^, respectively. Redox amphoterism of PAAHs has implications on ambipolar charge-carrier transport. Notably, quasi-reversible one-electron oxidations were measured for **1b**, **2b**, and **3** in CH_2_Cl_2_ at 0.92, 0.91, and 0.67 V vs Fc^+/0^, respectively. Irreversible oxidation events were observed for **1a** and **2a**, with peak potentials at 0.63 and 0.66 V, respectively. From voltammetric data, HOMO–LUMO energy gaps ranging 2.06–2.19 eV were estimated for **1**–**3** (Table 1).

### Crystallographic Studies

Pyracylenes **1a**–**b**, **2a**–**b**, and **3** were studied by X-ray crystallography (Figures 4 and S53). The pyracylene cores of each of these compounds are planar with only slight deviations. For example, **1a** shows a slight curvature with planes defined by 5-membered rings deviating ∼8.6° from coplanarity (Figure S50), and **3** shows a torsion angle of ∼3.5° about the central C=C bond (Figure S51). In all cases, significant bond-length alternations are observed between carbons of the pyracylene cores, consistent with the reported solid-state structure of unsubstituted pyracylene.^14^ Thus, perimeter C–C bonds of **1**–**3** alternate between shorter lengths <1.41 Å (e.g., Figure 4a,d,g, bonds labeled a, c and e), and longer lengths >1.44 Å (e.g., Figure 4a,d,g, bonds labeled b, d and f). The central C–C bonds of **1**–**3** (e.g., Figure 4a,d,g, bonds labeled h) are short, with bond lengths resembling C–C double bonds (1.32 Å–1.35 Å). The optimized structures from DFT calculations agree well with experimental results in terms of both bond lengths and patterns of bond alternation, although the central C=C bond lengths of **1b** and **2b** were about 0.01 Å longer than the experimental results (Figure S54).

**Figure 4:**
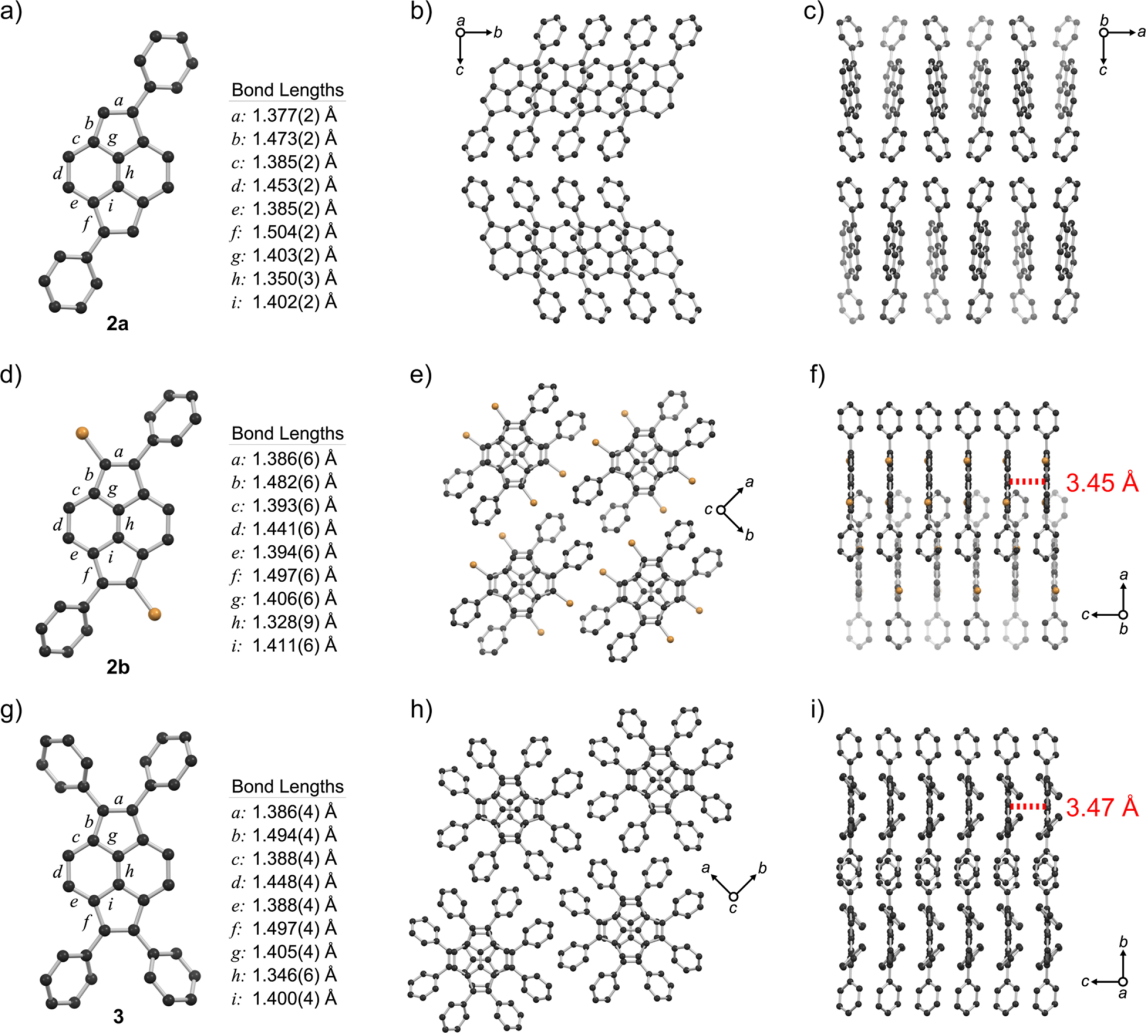
Solid state structures of pyracylenes **2a**, **2b**, and **3** highlighting molecular structures and bond lengths (a: **2a**, d: **2b**, g: **3**), as well as solid-state packing arrangements (b,c: **2a**, e,f: **2b**, h,i: **3**). C: black, Br: orange, H atoms omitted for clarity.

Strikingly, pyracylenes **1b**, **2b**, and **3** arrange into continuous 1D π-stacks with unusual packings of polycyclic antiaromatic cores, exhibiting 90° twist angles and negligible parallel displacements between adjacent molecules (Figures 4e,f,h,i, and S53e,f). Equal spacings between pyracylene cores were calculated with centroid–mean plane distances of 3.41 Å (**1b**), 3.45 Å (**2b**), and 3.47 Å (**3**). This structural characteristic differs from **1a** and **2a**, which do not form π-stacks. The unique, face-to-face 1D stacking of **1b**, **2b**, and **3** also differs from previously reported structures of pyracylenes.^13c,14,17^ The packings of **1b**, **2b**, and **3** contrast with the usual parallel-displaced π-stacks observed for aromatic molecules, where displacement mitigates repulsive interactions. Indeed, packings of **1b**, **2b**, and **3** can be compared to (+), or “Greek cross,” aggregate structures of aromatic π-systems, for which very few examples have been crystallographically characterized.^23^ In solid-state structures, close CH-π distances (<∼3.0 Å) were observed between orthogonally oriented phenyl substituents on neighboring pyracylene molecules (Figure S52). These occur between adjacent molecules within 1D π-stacks of **1b**, **2b**, and **3**, and additionally between molecules in adjacent stacks of **1b** and **3**.

### Analysis of Aromaticity and Stacking Interactions

The intermolecular interactions in the 90°-twisted stacking system were analyzed using DFT calculations. The dimeric part was extracted from the solid-state structure of **2b**, and the energy decomposition analysis (EDA) for the intermolecular interaction energy was conducted (see Supporting Information). The absolutely localized MO (ALMO-)­EDA^24^ indicated that the dispersion term was the main contributor in the dimer of **2b**. On the other hand, the contribution of charge-transfer (CT) interactions related to orbital interactions were not significant due to the stacking distance of 3.45 Å, where orbital overlap is limited.

Next, we constructed a face-to-face dimer model based on the optimized structure of unsubstituted pyracylene and examined the potential energy surface for the stacking distance *d* and twist angle θ (Figure 5a). The results indicated a local minimum around θ = 90° and *d* ∼ 3.4 Å (Figure 5b,c), resembling the orientations of **1b**, **2b**, and **3** in the solid state. In this orientation, the orbital interactions between the HOMOs (LUMOs) of the pyracylene dimer vanish (Figure 5d), suggesting that the destabilization of the HOMO caused by π-stacking is minimal, while dispersion forces provide significant stabilization. This is an interesting divergence from typical aromatic π-stacks, wherein parallel displacements mitigate repulsions.

**Figure 5:**
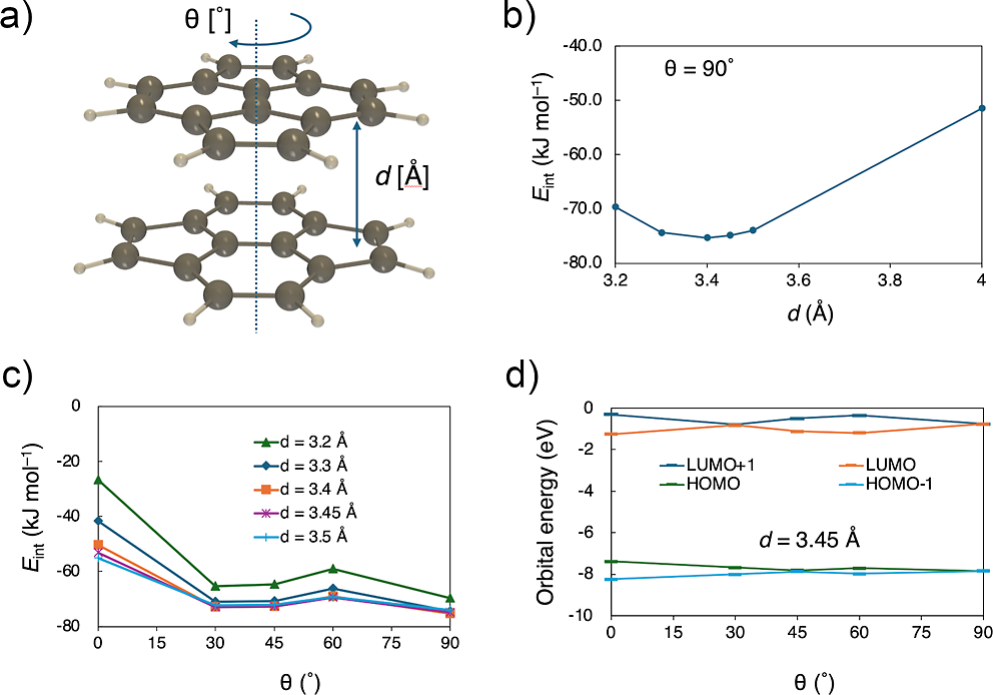
Structure of unsubstituted pyracylene dimer model (a), *d*-dependence of the interaction energy *E*_int_ for models with θ = 90° (b), θ-dependence of *E*_int_ (c), and θ-dependence of the orbital energies for models with *d* = 3.45 Å (d), calculated at the ωB97M-V/def2-SVPD level.^25^

Although the orbital interaction between the HOMOs (LUMOs) of monomers vanishes at θ = 90°, that between the HOMO of one monomer and the LUMO of the other monomer does not vanish. Interestingly, at this angle, the phase of the HOMO of one molecule matches that of the LUMO of the other molecule (Figure 6). Although this effect appears to be small at the stacking distance observed in the solid-state structures of **1b**, **2b**, and **3** (*d* ∼ 3.45 Å), to our knowledge there are no reports of this type of orbital interaction mode in one-dimensional π-stacks of antiaromatic molecules. Our calculations indicate that this HOMO–LUMO orbital interaction between pyracylene monomers should become important at shorter stacking distances (Figure S61), suggesting future design considerations for stabilization of antiaromatic π-stacked structures. For example, molecular designs of pyracylene which reduce HOMO–LUMO gaps may strengthen such orbital interactions and reduce stacking distances. Here it is worth contrasting with aromatic compounds, which are typified by HOMOs and LUMOs of different symmetry where the LUMO is bisected by an additional nodal plane compared to the HOMO. Thus, due to phase considerations, the face-to-face HOMO–LUMO interaction of two identical aromatic π-surfaces may be expected to be net nonbonding at any rotation of neighboring molecules.

**Figure 6:**
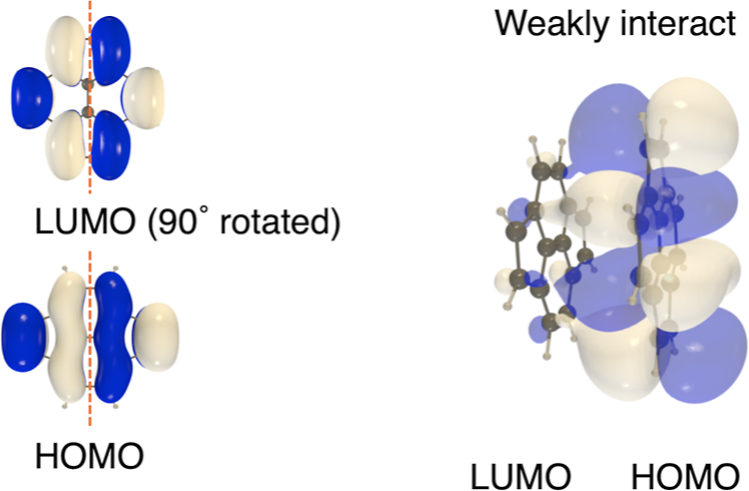
Diagrammatic representation of the orbital interaction between the HOMO of one pyracylene monomer and the LUMO of another pyracylene monomer at θ = 90° and *d* = 3.45 Å.

Magnetic response properties were evaluated at the GIAO-RB3LYP/6-311++G­(d,p) level (see Supporting Information). The geometries were taken from the solid-state structure and XY-scans of NICS_π*zz*,σ‑only_ values were evaluated 1.7 Å above the molecular plane (Figures 7 and S58).^26^ Consistent with previous calculations of unsubstituted pyracyclene,^11^ positive NICS_π*zz*,σ‑only_ values indicated paratropicity primarily localized on five-membered rings of **1–3**.

**Figure 7:**
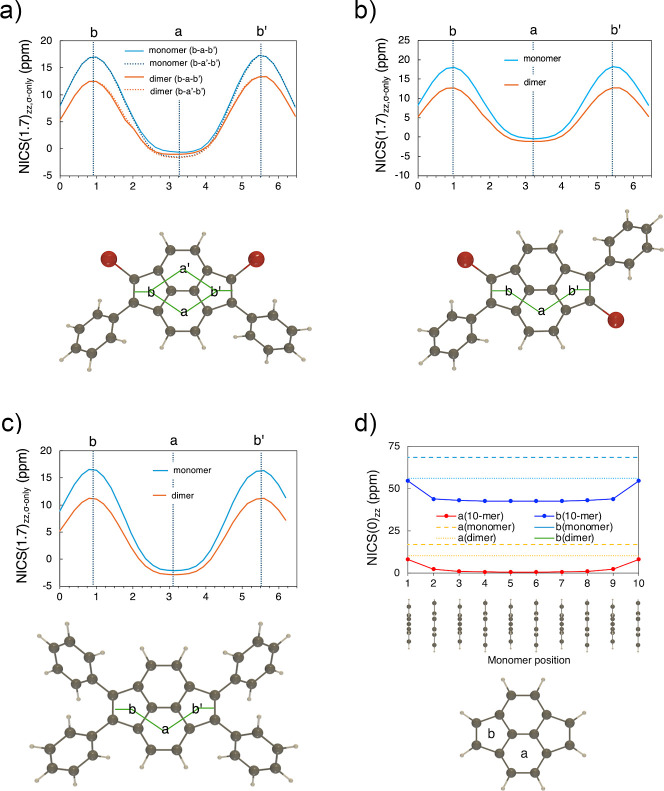
Results of NICS-XY scan of the monomer and π-stacked dimer of **1b** (a), **2b** (b), and **3** (c), with geometries based on solid-state structures, and (d) NICS(0)_*zz*_ of the 10-mer model consisting of unsubstituted pyracylenes with *d* = 3.45 Å and θ = 90°, calculated at the GIAO-RB3LYP/6-311++G­(d,p) level.

We then assessed the effect of intermolecular interactions on NICS_π*zz*,σ‑only_ values of **1b**, **2b**, and **3** by using dimer models with geometries extracted from solid-state structures. For the dimers, the NICS(1.7)_π*zz*,σ‑only_ was scanned outside the dimer structure (Figure 7a–c). The NICS(1.7)_π*zz*,σ‑only_ values of the five-membered rings in the dimeric structures were diminished compared to those in the monomer, indicating that intermolecular interactions between pyracylenes in this stacking arrangement could reduce the paratropicity of five-membered rings to some extent and increase the diatropicity of six-membered rings slightly.

Nevertheless, **1b**, **2b**, and **3** are shown to form infinite one-dimensional π-stacking structures with uniform stacking distances (*d*) in the solid state. To explore the effect of such continuous, face-to-face, 90°-twist-angle stacking arrangements on (anti)­aromaticity, NICS calculations were performed for a 10-mer model consisting of unsubstituted pyracylenes with *d* = 3.45 Å and θ = 90°. NICS(0)_*zz*_ values were evaluated at the centers of the five- and six-membered rings of each monomer (Figure 7d). The NICS(0)_*zz*_ values in the middle region of the 10-mer were almost converged.

The converged value on the five-membered ring (∼42.6 ppm) was significantly decreased from the NICS(0)_*zz*_ value of the monomer (68.5 ppm), and even smaller than that of a similarly assessed dimer model (56.0 ppm). These results suggest that antiaromatic character also weakens in the continuous stacking case of pyracylene with face-to-face 90°-twist-angle arrangements of monomers.

The reduction of antiaromatic character caused by molecular stacking has been previously discussed using a mechanism involving orbital level switching due to strong interactions between the HOMOs (LUMOs) for fully face-to-face (θ ∼ 0° and *d* ∼ 3 Å) dimers of cyclobutadiene and Ni­(II) norcorrole.^6,7^ Intriguingly, paratropicity reduction in face-to-face, 90°-twist-angle arrangements of pyracylenes, with orthogonally oriented HOMOs (LUMOs) and vanishing HOMO–HOMO (LUMO–LUMO) interactions, evidently does not follow this mechanism. For the pyracylenes computationally examined herein, we propose that the interactions of the HOMO of one monomer and the LUMO of another monomer, although weak at *d* ∼ 3.45 Å, influence magnetic response properties. Such interactions are expected to increase the HOMO–LUMO gap (Δε_HL_ = ε_LUMO_ – ε_HOMO_) in the dimer (or *n*-mer) compared to that of the monomer (Figure S60). Noting the molecular orbital contribution to magnetic responses (see Supporting Information), these changes to Δε_HL_ can in turn be expected to affect NICS values. Indeed, the NICS_*zz*_ values for five-membered rings of unsubstituted pyracylene monomers and 90°-twisted π-stacking dimers at *d* = 3.45 Å and 10.0 Å show correspondence to Δε_HL_ (Table S7). Furthermore, a canonical MO-based decomposition analysis of the NICS (CMO-NICS)^26a^ enabled us to ascribe the trend of total NICS_*zz*_ to trends in the contribution from the degenerate HOMO and HOMO–1. These results suggest that the intermolecular HOMO–LUMO orbital interactions between 90°-twisted π-stacked pyracylenes lead to an increase of Δε_HL_ which in turn reduces paratropicities (see Supporting Information, Table S7).

### Conductivity Measurements

Motivated by emerging applications of PAAHs in electronic materials,^5^ we measured time-resolved microwave conductivity (TRMC) profiles^27^ of crystalline samples of **1–3** (Figure 8). Crystalline samples of **1b**, **2b**, and **3**, which possess 1D π-stacking channels, exhibited clear TRMC signals with φΣμ ≈ 7 × 10^–9^ m^2^/(V s) comparable to those of typical π-conjugated polymers.^28^ To compare with other antiaromatic compounds, while one specific derivative in antiaromatic norcorrole liquid crystals was reported to exhibit a relatively high φΣμ_max_ value of up to 5.2 × 10^–8^ m^2^/(V s),^29^ most reported antiaromatic systems show φΣμ_max_ values on the order of ∼0.1–1 × 10^–9^ m^2^/(V s).^30^ The photoconductivities observed in our systems are comparable in magnitude to these typical values. Interestingly, no orbital interactions between the HOMOs (LUMOs) of adjacent monomers of unsubstituted pyracylene are expected in 90°-twisted stacking arrangements. Nevertheless, observed conductivities of **1b**, **2b**, and **3** may be explained by substituent-induced symmetry reduction of frontier molecular orbitals that allows electronic coupling between neighboring stacked molecules (see Supporting Information), or by conducting pathways other than the stacking direction. Crystals of **1a** and **2a**, which possess herringbone arrangements of pyracylene π-surfaces in the solid state, also exhibited TRMC signals comparable to those of **1b**, **2b**, and **3**.

**Figure 8:**
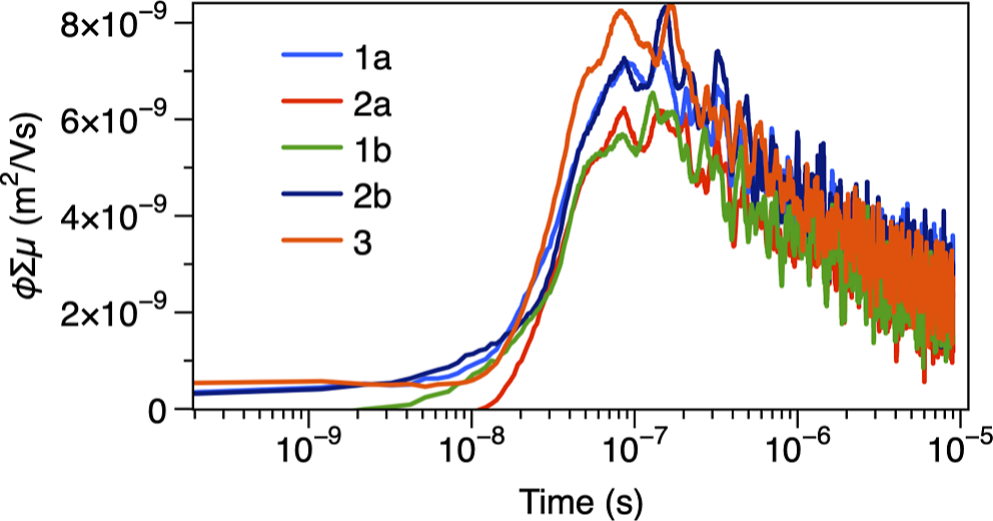
TRMC profiles of **1a** (blue), **2a** (red), **1b** (green), **2b** (deep blue), and **3** (orange).

## Conclusions

An expedient solution-phase synthesis of pyracylenes was achieved through Pd-catalyzed ring contraction of diborinic acids. The resulting pyracylenes underwent further derivatization via bromination and Pd-catalyzed cross-coupling reactions. All pyracylene products were fully characterized by ^1^H and ^13^C NMR spectroscopy, high-resolution mass spectrometry, and single-crystal X-ray crystallography. The electronic, optical, and magnetic properties of these products were studied using UV–vis absorption spectroscopy, cyclic voltammetry, and DFT calculations. Despite characteristic antiaromatic properties revealed by these studies, the pyracylenes were stable under ambient conditions.

Three pyracylene derivatives synthesized herein assembled into face-to-face one-dimensional antiaromatic π-stacks in the solid state, featuring uniform intermolecular distances and 90° twist angles between adjacent monomers. DFT calculations indicated that this stacking arrangement aligns HOMOs of antiaromatic monomers with phase-complementary LUMOs of neighboring molecules (and vice versa) while diminishing antiaromaticities of individual pyracylene molecules. Time-resolved microwave conductivity measurements revealed conductivities for pyracylenes in the solid state.

These results provide renewed access to a fundamental and intriguing nonalternant hydrocarbon scaffold, and have revealed unique characteristics of pyracylene that we hope will further understanding of antiaromatic π-stacking and present new prospects for antiaromatic materials design.

## Supplementary Material



## Notes

Experimental details on synthetic procedures, NMR characterization, UV–vis spectroscopy, cyclic voltammetry, X-ray crystallography, computations, and TRMC studies (PDF)
