# Supplementary material for: Pyracylenes: Facile Synthetic Access and Continuous Face-To-Face Antiaromatic π‑Stacking
Source: JACS Au. 2026 Jan 19;6(2):1118–28. doi: 10.1021/jacsau.5c01515 (PMC12933335; doi:10.1021/jacsau.5c01515)
Supplement: Supplementary file 1 [file au5c01515_si_001.pdf]

Supporting Information

Pyracylenes: Facile Synthetic Access and Continuous Face-to-Face Antiaromatic π-Stacking

Sheng-Yuan Cheng,[a] Jiun-Siang Juang,[a] Yu-Fang Huang,[a] Ting-Yi Lai,[a] Yi-Hung Liu,[a] Fumitaka Ishiwari,[b, c] Akinori Saeki,[b, d] Kenji Okada,[e] Ryohei Kishi,*[, d, e, f] and Jeffrey M. Farrell*[, a, g]

[a] Department of Chemistry, National Taiwan University, No. 1, Sec. 4, Roosevelt Rd., Taipei 10617, Taiwan.
[b] Department of Applied Chemistry, Graduate School of Engineering, Osaka University, Suita, Osaka 565-0871, Japan
[c] Graduate School of Urban Environmental Sciences, Tokyo Metropolitan University, 1-1 Minamiosawa, Hachioji, Tokyo 192-0297, Japan.
[d] Innovative Catalysis Science Division, Institute for Open and Transdisciplinary Research Initiatives (ICS-OTRI), The University of Osaka, 2-1 Yamadaoka, Suita, Osaka 565-0871, Japan.
[e] Graduate School of Engineering Science, The University of Osaka, 1-3 Machikaneyama, Toyonaka, Osaka 560-8531, Japan.
[f] Center for Quantum Information and Quantum Biology, The University of Osaka, 1-3 Machikaneyama, Toyonaka, Osaka 560-8531, Japan.
[g] Center for Emerging Materials and Advanced Devices, National Taiwan University, No. 1, Sec. 4, Roosevelt Rd., Taipei 10617, Taiwan.

Table of Contents

1. **Materials and Methods** 2
2. **Experimental Procedures** 3
3. **NMR Spectra** 15
4. **Simulated and measured HR-MS spectra** 25
5. **UV–Vis Spectroscopy** 27
6. **Cyclic Voltammetry** 32
7. **X-ray Crystallography** 40
8. **DFT calculations** 46
9. **References** 68

1. Materials and Methods

General considerations. Where indicated, glovebox synthetic manipulations were carried out in an atmosphere of dry, O_2-free N_2 in an MBraun glovebox using oven-dried glassware. 1,6-dihydroxy-2,7-diphenyl-1,6-diborapyrene and 1,8-dihydroxy-2,7-diphenyl-1,8-diborapyrene were prepared according to literatures reports.[1] Deuterated solvents were obtained from commercial sources and used without further purification. All other solvents for spectroscopic measurements were spectroscopic grade and used without further purification. Column chromatography was performed with commercial columns using silica gel (0.065 mm). All other reagents and solvents were obtained from commercial sources and used without further purification.

UV-Vis absorption spectra were recorded on a JASCO V-770 spectrophotometer.

NMR spectra were recorded on a Bruker AVIIIHD 400 MHz FT-NMR or a Bruker AVIIIHD 500 MHz FT-NMR spectrometer. Chemical shifts are listed in parts per million and are given relative to $SiMe_4$ and referenced to a residual solvent signal (^{1}H, ^{13}C). Coupling constants (*J*) are quoted in Hertz (Hz).

High resolution mass spectrometry experiments were performed on a Bruker Daltonic Autoflex Speed or Bruker ultrafleXtreme instrument.

Cyclic voltammetry experiments were performed using a commercial electrochemical analyzer (CHI621E, CH Instrument, USA) with a three-electrode single-compartment cell. The supporting electrolyte tetrabutylammonium hexafluorophosphate (n-Bu_4NPF_6) was purchased from Combiblock and used without further purification. The measurements were recorded using ferrocene (Fc) as an internal standard for the calibration of the potential. An Ag/AgCl reference electrode was used. A Pt disc and a Pt wire were used as working auxiliary electrodes, respectively.

Sublimation was performed using a TENDER (TF-12-95-900) vacuum deposition system comprising a three-zone tube furnace and fitted turbomolecular vacuum pump. Under vacuum (10^{-5} torr), compounds sublimed at 110 °C (**1a**), 140 °C (**2a**), 110 ºC (**1b**), 140 °C (**2b**), and 160 °C (**3**).

Single crystal X-ray diffraction data were recorded at 100 K on a Bruker D8 Venture SC-XRD with a Photon III C28 detector and multi-layered mirror monochromated CuKα radiation. The structures were solved using Shelxt methods, expanded with Fourier techniques and refined with the Shelxt software package.[2] All non-hydrogen atoms were refined anisotropically. Hydrogen atoms were included in the structure

factor calculation on geometrically idealized positions. Crystallographic data have been deposited with the Cambridge Crystallographic Data Centre under entries no. 2492995-2492999 These data can be obtained free of charge from The Cambridge Crystallographic Data Centre via www.ccdc.ac.uk/data.request/cif.

DFT calculations were performed using Gaussian 16 program package,[3] unless otherwise noted. First, geometries of **1a**, **1b**, **2a**, **2b**, and **3**, as well as unsubstituted pyracylene, were optimized at the spin-restricted (R)CAM-B3LYP/6-311G(d,p)[4] level. Frequency analysis calculation was performed to confirm the local minimum geometry.

Time-resolved microwave conductivity (TRMC) data were obtained by the following procedure. The sample adhered to adhesive tape was excited using a third harmonic (355 nm) of a Nd:YAG laser (Continuum, Surelite II, 5–8 ns pulse duration, 10 Hz) with an incident photon density (I_0) of 9.1×10^{15} photons cm^{-2} $pulse^{-1}$, where the microwave frequency and power were ≈9 GHz and ≈3 mW, respectively. TRMC signals represent a product of the quantum yield (φ) and the sum of the charge-carrier mobilities $\Sigma\mu$ ($= \mu_+ + \mu_-$) converted from the photoconductivity ($\Delta\sigma = A^{-1}\cdot\Delta P_r\cdot P_r^{-1}$, where A is the sensitivity factor, P_r is the reflected microwave power, and ΔP_r is the change in P_r upon exposure to light) using the equation $\varphi\Sigma\mu = \Delta\sigma(eI_0F_{light})^{-1}$, where e and F_{light} are the electron charge and correction (or filling) factor, respectively. The experiments were performed at room temperature in the air.

2. Experimental Procedures

NMR spectroscopic monitoring of the formation of 1a by stoichiometric Pd-mediated ring contraction of I.

In an inert atmosphere glove box, 1,8-dihydroxy-2,7-diphenyl-1,8-diborapyrene (**I**, 10.0 mg, 0.026 mmol) in 0.6 mL CD_3CN was added to a J-Young NMR tube. After measurement of an ^1H NMR spectrum, a solution of $Pd(OAc)_2$ (11.7 mg, 200 mol%) in 0.1mL CD_3CN was added into the J-Young tube. The reaction was monitored by ^1H NMR spectroscopy before heating, and after heating 70 °C at 0.5 hours and 2 hours. (**Figure S1**).

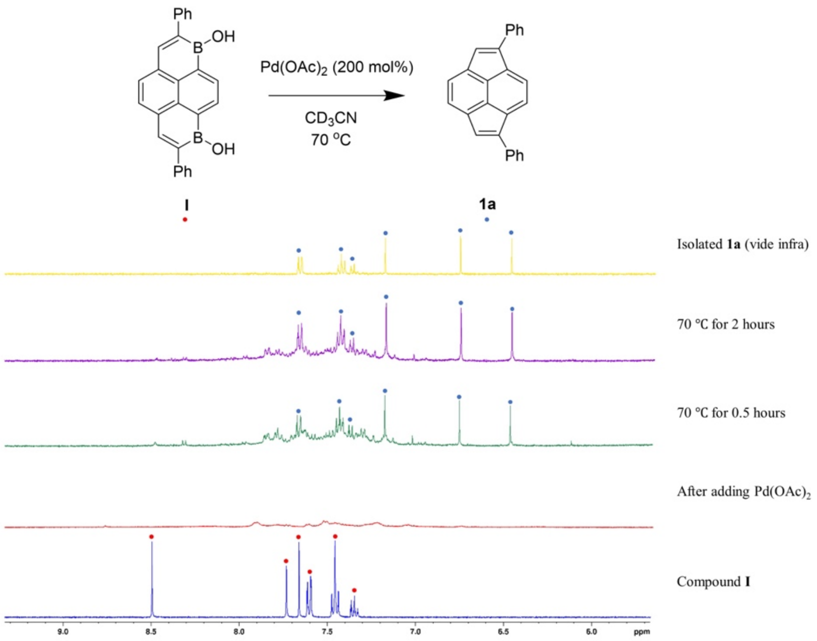

Figure S1. Magnified aromatic region of ^{1}H NMR spectra of monitoring the stoichiometric palladium ring contraction reaction (400 MHz, CD_3CN, 298 K).

Preparative-scale synthesis of 1a by stoichiometric Pd-mediated ring contraction of I.

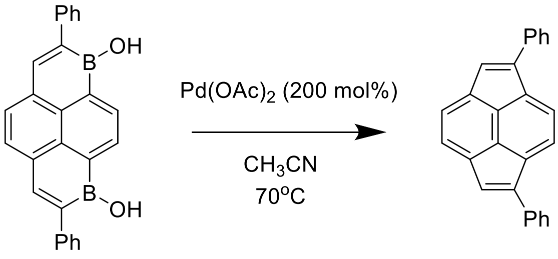

11%

1,8-dihydroxy-2,7-diphenyl-1,8-diborapyrene (50.0 mg, 0.130 mmol) and $Pd(OAc)_2$ (58.5 mg, 0.261 mmol) were weighed into a 10 mL Schlenk tube fitted with a magnetic stir bar and sealed with a Teflon cap. The Schlenk tube was evacuated and backfilled with nitrogen three times. Acetonitrile (3.0 mL) was added via micropipette under positive N_2 pressure and the reaction mixture was stirred at 70 °C for 0.5 hours over which time the solution turned deep green. Volatiles were removed in vacuo, and the residue was purified by column chromatography (eluent: 10:1 n-hexane : DCM). Pure 1,6-diphenylpyracylene (4.8mg, 0.015 mmol, 11%) was isolated as a green solid following solvent removal by high vacuum and was spectroscopically identical to **1a** isolated under optimized conditions, vide infra.

Optimization of Pd-catalyzed ring contraction of I to form 1a:

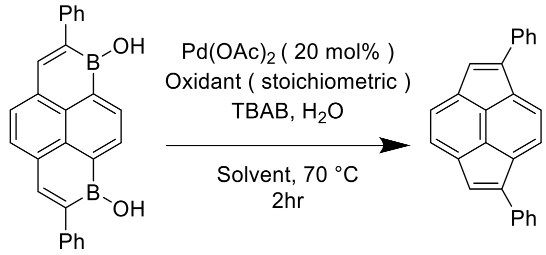

General Procedure: 1,8-dihydroxy-2,7-diphenyl-1,8-diborapyrene (50.0 mg, 0.130 mmol) was weighed into a 10 mL Schlenk tube fitted with a magnetic stir bar and sealed with a Teflon cap. The Schlenk tube was evacuated and backfilled with nitrogen three times where N_2 atmosphere is indicated (Table S1). To the Schlenk tube was added 3.0 mL solvent and, where indicated, [n-Bu_4N][Br] $_{(aq)}$ or water via micropipette under positive N_2 pressure. $Pd(OAc)_2$ (5.5 mg, 0.024 mmol), and oxidant (stoichiometric) were dissolved or suspended in 2.0 mL solvent and this mixture was added via micropipette under positive N_2 pressure. The reaction mixture was stirred at 70 °C for 2 hours. Volatiles were removed in vacuo and the residue was purified by column chromatography (eluent: 10:1 n-hexane : DCM). Pure 1,6-diphenylpyracylene was isolated as a green solid following solvent removal under high vacuum, and was spectroscopically identical to **1a** isolated under optimized conditions, vide infra (Table S1).

Table S1. Optimization of Oxidative Palladium-Catalyzed C−C coupling for Pyracylene.

Entry	Solvent	Oxidant	[n-Bu_4N][Br]	H_2O	Atmosphere	Yield[a]
**1**	CH_3CN	1,4-benzoquinone	X	X	air	14%
**2**	CH_3CN	1,4-benzoquinone	X	0.5 mL	air	9%
**3**	CH_3CN	1,4-benzoquinone	1 equiv.	0.5 mL	air	11%
**4**	CH_3CN	1,4-benzoquinone	5 equiv.	0.5 mL	air	34%
**5**	CH_3CN	1,4-benzoquinone	10 equiv.	0.5 mL	air	35%
**6**	CH_3CN[b]	1,4-benzoquinone	10 equiv.	0.5 mL	N_2	48%
**7**	CH_3CN	1,4-benzoquinone	10 equiv.	0.5 mL	N_2	54%
**8**	Toluene	1,4-benzoquinone	10 equiv.	0.5 mL	N_2	56%
**9**	DMF	1,4-benzoquinone	10 equiv.	0.5 mL	N_2	27%
**10**	DMSO	1,4-benzoquinone	10 equiv.	0.5 mL	N_2	12%
**11**	Dioxane	1,4-benzoquinone	10 equiv.	0.5 mL	N_2	34%
**12**	Toluene	$Cu(OAc)_2$	10 equiv.	0.5 mL	N_2	20%
**13**	Toluene	AgOAc	10 equiv.	0.5 mL	N_2	<1%
**14**	Toluene	TEMPO	10 equiv.	0.5 mL	N_2	22%
**15**	Toluene	MnO_2	10 equiv.	0.5 mL	N_2	3%

[a] Isolated yield. [b] Degassed with three freeze-pump-thaw cycles.

Synthesis of 1,6-diphenylpyracylene (1a)

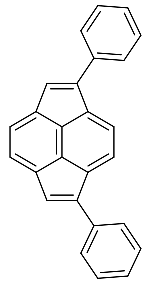

1,8-dihydroxy-2,7-diphenyl-1,8-diborapyrene (300.0 mg, 0.781 mmol) was weighed into a 100 mL Schlenk flask fitted with a magnetic stir bar and sealed with a rubber septum. The Schlenk flask was evacuated and backfilled with nitrogen three times. To the flask was added 6.0 mL toluene and $[n\text{-}Bu_4N][Br]_{(aq)}$ (2.8 g, 8.8 mmol $[n\text{-}Bu_4N][Br]$ dissolved in 3.0 mL H_2O) via micropipette under positive N_2 pressure. $Pd(OAc)_2$ (35.1 mg, 0.156 mmol) and 1,4-benzoquinone (168.8 mg, 1.56 mmol) were dissolved in 12.0 mL toluene, and the solution was added into the tube via micropipette under positive N_2 pressure. The reaction mixture was stirred at 70 °C for 2 hours, over which time the solution turned deep green. All volatiles were removed in vacuo. The residue was purified by column chromatography (eluent: 10:1 n-hexane : DCM). Pure 1,6-diphenylpyracylene (138.8 mg, 0.423 mmol, 54.2 % yield) was isolated as a green solid following solvent removal by high vacuum.

^{1}H NMR (400 MHz, CD_2Cl_2, 298 K): δ 7.64-7.61 (m, 4H), 7.44-7.40 (m, 4H,), 7.36-7.32 (m, 2H), 7.10 (s, 2H), 6.70 (s, 2H), 6.38 (s, 2H).

^{13}C{^{1}H} NMR (125MHz, CD_2Cl_2, 298 K): δ 147.2 (C), 142.2 (C), 142.0 (C), 135.7 (C), 133.5 (C), 129.2 (C), 128.7 (C), 128.4 (C), 126.8 (C), 125.9 (C), 125.6 (C).

HR-MS (MALDI-TOF, positive mode) *m/z*: $[M]^+$ Calc'd for $C_{26}H_{16}$ 328.1246; Found 328.1256.

CV (1.79 x 10^{-4} M, 0.1 M $n\text{-}Bu_4NPF_6$, in CH_2Cl_2, vs. $Fc^{+/0}$, 298 K): $E_{1/2\ red\ 1}$ = -1.47 V. (2.0 x 10^{-4} M, 0.1 M $n\text{-}Bu_4NPF_6$, in THF, vs. $Fc^{+/0}$, 298K): $E_{1/2\ red\ 1}$ = -1.50 V, $E_{1/2\ red\ 2}$ = -1.90 V.

UV-Vis (5.6 x 10^{-5} M in CH_2Cl_2, 298 K): λ_{max} (ε_{max}) = 360 nm (13600), 377 nm (11100), 600 nm (1200).

Synthesis of 1,5-diphenylpyracylene (2a)

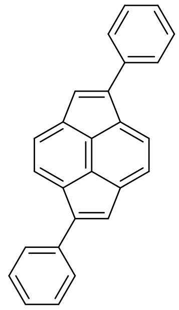

1,6-dihydroxy-2,7-diphenyl-1,6-diborapyrene (300.0 mg, 0.781 mmol) was weighed into a 25 mL Schlenk flask fitted with a magnetic stir bar and sealed with a rubber septum. The Schlenk flask was evacuated and backfilled with nitrogen three times. To the flask was added 6.0 mL acetonitrile and $[n\text{-}Bu_4N][Br]_{(aq)}$ (2.8 g, 8.8 mmol $[n\text{-}Bu_4N][Br]$ dissolved in 3.0 mL H_2O) via micropipette under positive N_2 pressure. $Pd(OAc)_2$ (35.1 mg, 0.156 mmol) and 1,4-benzoquinone (168.8 mg, 1.56 mmol) were dissolved in 12.0 mL acetonitrile, and the solution was added into the tube via micropipette under positive N_2 pressure. The reaction mixture was stirred at 70 °C for 2 hours, over which time the solution turned deep green, and a black solid precipitated from the solution. The solid was filtered and washed with MeOH, and n-hexane. The solid was then dissolved in CH_2Cl_2 and filtered through a Celite pad. Pure 1,5-diphenylpyracylene (212.1 mg, 0.646 mmol, 82.7 % yield) was isolated as a black, green solid following solvent removal by high vacuum.

^{1}H NMR (400 MHz, CD_2Cl_2, 298 K): δ7.67-7.65 (m, 4H,), 7.47-7.43 (m, 4H), 7.40-7.36 (m, 2H,), 7.07 (d, 2H, $^3J_{HH}$ = 6.83 Hz), 6.80 (d, 2H, $^3J_{HH}$ = 6.83 Hz), 6.41 (s, 2H).

^{13}C{^{1}H} NMR (125MHz, CD_2Cl_2, 298 K): δ 147.6 (C), 142.7 (C), 141.2 (C), 135.5 (C), 133.3 (C), 129.0 (C), 128.6 (C), 127.8 (C), 126.7 (C), 126.6 (C), 124.6 (C).

HR-MS (MALDI-TOF, positive mode) *m/z*: $[M]^+$ Calc'd for $C_{26}H_{16}$ 328.1246; Found 328.1241.

CV (2.2 x 10^{-4} M, 0.1 M n-Bu_4NPF_6, in CH_2Cl_2, vs. $Fc^{+/0}$, 298 K): $E_{1/2\ red\ 1}$ = -1.47 V. (1.4 x 10^{-4} M, 0.1 M n-Bu_4NPF_6, in THF, vs. $Fc^{+/0}$, 298K): $E_{1/2\ red\ 1}$ = -1.50 V, $E_{1/2\ red\ 2}$ = -1.90 V.

UV-Vis (2.1x 10^{-5} M in CH_2Cl_2, 298 K): λ_{max} (ε_{max}) = 365 nm (13600), 384 nm (13000), 417 nm (3700), 436 nm (3400), 514nm (400).

Synthesis of 1,6-dibromo-2,5-diphenylpyracylene (1b)

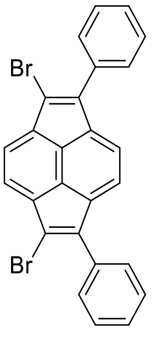

In a glove box filled with N_2, 1,6-diphenylpyracylene (30.0 mg, 0.0913 mmol) was dissolved in 3 mL $CHCl_3$ in a 20 mL vial. NBS (32.5 mg, 0.183 mmol) was added at -35 °C. After stirring for 3 hours, all volatiles were removed in vacuo. The residue was washed with MeOH. Pure 1,6-dibromo-2,5-diphenylpyracylene (40.2 mg, 0.0827 mmol, 90.6 % yield) was isolated as a red solid following solvent removal by high vacuum.

^{1}H NMR (400 MHz, $CDCl_3$, 298 K): δ 7.64-7.61 (m, 4H,), 7.47-7.43 (m, 4H), 7.41-7.36 (m, 2H,), 6.80 (s, 2H), 6.73 (s, 2H).

^{13}C{^{1}H} NMR (125 MHz, $CDCl_3$, 298 K): δ 142.9 (C), 141.9 (C), 140.9 (C), 133.0 (C), 130.2 (C), 128.5 (C), 128.4 (C), 128.3 (C), 124.9 (C), 124.4 (C), 119.9 (C).

HR-MS (MALDI-TOF, positive mode) *m/z*: $[M]^+$ Calc'd for $C_{26}H_{14}Br_2$ 485.9438; Found 485.9434.

CV (1.6×10^{-4} M, 0.1 M n-Bu_4NPF_6, in CH_2Cl_2, vs. $Fc^{+/0}$, 298 K): $E_{1/2\ ox\ 1}$ = 0.922 V, $E_{1/2\ red\ 1}$ = -1.26 V, $E_{1/2\ red\ 2}$ = -1.67 V.

UV-Vis (3.3×10^{-5} M in CH_2Cl_2, 298 K): λ_{max} (ε_{max}) = 365 nm (22700), 383 nm (18300), 572 nm (500).

Synthesis of 1,5-dibromo-2,6-diphenylpyracylene (2b)

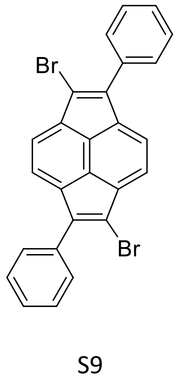

In a glove box filled with N_2, 1,5-diphenylpyracylene (30.0 mg, 0.0913 mmol) was dissolved in 3 mL $CHCl_3$ in a 20 mL vial. NBS (32.5 mg, 0.183 mmol) was added at -35 °C. After stirring for 3 hours, all volatiles were removed in vacuo. The residue was washed with MeOH. Pure 1,5-dibromo-2,6-diphenylpyracylene (37.8 mg, 0.0777 mmol, 85.1 % yield) was isolated as a red solid following solvent removal by high vacuum.

^{1}H NMR (400 MHz, $CDCl_3$, 298 K): δ7.65-7.63 (m, 4H,), 7.48-7.44 (m, 4H), 7.41-7.38 (m, 2H,), 6.83 (d, 2H, $^3J_{HH}$ = 6.81 Hz), 6.70 (d, 2H, $^3J_{HH}$ = 6.81 Hz)

^{13}C{^{1}H} NMR (125 MHz, $CDCl_3$, 298 K): δ 142.7 (C), 141.9 (C), 141.0 (C), 133.0 (C), 130.2 (C), 128.5 (C), 128.5 (C), 128.3 (C), 125.0 (C), 124.3 (C), 120.1 (C)

HR-MS (MALDI-TOF, positive mode) *m/z*: $[M]^+$ Calc'd for $C_{26}H_{14}Br_2$ 485.9438; Found 485.9460.

CV (1.03×10^{-4} M, 0.1 M n-Bu_4NPF_6, in CH_2Cl_2, vs. $Fc^{+/0}$, 298 K): $E_{1/2\ ox\ 1}$ = 0.909 V, $E_{1/2\ red\ 1}$ = -1.28 V, $E_{1/2\ red\ 2}$ = -1.67 V.

UV-Vis (2.8×10^{-5} M in CH_2Cl_2, 298 K): λ_{max} (ε_{max}) = 364 nm (22300), 383 nm (17800), 444 nm (1400), 470 nm (800), 561 nm (400).

Synthesis of 1,2,5,6-tetraphenylpyracylene (3)

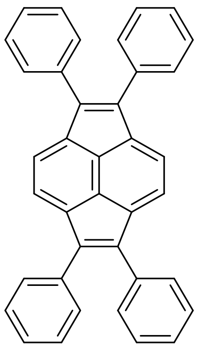

From 1b: Phenyl boronic acid (11.1 mg, 0.0910 mmol, 2.2 equiv.) and Cs_2CO_3 (133.9 mg, 0.411 mmol, 10 equiv.) were weighed into a 50 mL Schlenk flask equipped with a magnetic stir bar. The vessel was sealed with a rubber septum, evacuated, and backfilled with nitrogen gas. The flask was moved into the glovebox, then $Pd(PPh_3)_4$ (4.7 mg, 0.00411 mmol, 10 mol%) was added into the flask. 1,6-dibromo-2,5-diphenylpyracylene **1b** (20.0 mg, 0.0411 mmol, 1 equiv.) was dissolved in toluene (8 mL) then transferred into flask. The reaction mixture was stirred at 100 °C for 16 hours.

After the reaction cooled to room temperature, the precipitated red solid was filtered and washed with methanol. Pure 1,2,5,6-tetraphenylpyracylene (16.4 mg, 0.0341 mmol, 83 % yield) was isolated as a red solid by recrystallization from hot $CHCl_3$.

From 2b: Phenyl boronic acid (11.1 mg, 0.0904 mmol, 2.2 equiv.) and Cs_2CO_3 (133.9 mg, 0.411 mmol, 10 equiv.) were weighed into a 50 mL Schlenk flask equipped with a magnetic stir bar. The vessel was sealed with a rubber septum, evacuated, and backfilled with nitrogen gas. The flask was moved into the glovebox, then $Pd(PPh_3)_4$ (4.7 mg, 0.00411 mmol, 10 mol%) was added into the flask. 1,5-dibromo-2,6-diphenylpyracylene **2b** (20.0 mg, 0.0411 mmol, 1 equiv.) was dissolved in toluene (8 mL) then transferred into flask. The reaction mixture was stirred at 100 °C for 16 hours. After the reaction cooled to room temperature, the precipitated red solid was filtered and washed with methanol. Pure 1,2,5,6-tetraphenylpyracylene (16.3 mg, 0.0339 mmol, 82.5 % yield) was isolated as a red solid by recrystallization from hot $CHCl_3$.

^{1}H NMR (800 MHz, TCE-d_2, 333 K): δ 7.33-7.29 (m, 20H), 6.86 (s, 4H).
^{13}C {^{1}H} NMR (200 MHz, $CDCl_3$, 333 K): δ 143.3 (C), 141.3 (C), 135.0 (C), 131.9 (C), 128.9 (C), 128.3 (C), 127.5 (C), 124.9 (C).
HR-MS (MALDI-TOF, Positive mode) *m/z*: $[M]^+$ Calc'd for $C_{38}H_{24}$ 480.1873; Found 480.1910.
CV (1.3×10^{-4} M, 0.1 M n-Bu_4NPF_6, in CH_2Cl_2, vs. $Fc^{+/0}$, 298K): $E_{1/2\ ox\ 1}$ = 0.67 V, $E_{1/2\ red\ 1}$ = -1.45 V.
(8.3×10^{-5} M, 0.1 M n-Bu_4NPF_6, in THF, vs. $Fc^{+/0}$, 298K): $E_{1/2\ red\ 1}$ = -1.44 V, $E_{1/2\ red\ 2}$ = -1.80 V.
UV-Vis (4.8×10^{-5} M in CH_2Cl_2, 298 K): λ_{max} (ε_{max}) = 318 nm (18400), 370nm (16600), 387 nm (14000), 578 nm (300).

NMR spectroscopic monitoring of the stability of 1a, 1b, 2a, and 2b.

The powder of each pyracylene was stored in a vial under ambient conditions. After one week, the solid was analyzed by ^{1}H NMR spectroscopy. The solution of each compound was monitored daily by ^{1}H NMR spectroscopy until the emergence of decomposition peaks. (**Figure S2-S5**)

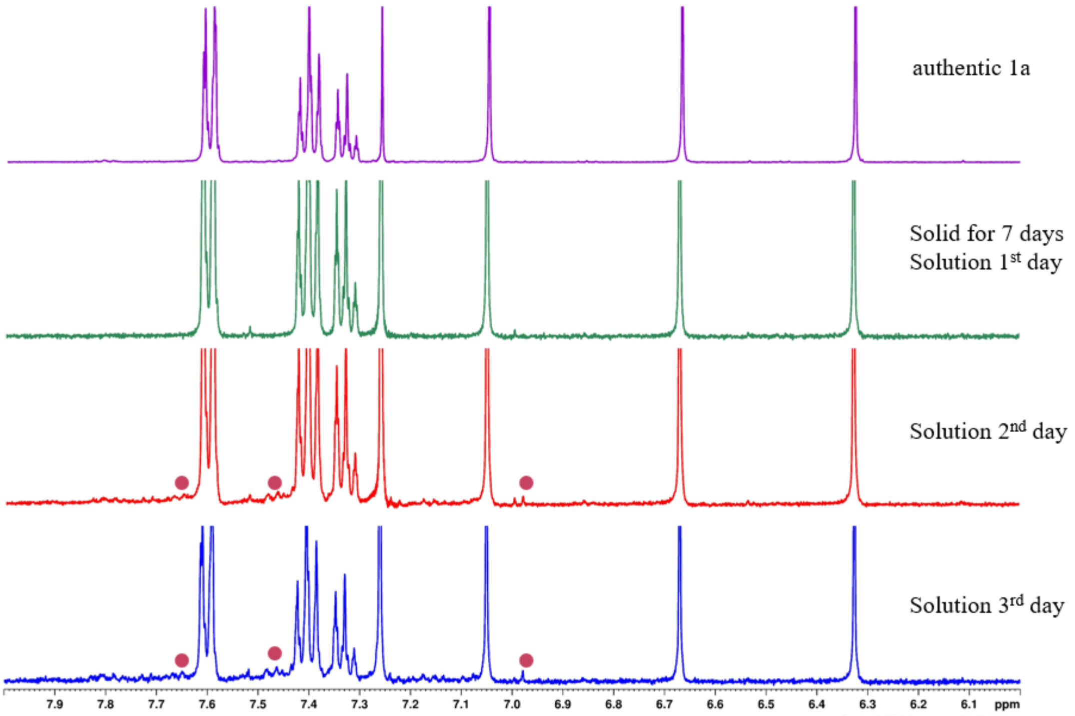

Figure S2. Magnified aromatic region of ^{1}H NMR spectra of monitoring the stability of **1a** (400 MHz, $CDCl_3$, 298 K). (red spots: decomposition peaks)

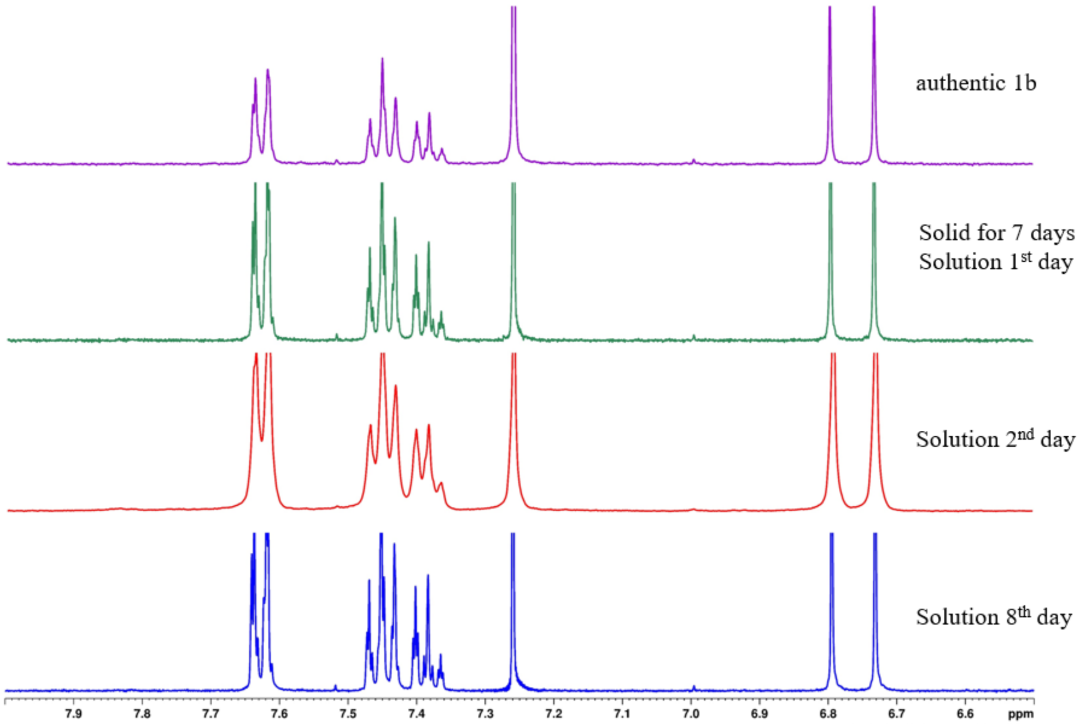

Figure S3. Magnified aromatic region of ^{1}H NMR spectra of monitoring the stability of **1b** (400 MHz, $CDCl_3$, 298 K).

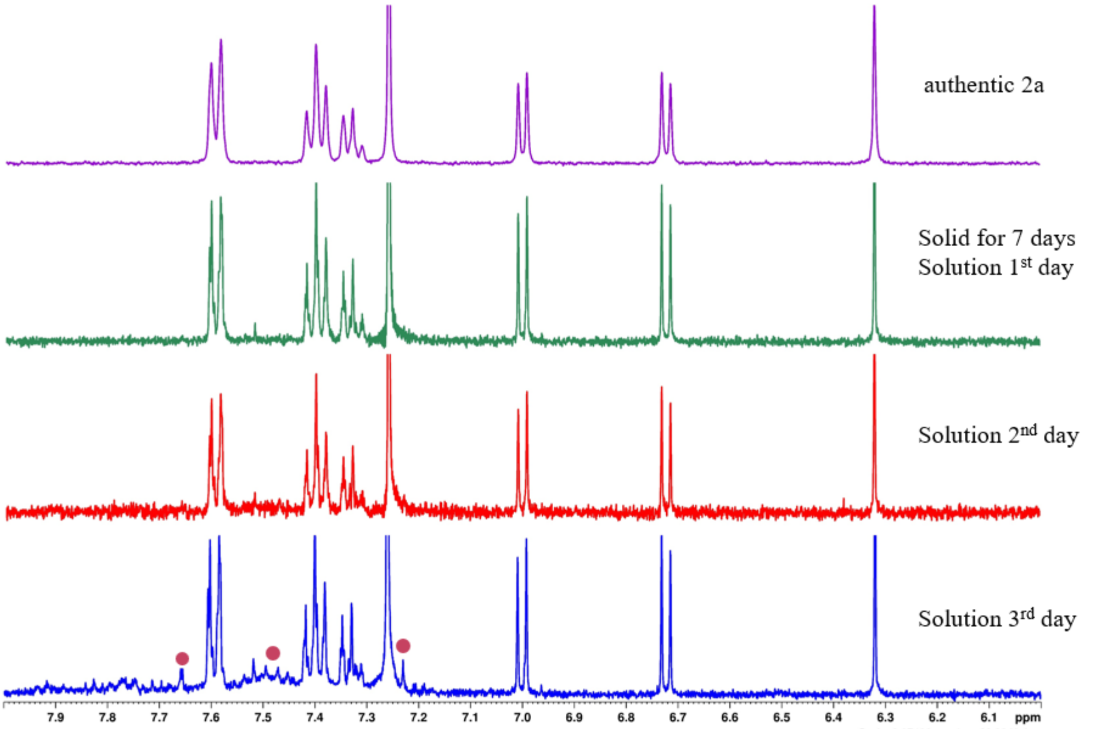

Figure S4. Magnified aromatic region of ^{1}H NMR spectra of monitoring the stability of **2a** (400 MHz, $CDCl_3$, 298 K). (red spots: decomposition peaks)

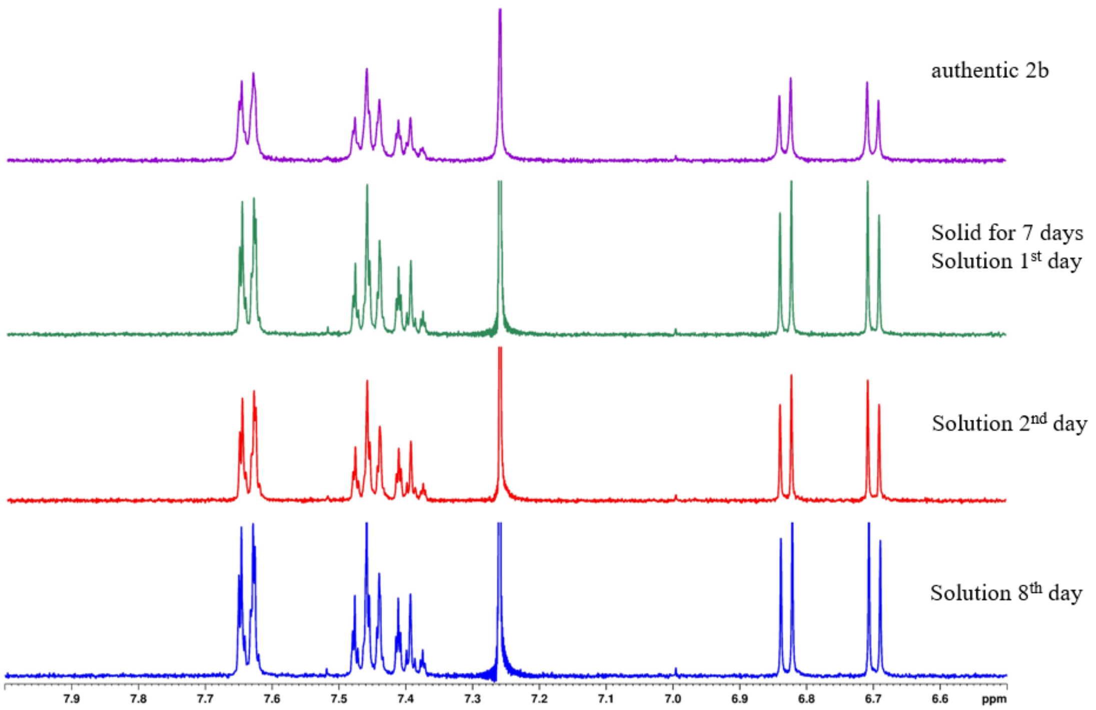

Figure S5. Magnified aromatic region of ^{1}H NMR spectra of monitoring the stability of **2b** (400 MHz, $CDCl_3$, 298 K).

3. NMR Spectra

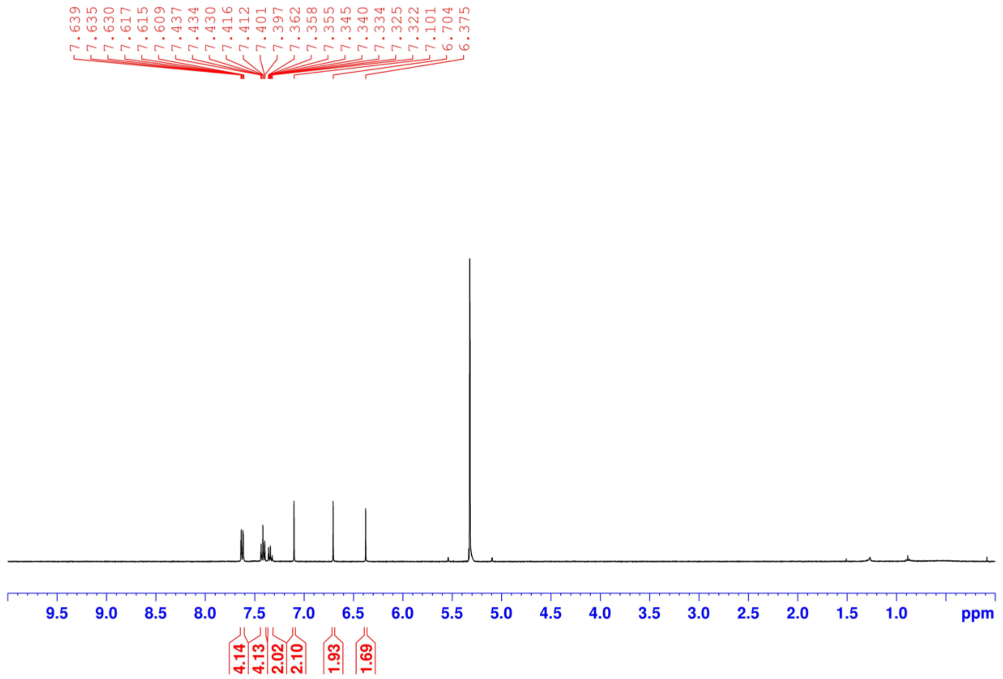

Figure S6: ^{1}H NMR Spectrum of compound **1a** (400 MHz, CD_2Cl_2, 298 K).

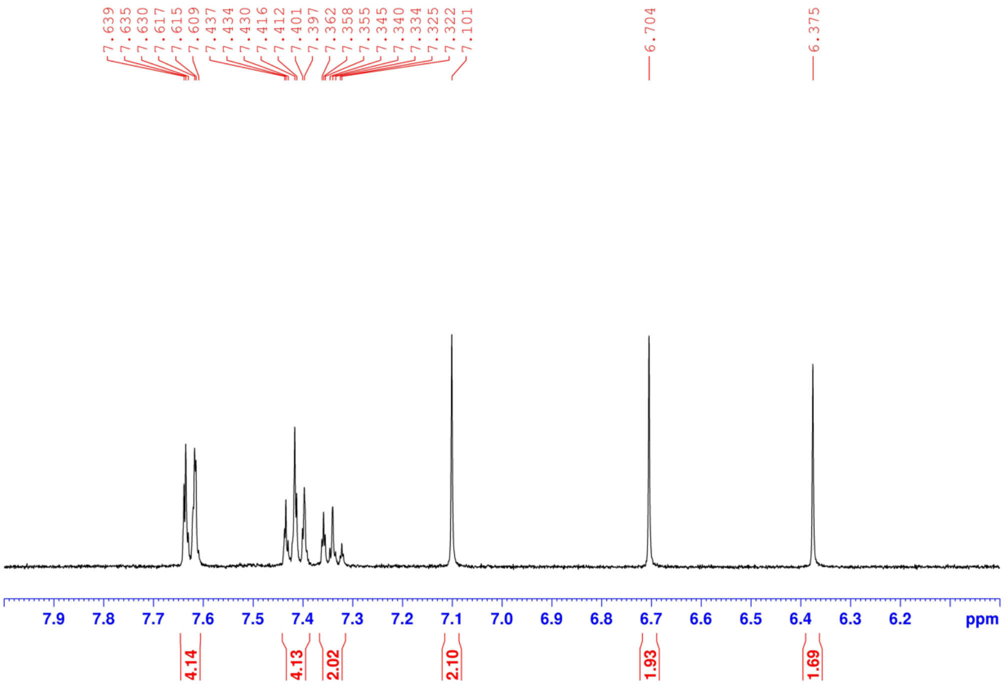

Figure S7: Magnified aromatic region of the ^{1}H NMR spectrum of compound **1a** (400 MHz, CD_2Cl_2, 298 K).

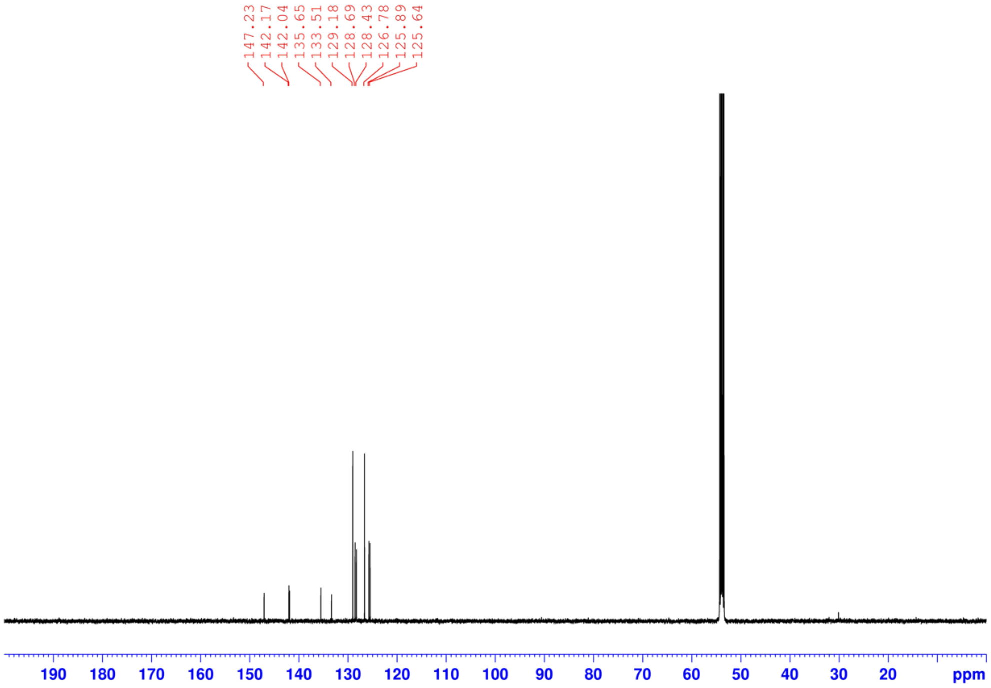

Figure S8: ^{13}C NMR spectrum of compound **1a** (125 MHz, CD_2Cl_2, 298 K).

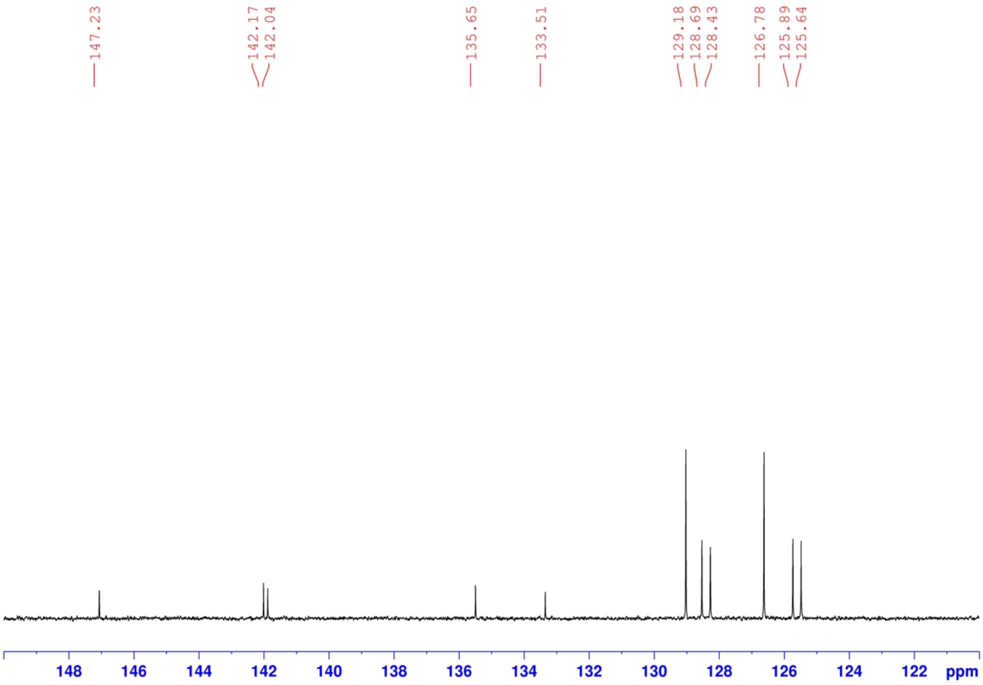

Figure S9: Magnified aromatic region of the ^{13}C NMR spectrum of compound **1a** (125 MHz, CD_2Cl_2, 298 K).

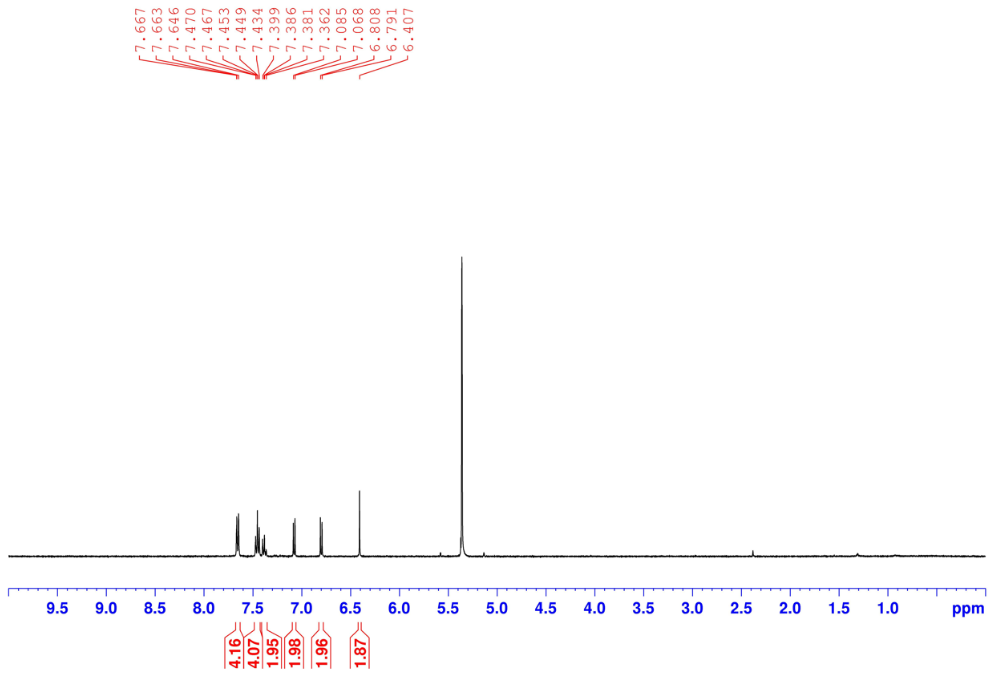

Figure S10: ^{1}H NMR Spectrum of compound **2a** (400 MHz, CD_2Cl_2, 298 K).

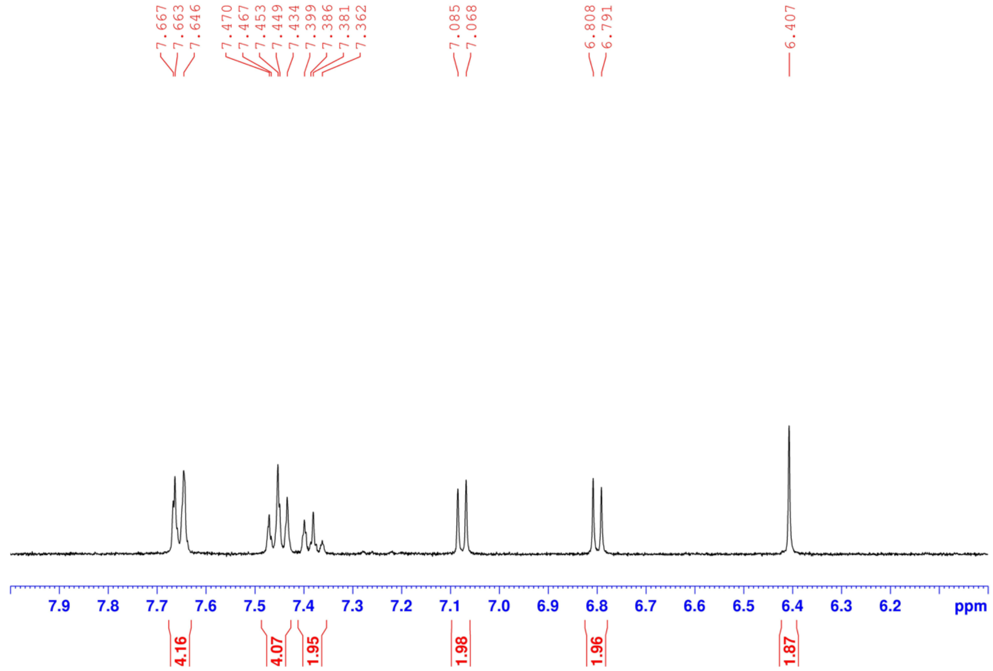

Figure S11: Magnified aromatic region of the ^{1}H NMR spectrum of compound **2a** (400 MHz, CD_2Cl_2, 298 K).

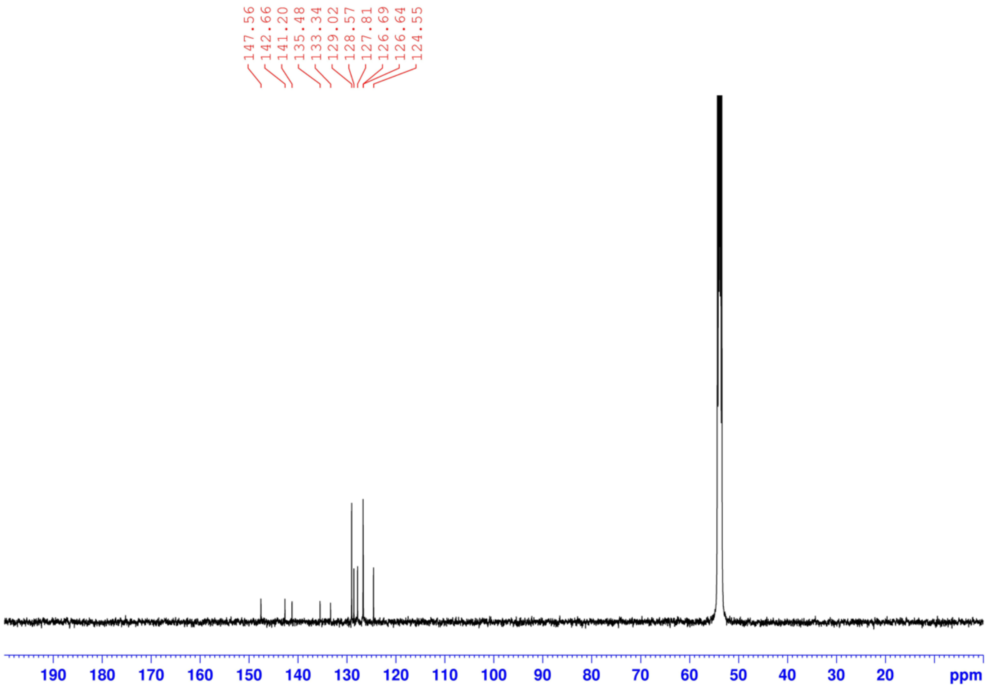

Figure S12: ^{13}C NMR spectrum of compound **2a** (125 MHz, CD_2Cl_2, 298 K).

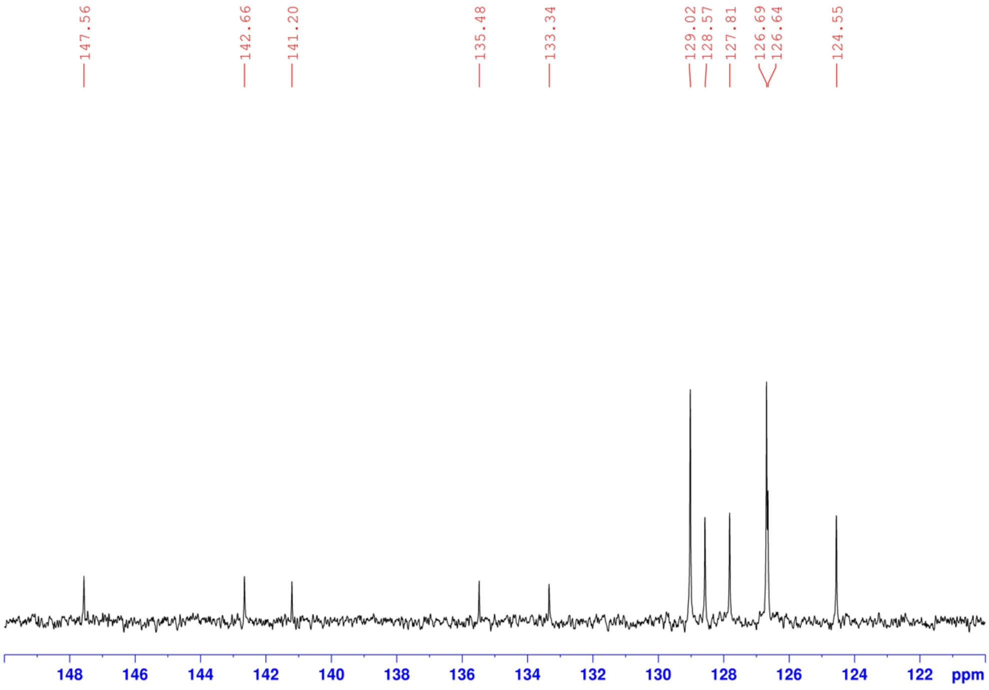

Figure S13: Magnified aromatic region of the ^{13}C NMR spectrum of compound **2a** (125 MHz, CD_2Cl_2, 298 K).

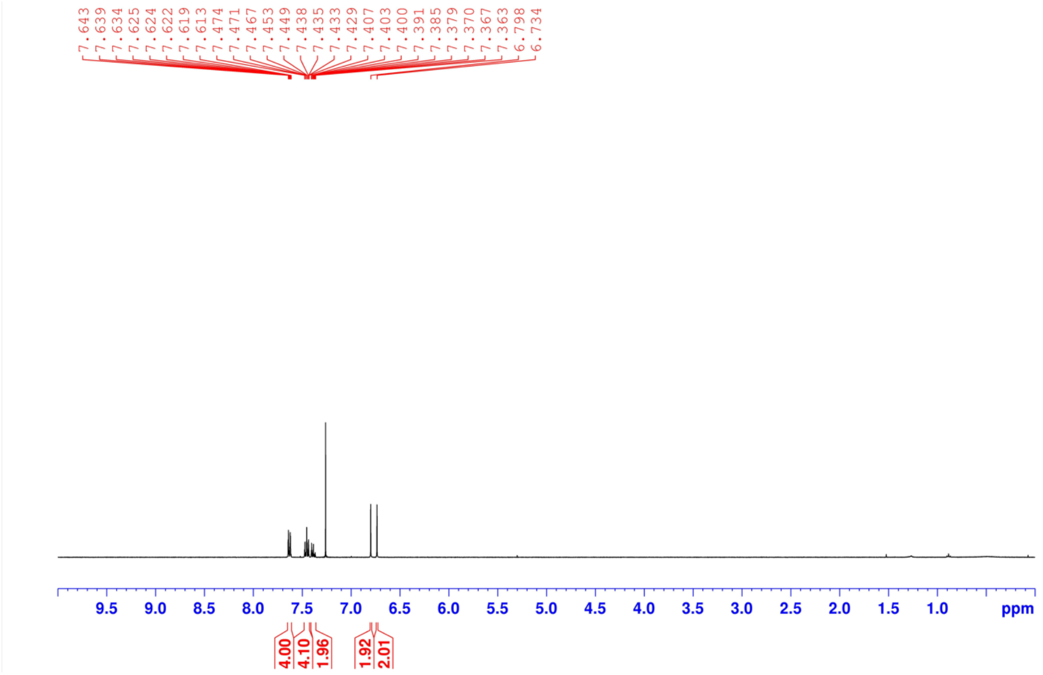

Figure S14: ^{1}H NMR Spectrum of compound **1b** (400 MHz, $CDCl_3$, 298 K).

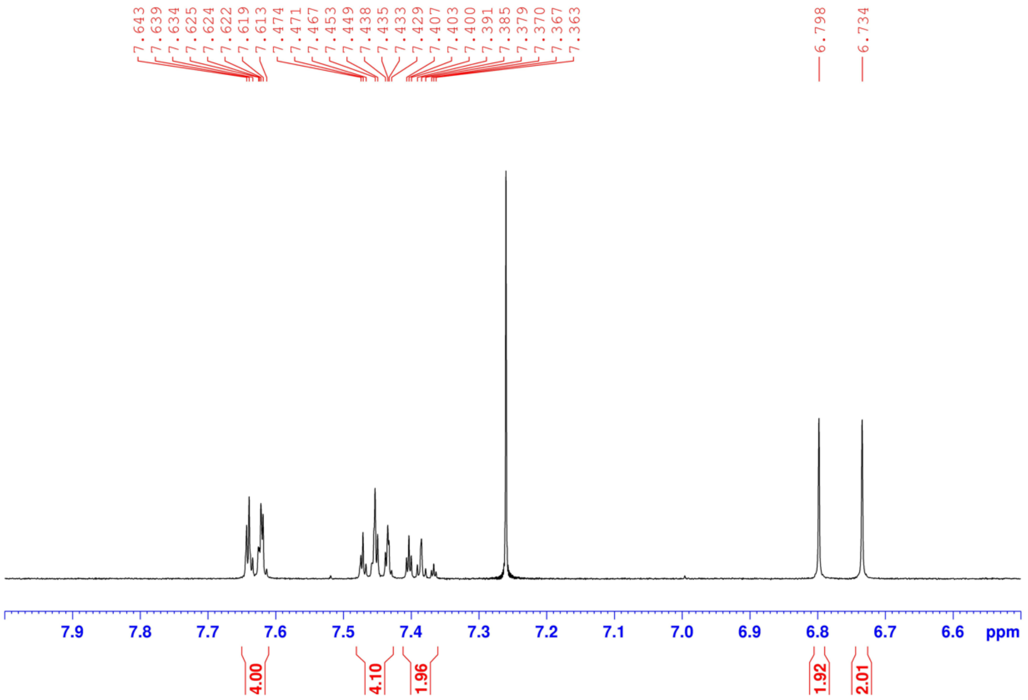

Figure S15: Magnified aromatic region of the ^{1}H NMR spectrum of compound **1b** (400 MHz, $CDCl_3$, 298 K).

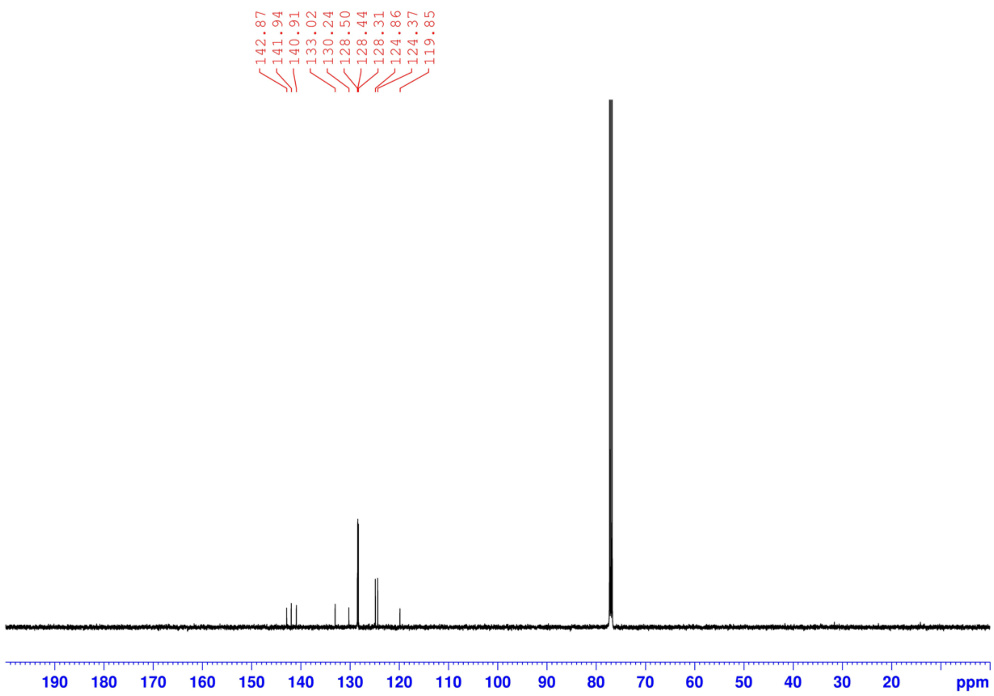

Figure S16: ^{13}C NMR spectrum of compound **1b** (125 MHz, $CDCl_3$, 298 K).

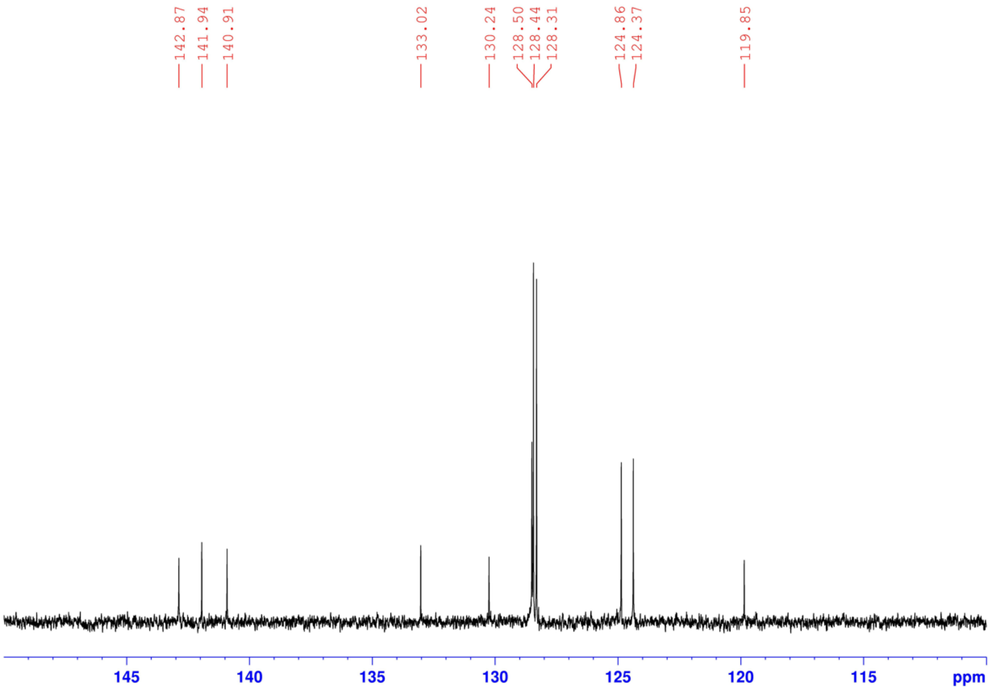

Figure S17: Magnified aromatic region of the ^{13}C NMR spectrum of compound **1b** (125 MHz, $CDCl_3$, 298 K).

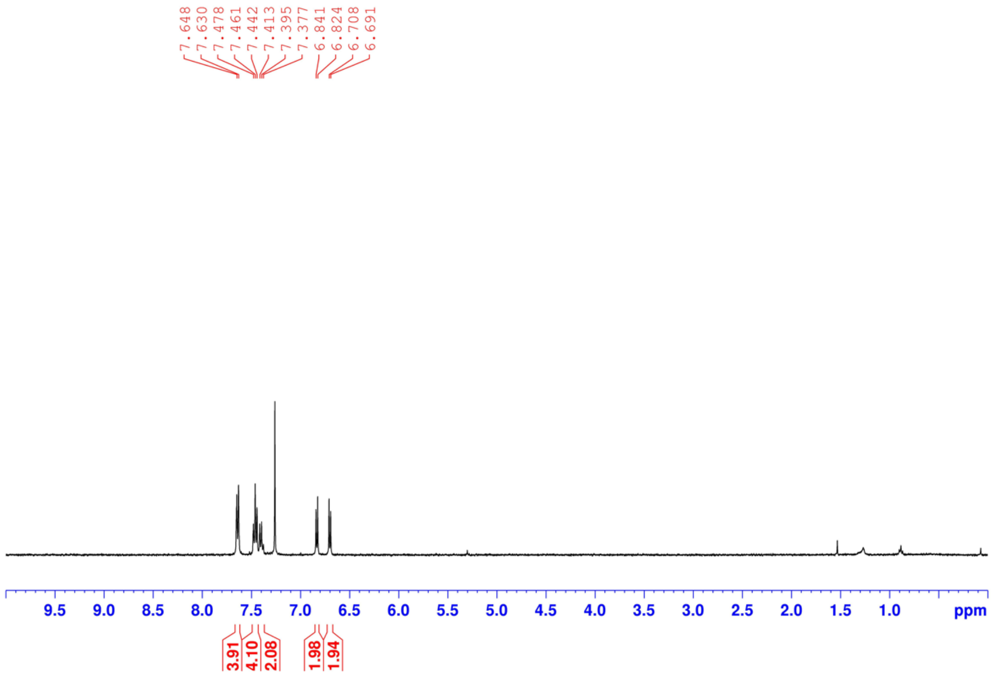

Figure S18: ^{1}H NMR Spectrum of compound **2b** (400 MHz, $CDCl_3$, 298 K).

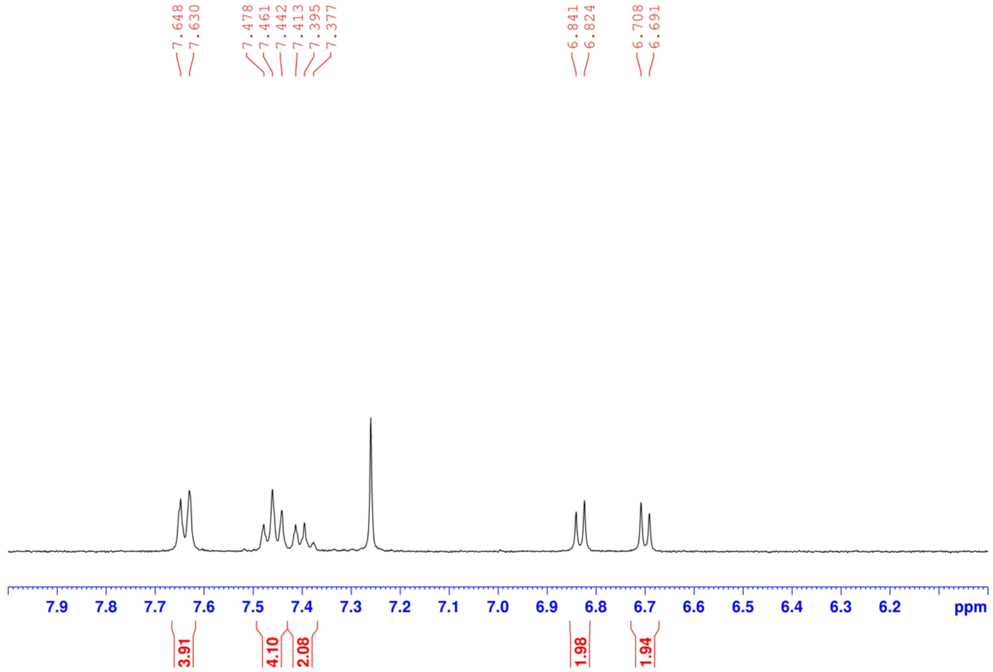

Figure S19: Magnified aromatic region of the ^{1}H NMR spectrum of compound **2b** (400 MHz, $CDCl_3$, 298 K).

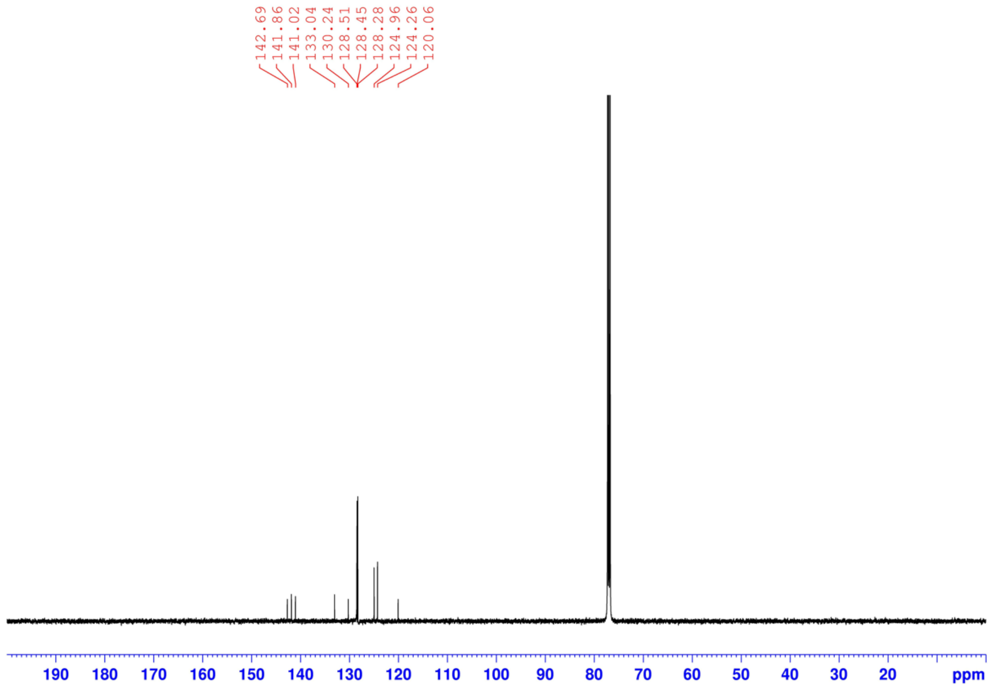

Figure S20: ^{13}C NMR spectrum of compound **2b** (125 MHz, $CDCl_3$, 298 K).

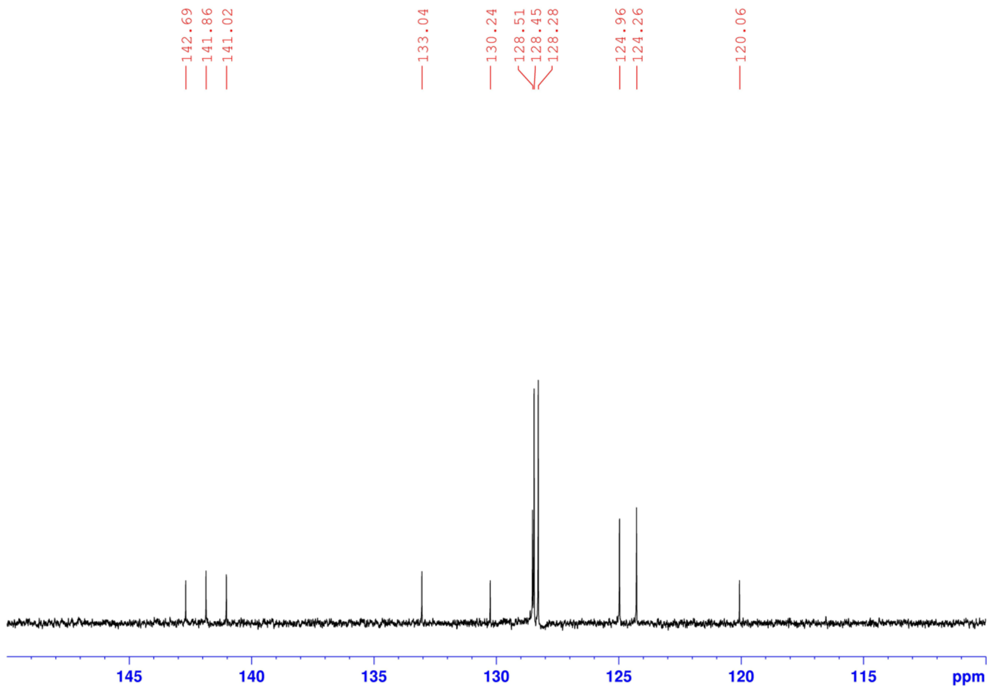

Figure S21: Magnified aromatic region of the ^{13}C NMR spectrum of compound **2b** (125 MHz, $CDCl_3$, 298 K).

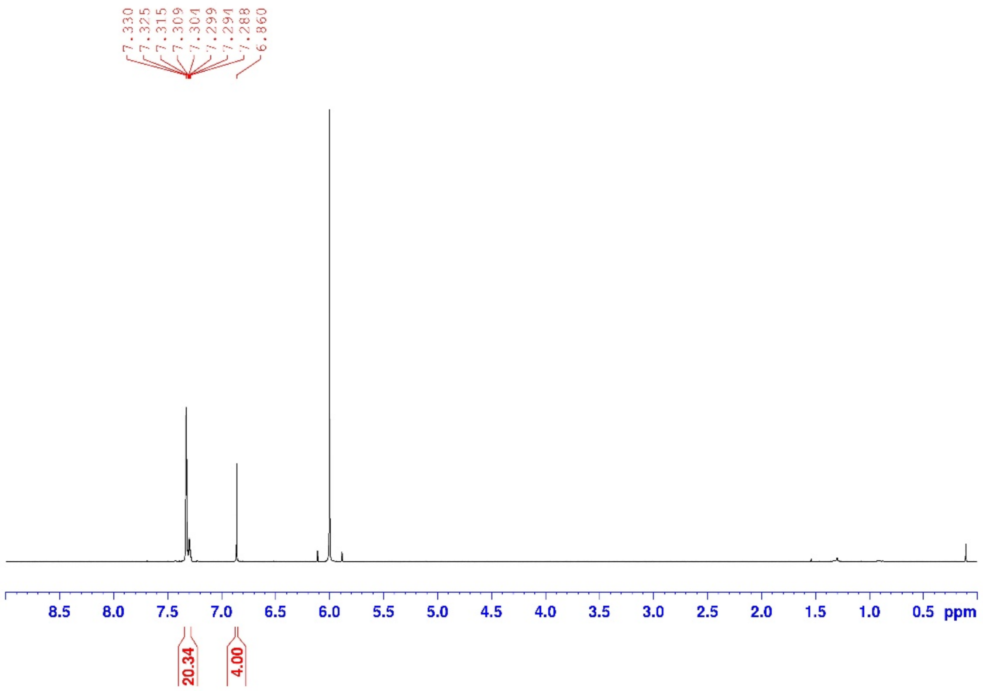

Figure S22: ^{1}H NMR Spectrum of compound **3** (800 MHz, TCE-d_2, 333 K).

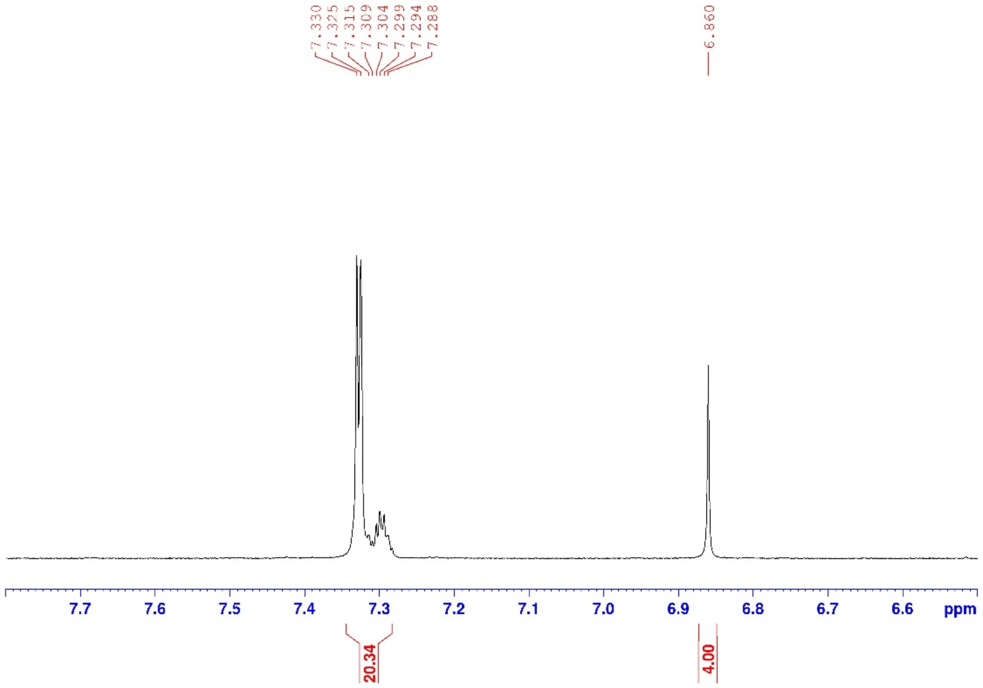

Figure S23: Magnified aromatic region of the ^{1}H NMR spectrum of compound **3** (800 MHz, TCE-d2, 333 K).

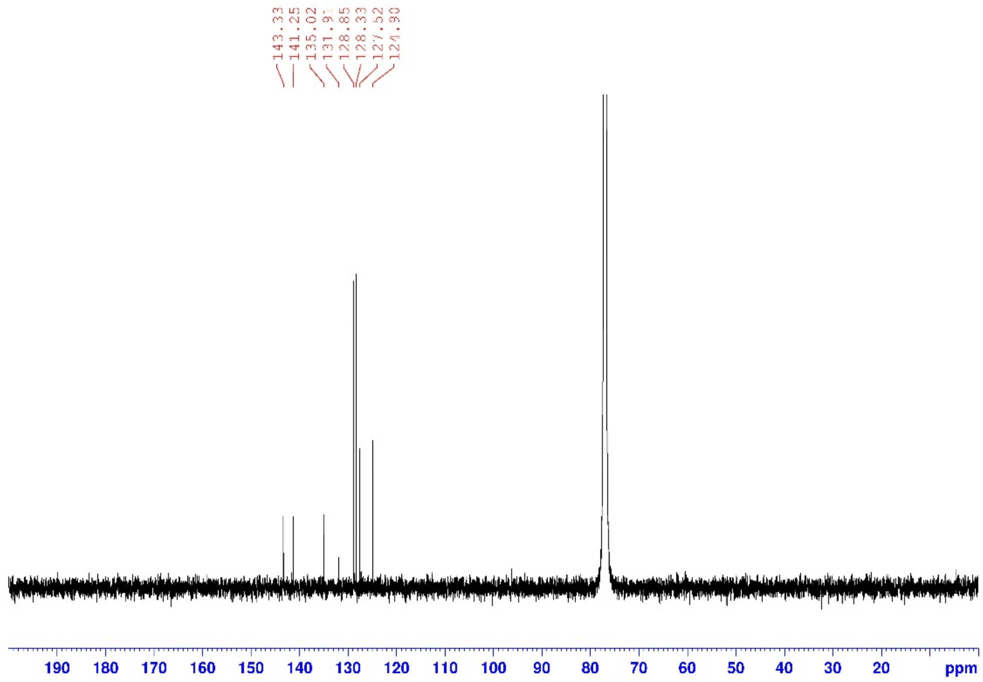

Figure S24: ^{13}C NMR spectrum of compound **3** (800 MHz, $CDCl_3$, 298 K).

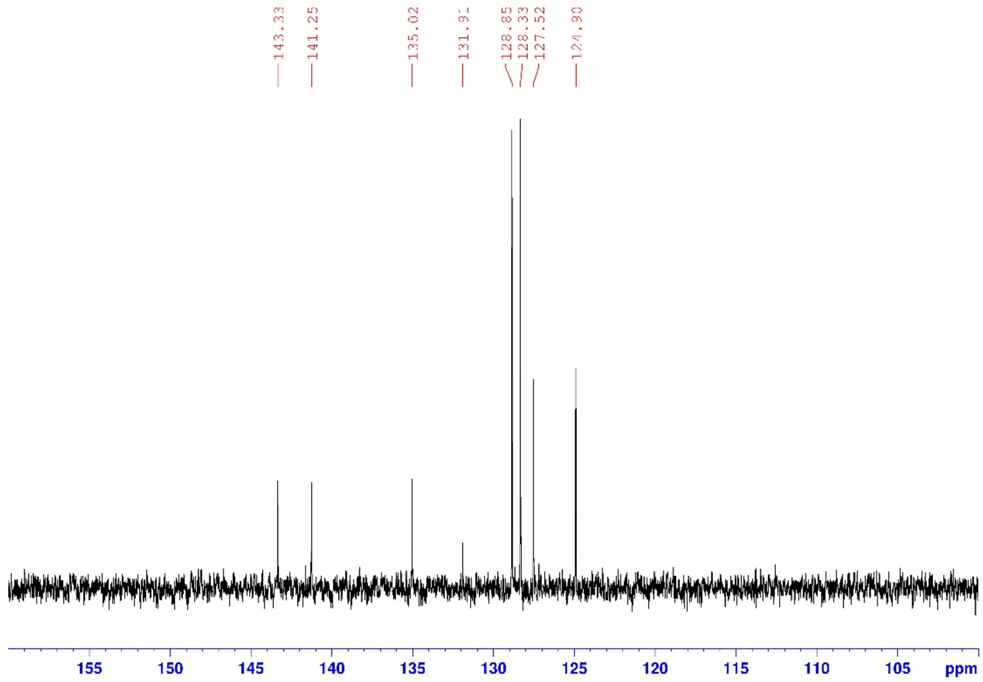

Figure S25: Magnified aromatic region of the ^{13}C NMR spectrum of compound **3** (800 MHz, $CDCl_3$, 298 K).

4. Simulated and measured HR-MS spectra

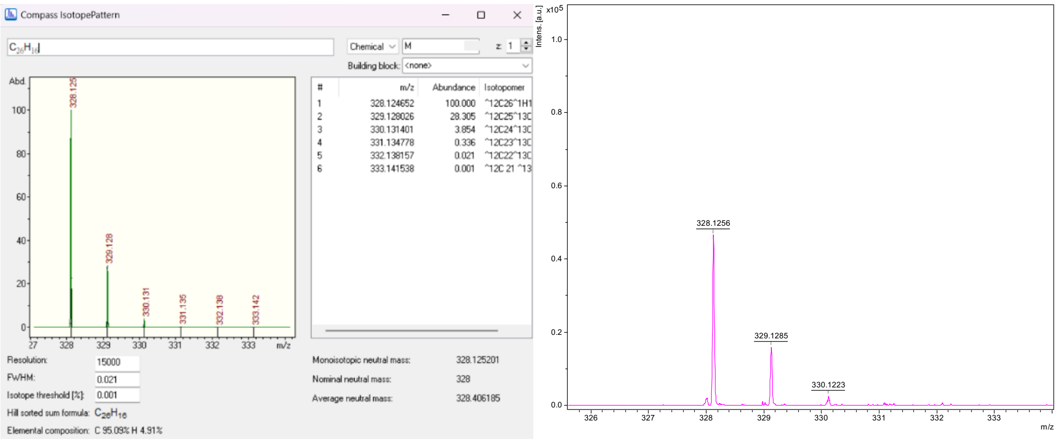

Figure S26. Simulated (left) and found (right) patterns of the high-resolution mass spectrum of **1a** (MALDI-TOF, positive mode) *m/z*: $[M]^+$ Calc'd for $C_{26}H_{16}$.

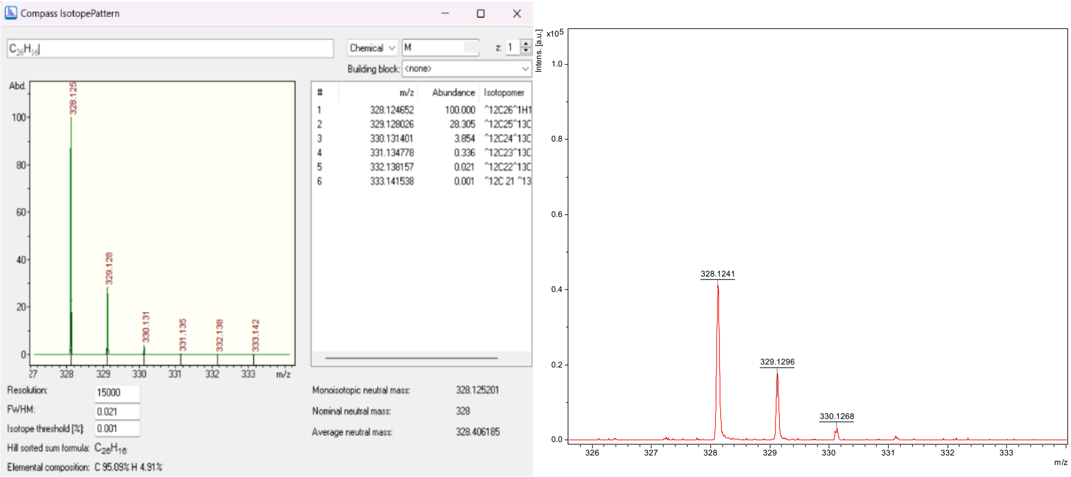

Figure S27. Simulated (left) and found (right) patterns of **2a** (MALDI-TOF, positive mode) *m/z*: $[M]^+$ Calc'd for $C_{26}H_{16}$.

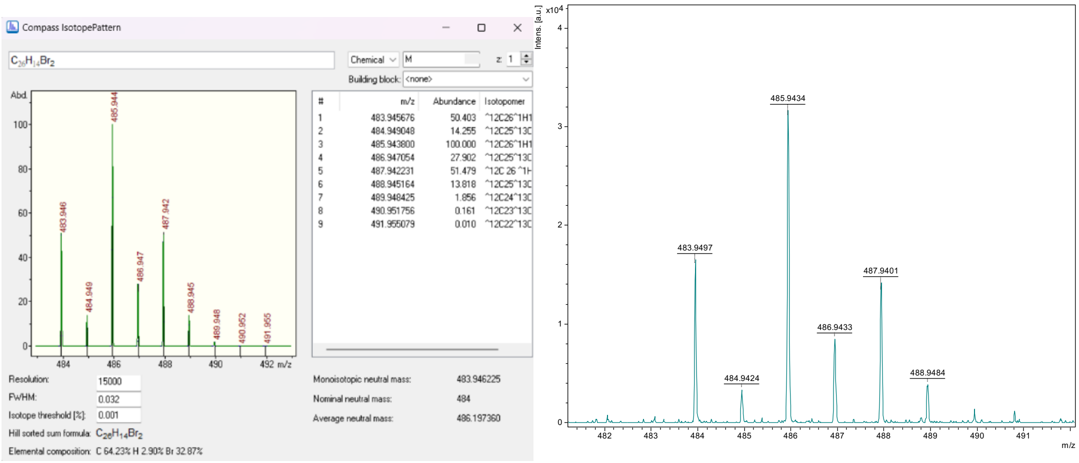

Figure S28. Simulated (left) and found (right) patterns of **1b** (MALDI-TOF, positive mode) *m/z*: $[M]^+$ Calc'd for $C_{26}H_{14}Br_2$.

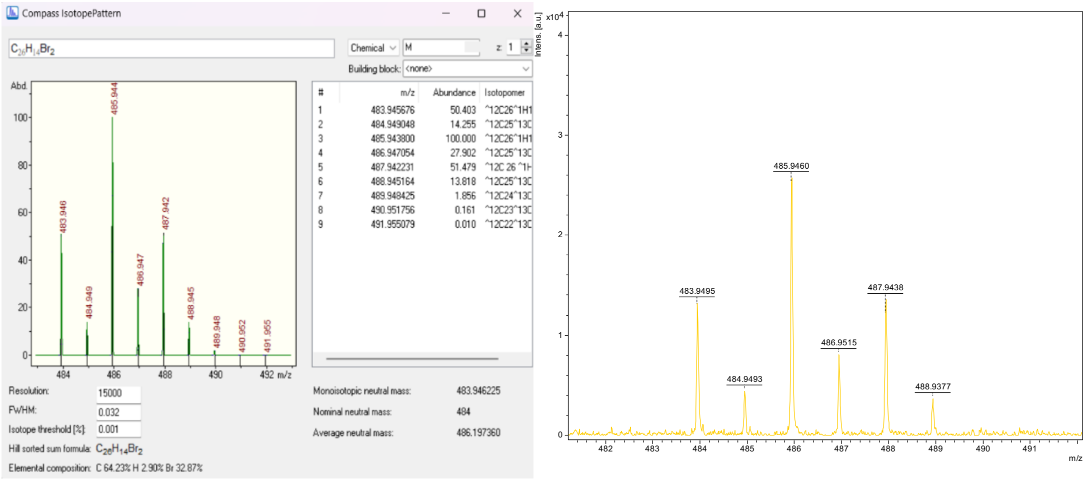

Figure S29. Simulated (left) and found (right) patterns of **2b** (MALDI-TOF, positive mode) *m/z*: $[M]^+$ Calc'd for $C_{26}H_{14}Br_2$.

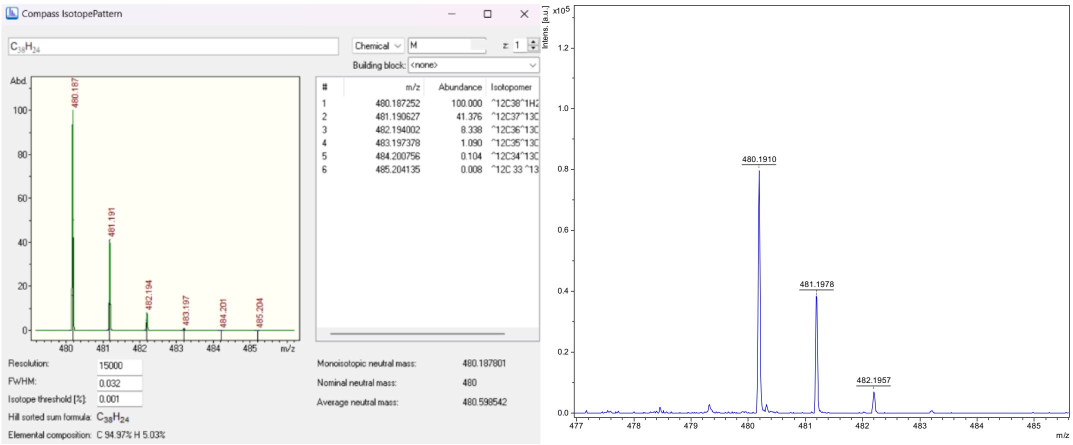

Figure S30. Simulated (left) and found (right) patterns of **3** (MALDI-TOF, positive mode) *m/z*: $[M]^+$ Calc'd for $C_{38}H_{24}$.

5. UV–Vis Spectroscopy

a)

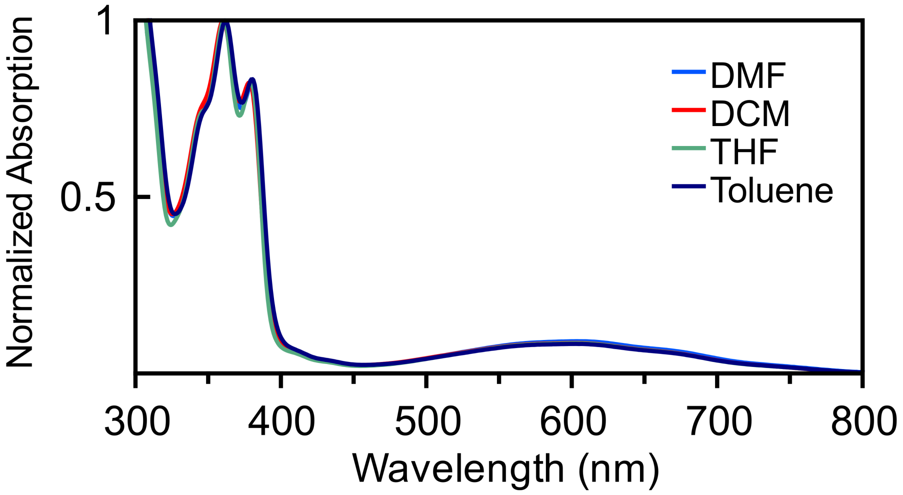

b)

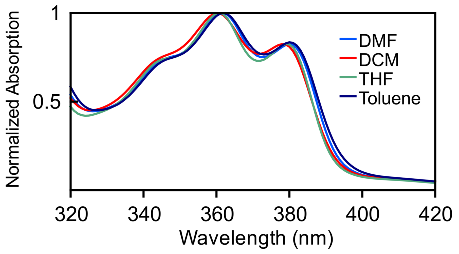

c)

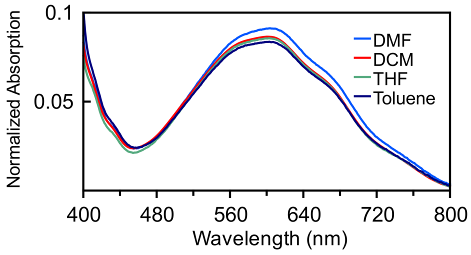

Figure S31. a) UV-Vis absorption spectra of **1a** (10^{-5} M in various organic solvents, 298 K). b) Magnified 320-420 nm regions of UV-Vis absorption spectra of **1a** (10^{-5} M in various organic solvents, 298 K). c) Magnified 400-800 nm regions of UV-Vis absorption spectra of **1a** (10^{-5} M in various organic solvents, 298 K)

a)

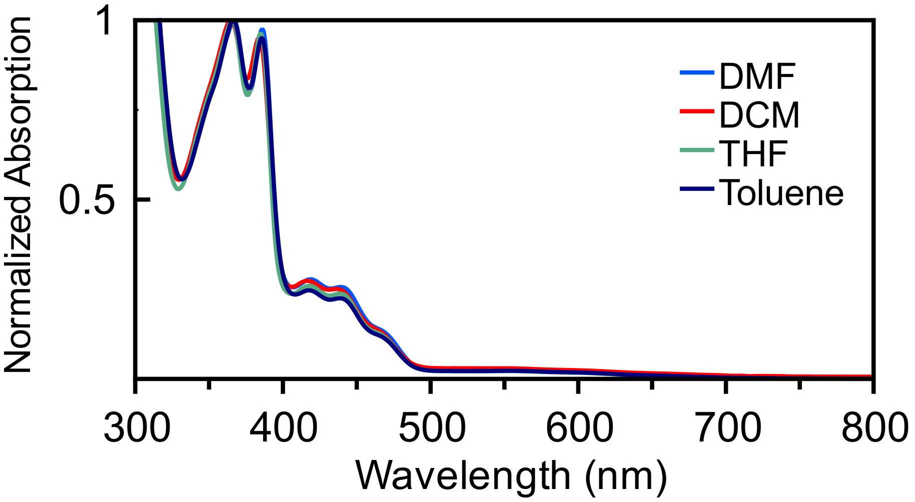

b)

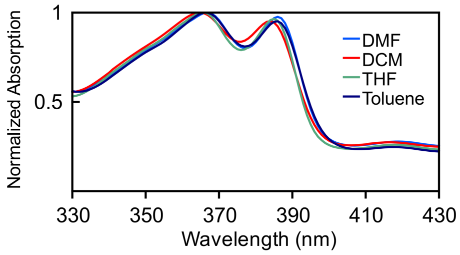

c)

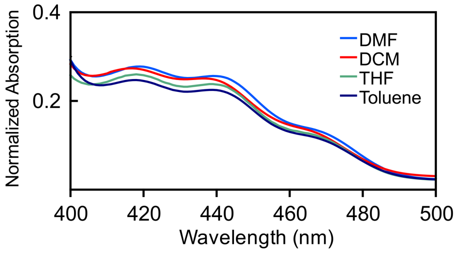

Figure S32. a) UV-Vis absorption spectra of **2a** (10^{-5} M in various organic solvents, 298 K). b) Magnified 330-430 nm regions of UV-Vis absorption spectra of **2a** (10^{-5} M in various organic solvents, 298 K). c) Magnified 400-500 nm regions of UV-Vis absorption spectra of **2a** (10^{-5} M in various organic solvents, 298 K).

a)

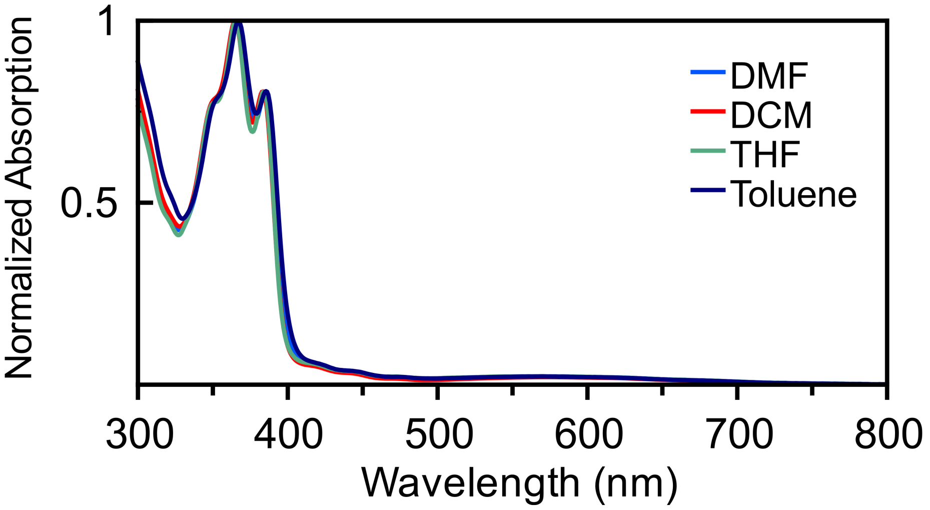

b)

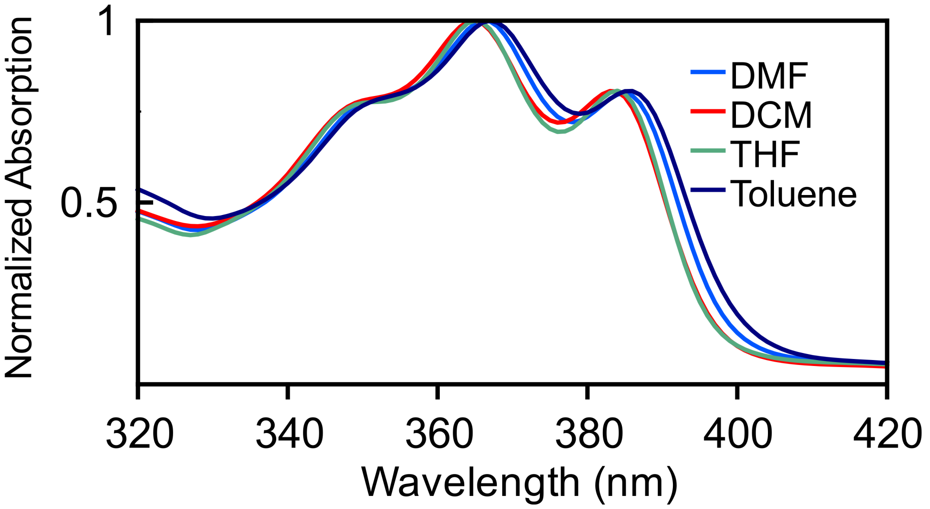

Figure S33. a) UV-Vis absorption spectra of **1b** (10^{-5} M in various organic solvents, 298 K). b) Magnified 320-420 nm regions of UV-Vis absorption spectra of **1b** (10^{-5} M in various organic solvents, 298 K).

a)

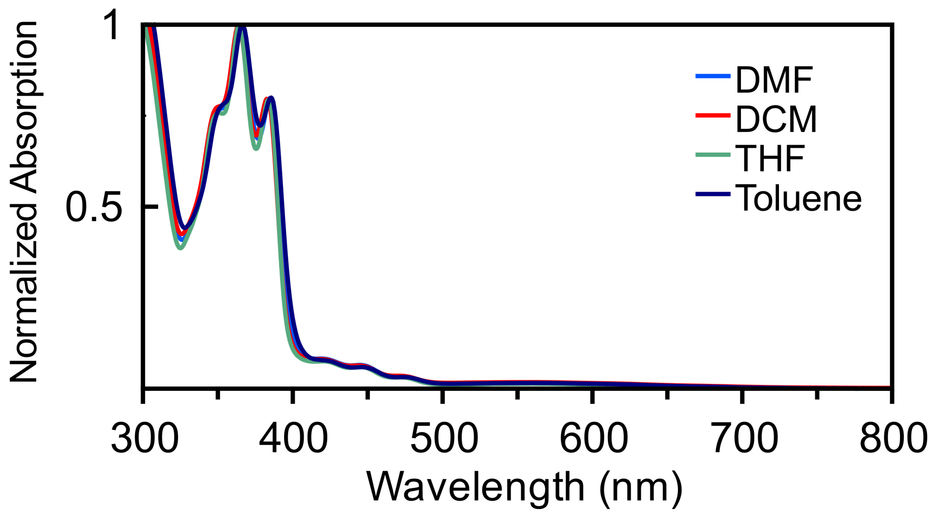

b)

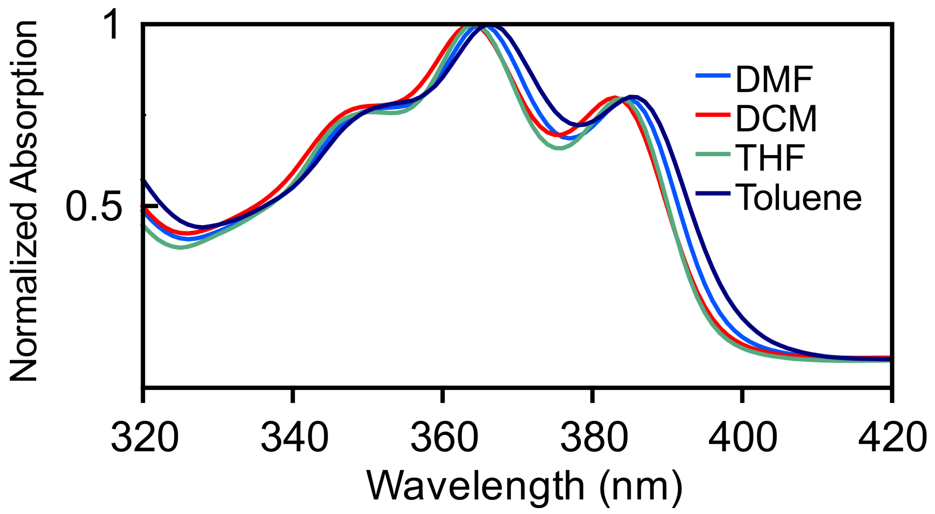

Figure S34. a) UV-Vis absorption spectra of **2b** (10^{-5} M in various organic solvents, 298 K). b) Magnified 320-420 nm regions of UV-Vis absorption spectra of **2b** (10^{-5} M in various organic solvents, 298 K).

a)

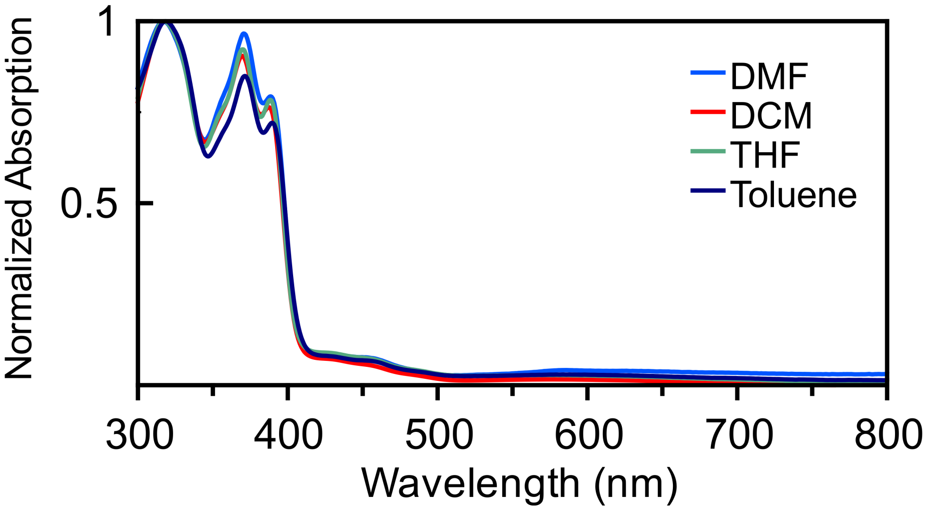

b)

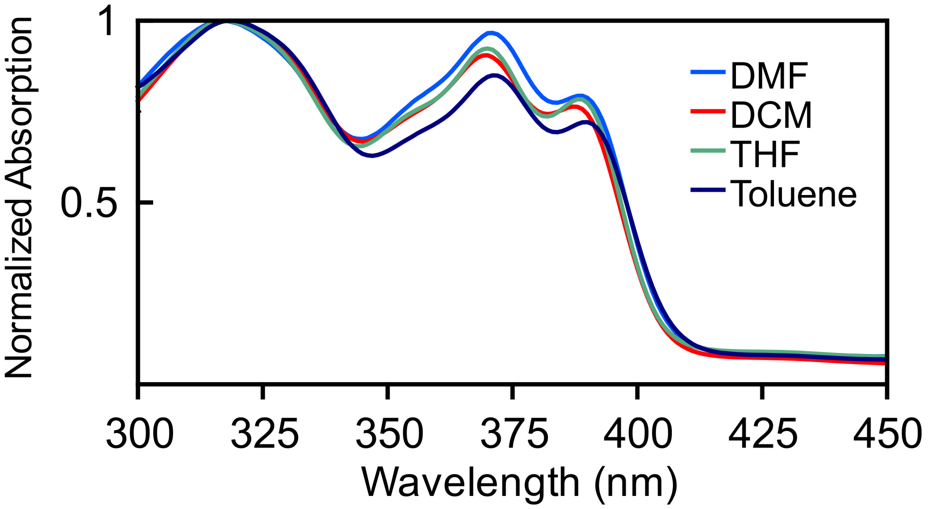

Figure S35. a) UV-Vis absorption spectra of **3** (10^{-5} M in various organic solvents, 298 K). b) Magnified 300-450 nm regions of UV-Vis absorption spectra of **3** (10^{-5} M in various organic solvents, 298 K).

6. Cyclic Voltammetry

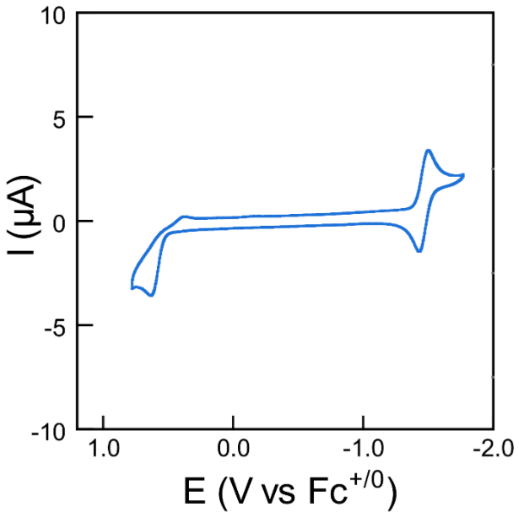

Figure S36. Full cyclic voltammogram of 1,6-diphenylpyracylene (**1a**) (1.8 x 10^{-4} M, 0.1 M n-Bu_4NPF_6, in CH_2Cl_2, vs. $Fc^{+/0}$, 298K).

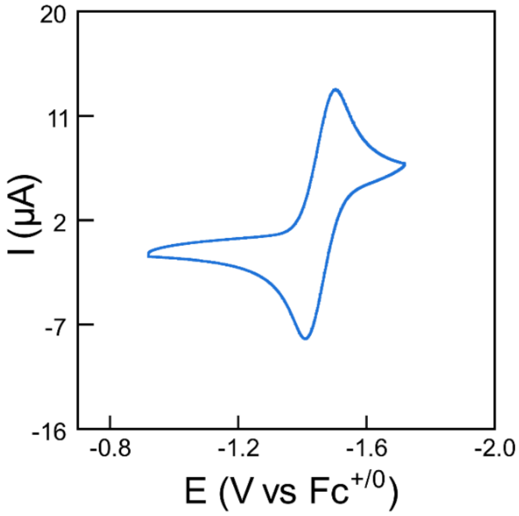

Figure S37. Cyclic voltammogram of the first reduction of 1,6-diphenylpyracylene (**1a**) (1.8 x 10^{-4} M, 0.1 M n-Bu_4NPF_6, in CH_2Cl_2, vs. $Fc^{+/0}$, 298K).

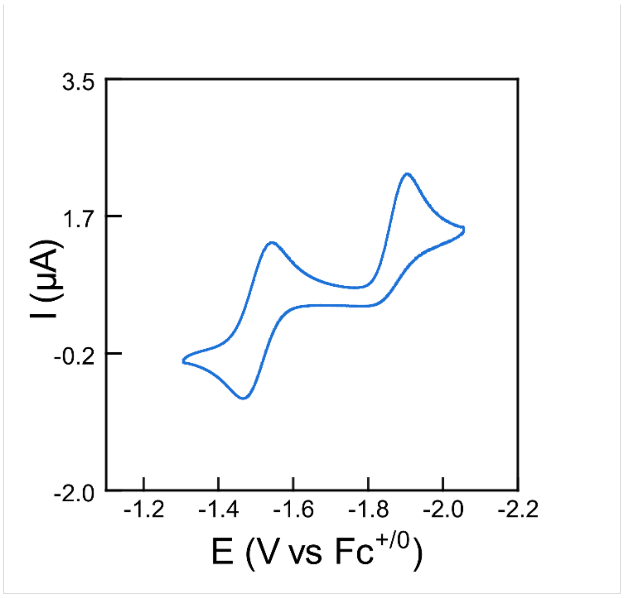

Figure S38. Cyclic voltammogram of the reduction of 1,6-diphenylpyracylene (**1a**) (2.0 x 10^{-4} M, 0.1 M n-Bu_4NPF_6, in THF, vs. $Fc^{+/0}$, 298K).

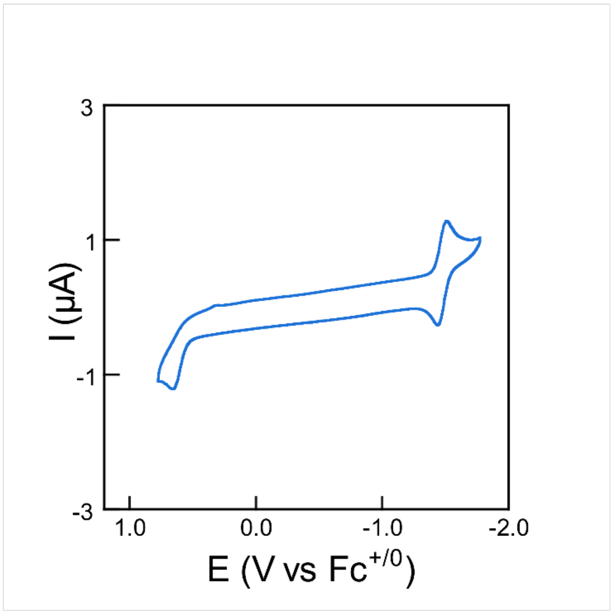

Figure S39. Full cyclic voltammogram of 1,5-diphenylpyracylene (**2a**) (2.2 x 10^{-4} M, 0.1 M n-Bu_4NPF_6, in CH_2Cl_2, vs. $Fc^{+/0}$, 298K).

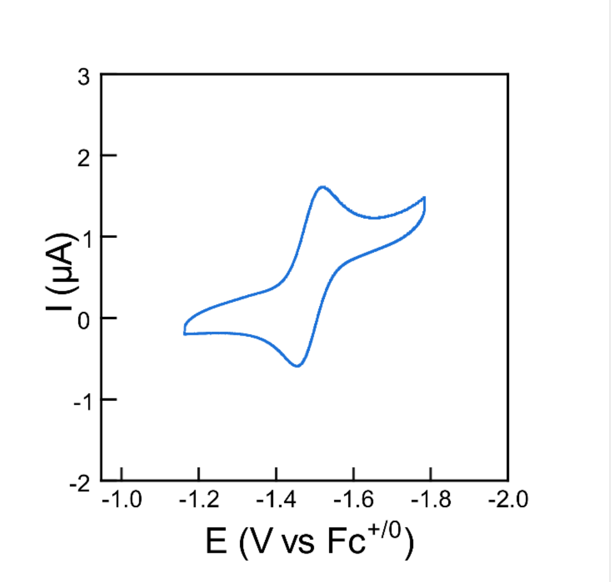

Figure S40. Cyclic voltammogram of the first reduction of 1,5-diphenylpyracylene (**2a**) (2.2 x 10^{-4} M, 0.1 M n-Bu_4NPF_6, in CH_2Cl_2, vs. $Fc^{+/0}$, 298K).

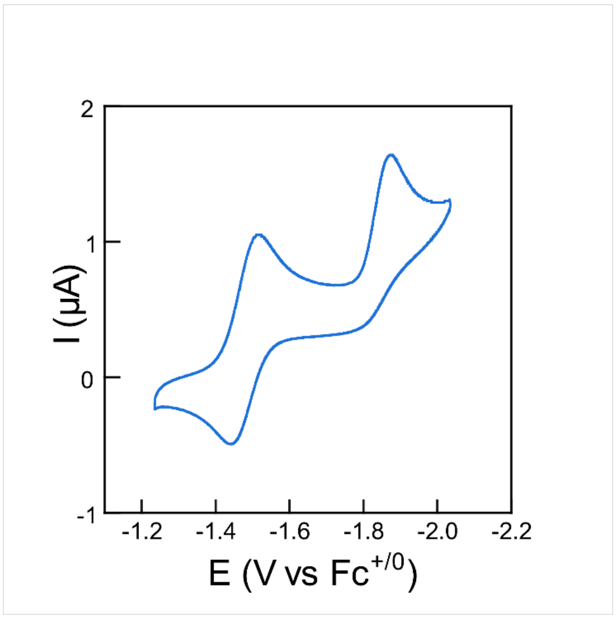

Figure S41. Cyclic voltammogram of the reduction of 1,5-diphenylpyracylene (**2a**) (1.4 x 10^{-4} M, 0.1 M n-Bu_4NPF_6, in THF, vs. $Fc^{+/0}$, 298K).

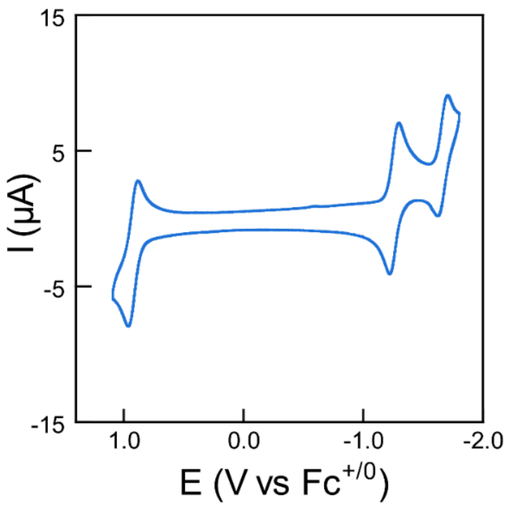

Figure S42. Full cyclic voltammogram of 1,6-dibromo-2,5-diphenylpyracylene (**1b**) (1.6 x 10^{-4} M, 0.1 M n-Bu_4NPF_6, in CH_2Cl_2, vs. $Fc^{+/0}$, 298K).

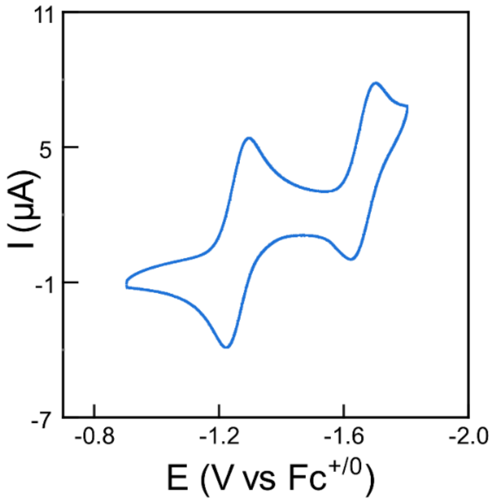

Figure S43. Cyclic voltammogram of the reduction of 1,6-dibromo-2,5-diphenylpyracylene (**1b**) (1.6 x 10^{-4} M, 0.1 M n-Bu_4NPF_6, in CH_2Cl_2, vs. $Fc^{+/0}$, 298K).

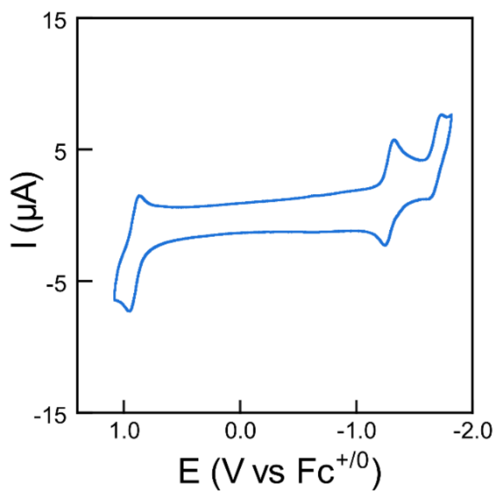

Figure S44. Full cyclic voltammogram of 1,5-dibromo-2,6-diphenylpyracylene (**2b**) (1.0 x 10^{-4} M, 0.1 M n-Bu_4NPF_6, in CH_2Cl_2, vs. $Fc^{+/0}$, 298K).

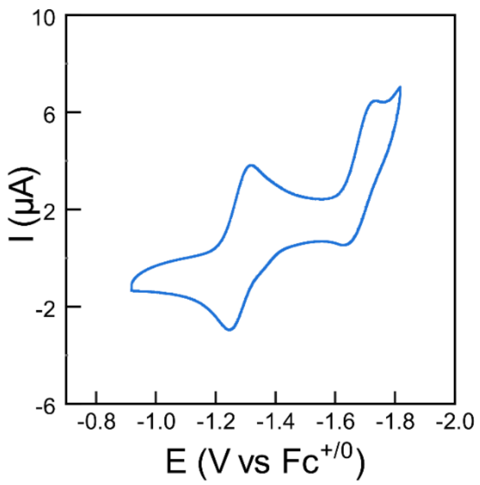

Figure S45. Cyclic voltammogram of the reduction of 1,5-dibromo-2,6-diphenylpyracylene (**2b**) (1.0 x 10^{-4} M, 0.1 M n-Bu_4NPF_6, in CH_2Cl_2, vs. $Fc^{+/0}$, 298K).

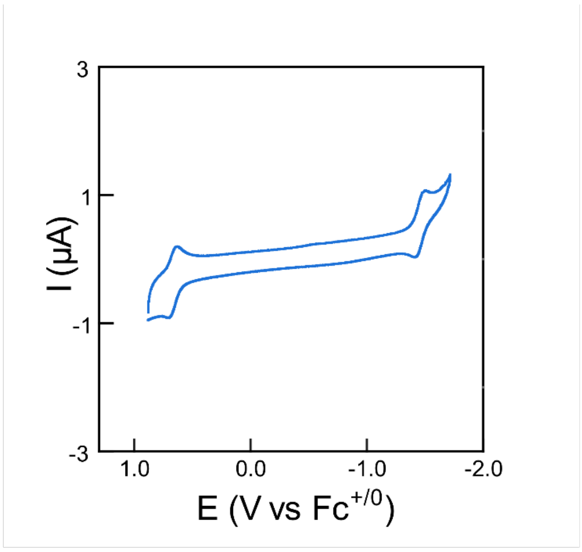

Figure S46. Full cyclic voltammogram of 1,2,5,6-tetraphenylpyracylene (**3**) (1.3 x 10^{-4} M, 0.1 M n-Bu_4NPF_6, in CH_2Cl_2, vs. $Fc^{+/0}$, 298K).

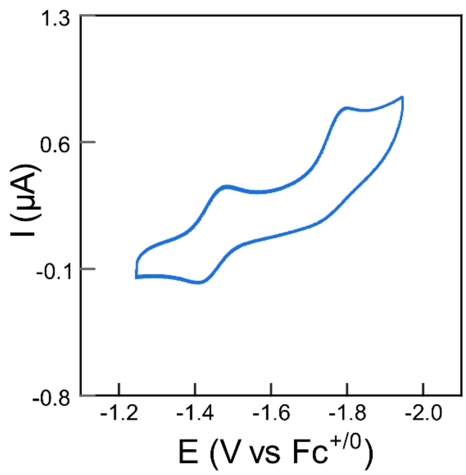

Figure S47. Cyclic voltammogram of the reduction of (**3**) (8.3 x 10^{-5} M, 0.1 M n-Bu_4NPF_6, in THF, vs. $Fc^{+/0}$, 298K).

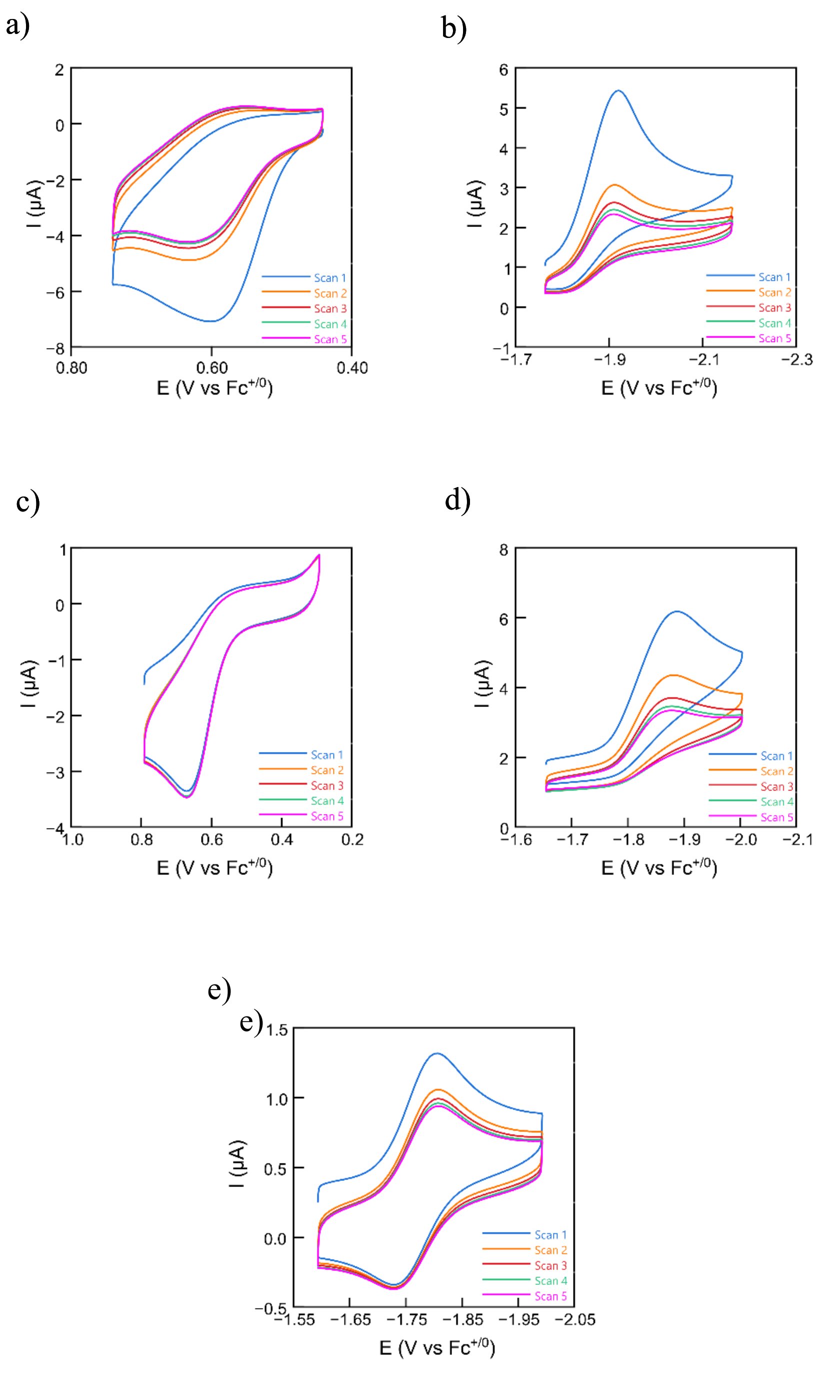

Figure S48. Cyclic voltammograms of multiple scanning cycles of the a) oxidation wave of **1a** (2.7 x 10^{-4} M, 0.1 M n-Bu_4NPF_6, in CH_2Cl_2, vs. $Fc^{+/0}$, 298K**)**, b) second reduction wave of **1a** (2.7 x 10^{-4} M, 0.1 M n-Bu_4NPF_6, in THF, vs. $Fc^{+/0}$, 298K), c) oxidation wave of **2a** (2.7 x 10^{-4} M, 0.1 M n-Bu_4NPF_6, in CH_2Cl_2, vs. $Fc^{+/0}$, 298K), d) second reduction wave of **2a** (2.3 x 10^{-4} M, 0.1 M n-Bu_4NPF_6, in THF, vs. $Fc^{+/0}$, 298K), e) second reduction wave of **3** (1.6 x 10^{-4} M, 0.1 M n-Bu_4NPF_6, in THF, vs. $Fc^{+/0}$, 298K) (blue: scan 1, orange: scan 2, red: scan 3, green: scan 4, and pink: scan 5, scanning rate: 500 mV/s).

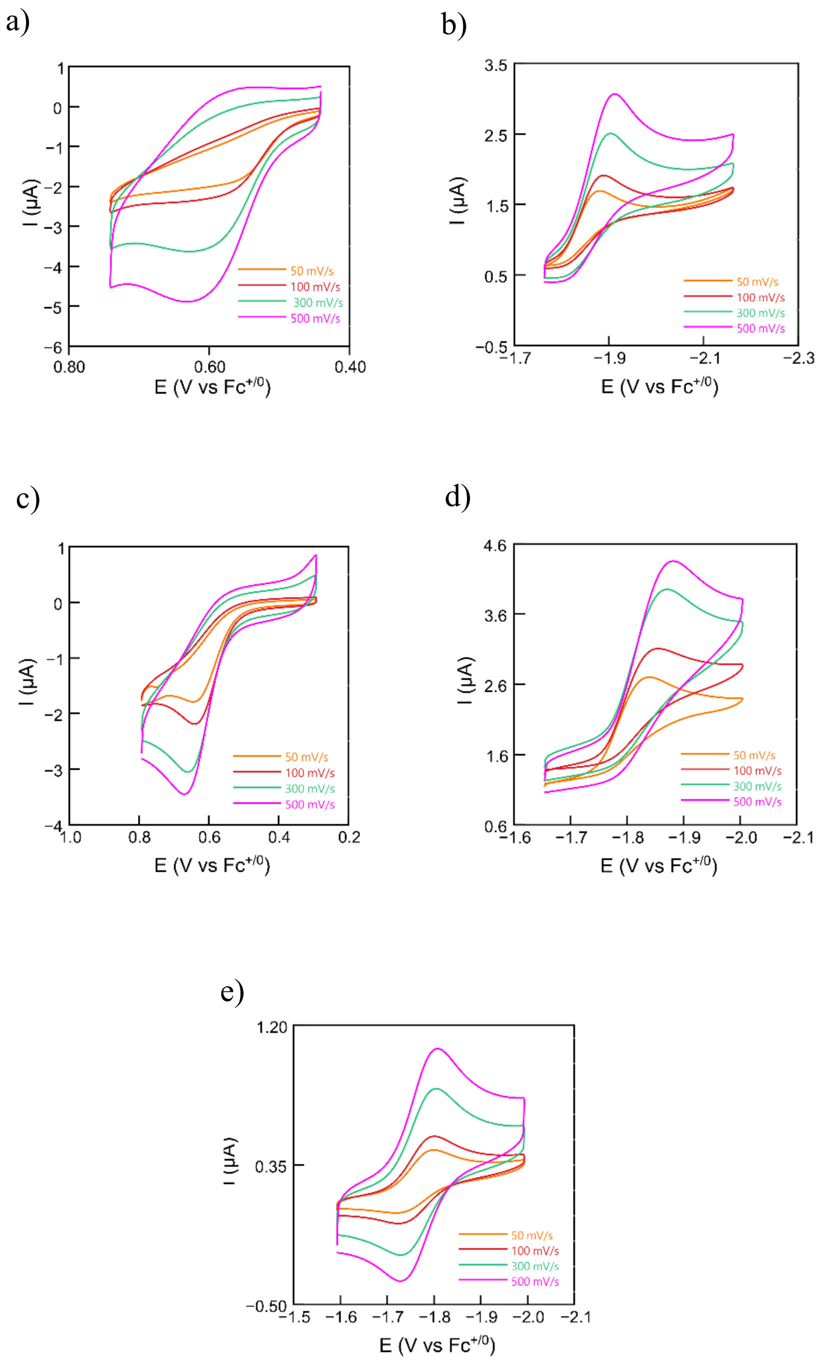

Figure S49. Cyclic voltammograms at different scanning rates of the a) oxidation wave of **1a** (2.7 x 10^{-4} M, 0.1 M n-Bu_4NPF_6, in CH_2Cl_2, vs. $Fc^{+/0}$, 298K), b) second reduction wave of **1a** (2.7 x 10^{-4} M, 0.1 M n-Bu_4NPF_6, in THF, vs. $Fc^{+/0}$, 298K), c) oxidation wave of **2a** (2.7 x 10^{-4} M, 0.1 M n-Bu_4NPF_6, in CH_2Cl_2, vs. $Fc^{+/0}$, 298K), d) second reduction wave of **2a** (2.3 x 10^{-4} M, 0.1 M n-Bu_4NPF_6, in THF, vs. $Fc^{+/0}$, 298K), e) second reduction wave of **3** (1.6 x 10^{-4} M, 0.1 M n-Bu_4NPF_6, in THF, vs. $Fc^{+/0}$, 298K). Orange: 50, red: 100, green: 300, and pink: 500 mV/s.

7. X-ray Crystallography

Table S2. Selected X-ray Crystallographic Data for **1a** and **2a.**

**Compound**	**1,6-diphenylpyracylene**	**1,5-diphenylpyracylene**
Formula weight, gmol^{-1}	328.39	328.39
T (K) / *l* (Å)	100(2)/1.54178	100(2)/1.54178
Crystal system	Orthorhombic	Orthorhombic
Space group / *Z*	$P2_12_12_1$/**4**	Pbca/4
a, Å	5.6272(3)	7.4544(2)
b, Å	10.8161(5)	7.7891(2)
c, Å	27.3457(12)	27.9214(8)
α, °	90	90
β, °	90	90
γ, °	90	90
V, Å^3	1664.38(14)	1621.20(8)
ρ, mg m^{-3}	1.311	1.345
Abs. coeff., mm^{-1}	0.564	0.579
F(000)	688	688
Crystal size, mm^3	0.100x0.050x0.025	0.100 x 0.050 x 0.050
θ range, °	3.232 to 70.100	3.165 to 70.202
Reflns collected	3704	17426
Ind. reflns	3704	1537
R(int)	N/A	0.0516
Obs. reflns [$I > 2\sigma(I)$]	3572	1455
Completeness to 2*θ*	100.0	100.0
Goodness-of-fit F^2	1.068	1.019
Final *R* [$I > 2\sigma(I)$]	R1 = 0.0458<br>wR2 = 0.1114	R1 = 0.0482<br>wR2 = 0.1340
R (all data)	R1 = 0.0478<br>wR2 = 0.1146	R1 = 0.0500<br>wR2 = 0.1362

Table S3. Selected X-ray Crystallographic Data for **1b** and **2b.**

**Compound**	**1,6-dibromo-2,5-diphenylpyracylene**	**1,5-dibromo-2,6-diphenylpyracylene**
Formula weight, $gmol^{-1}$	528.65	484.18
T (K) / l (Å)	100(2)/1.54178	100(2)/1.54178
Crystal system	Tetragonal	Orthorhombic
Space group / Z	P-421c/8	Pccn
a, Å	24.8204(6)	15.5489(9)
b, Å	24.8204(6)	17.3690(10)
c, Å	6.8571(3)	6.9097(4)
α, °	90	90
β, °	90	90
γ, °	90	90
V, $Å^3$	4224.3(3)	1866.09(19)
ρ, mg m^{-3}	1.662	1.723
Abs. coeff., mm^{-1}	6.104	5.561
F(000)	2088	952
Crystal size, mm^3	0.200x0.020x0.020	0.150 x 0.030 x 0.025
θ range, °	3.56 to 70.32	3.815 to 70.140
Reflns collected	28476	4943
Ind. reflns	4026	1753
R(int)	0.0808	0.1266
Obs. reflns [$I > 2\sigma(I)$]	3702	1297
Completeness to 2θ	100.0	99.1
Goodness-of-fit F^2	1.227	1.031
Final R [$I > 2\sigma(I)$]	R1 = 0.0536<br>wR2 = 0.1524	R1 = 0.0605<br>wR2 = 0.1520
R (all data)	R1 = 0.0579<br>wR2 = 0.1562	R1 = 0.0773<br>wR2 = 0.1677

Table S4. Selected X-ray Crystallographic Data for **3.**

**Compound**	**1,2,5,6-tetraphenylpyracylene**
Formula weight, gmol^{-1}	480.57
T (K) / *l* (Å)	100(2)/1.54178
Crystal system	Orthorhombic
Space group / *Z*	Iba2/4
a, Å	15.4325(13)
b, Å	22.966(3)
c, Å	6.9310(5)
α, °	90
β, °	90
γ, °	90
V, Å^3	2456.6(4)
ρ, mg m^{-3}	1.299
Abs. coeff., mm^{-1}	0.559
F(000)	1088
Crystal size, mm^3	0.200 x 0.025 x 0.015
θ range, °	3.45 to 70.32
Reflns collected	12457
Ind. reflns	2291
R(int)	0.0748
Obs. reflns [$I > 2\sigma(I)$]	2021
Completeness to 2θ	99.7
Goodness-of-fit F^2	1.104
Final *R* [$I > 2\sigma(I)$]	R1 = 0.0502<br>wR2 = 0.1332
R (all data)	R1 = 0.0604<br>wR2 = 0.1421

a)

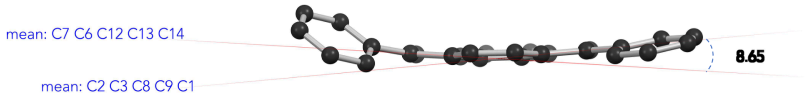

b)

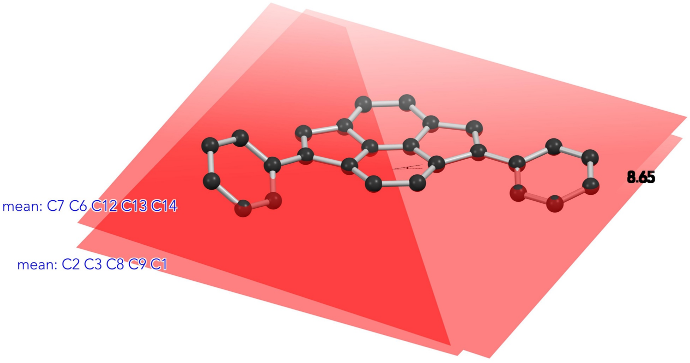

Figure S50. Depictions of the solid-state structure of **1a** showing ~8.6° angle between planes defined by 5-membered rings viewed from (a) side-on and (b) an alternative angle, C: black, H atoms omitted for clarity.

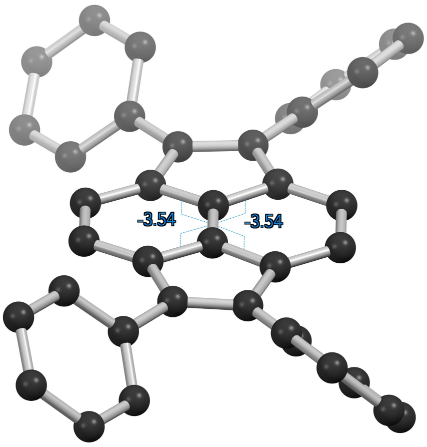

Figure S51. Depiction of the solid-state structure of **3** illustrating the torsion angle of ~3.5° about the central C=C bond. C: black, H atoms omitted for clarity.

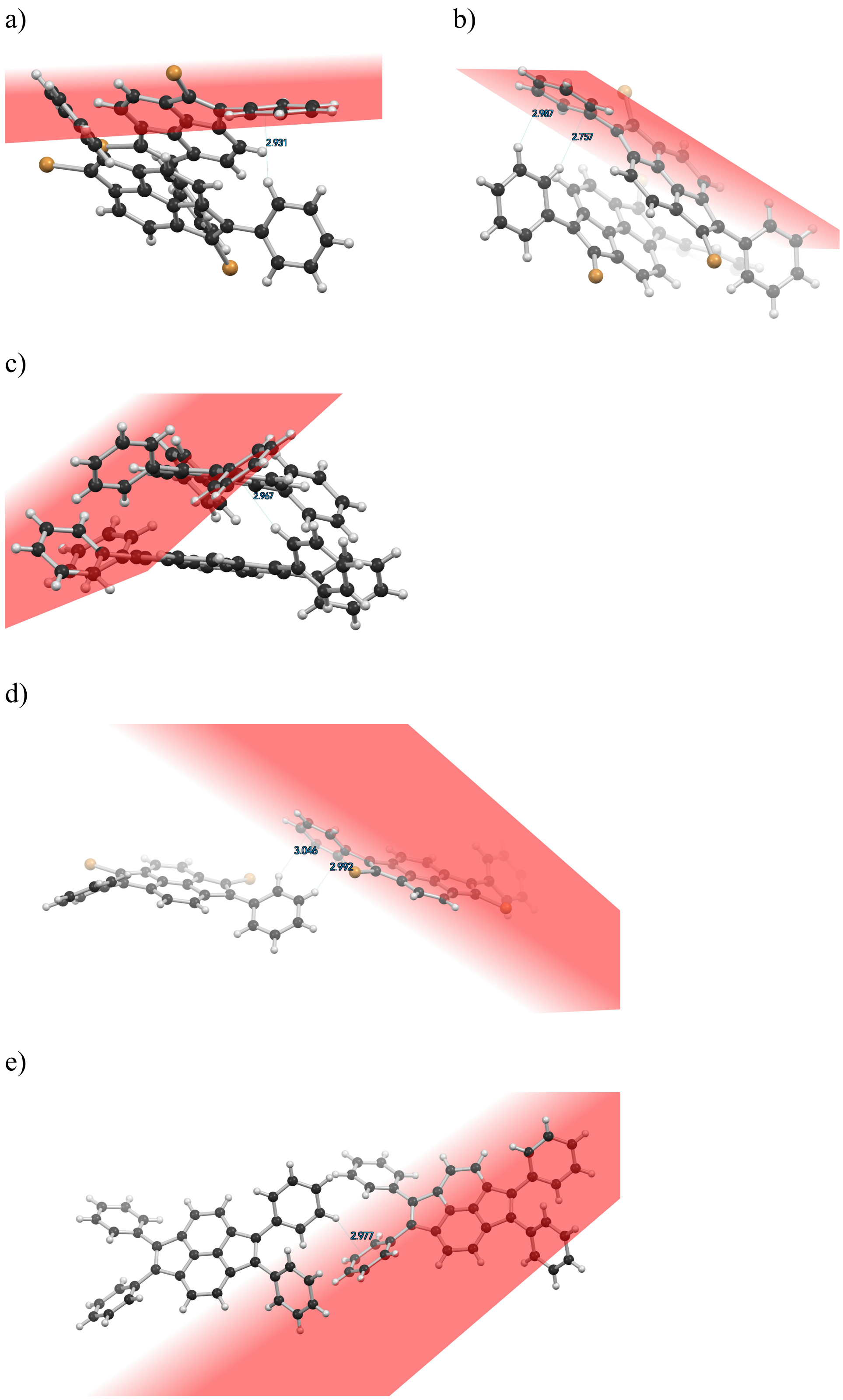

Figure S52. Depiction of solid-state structures showing close CH-π distances between orthogonally oriented phenyl substituents of neighboring pyracylenes within 1D π-stacks of a) **1b**, b) **2b**, and c) **3**, and additionally between neighboring pyracylenes in adjacent stacks of d) **1b** and e) **3**. C: black, Br: orange, distance labels in Å.

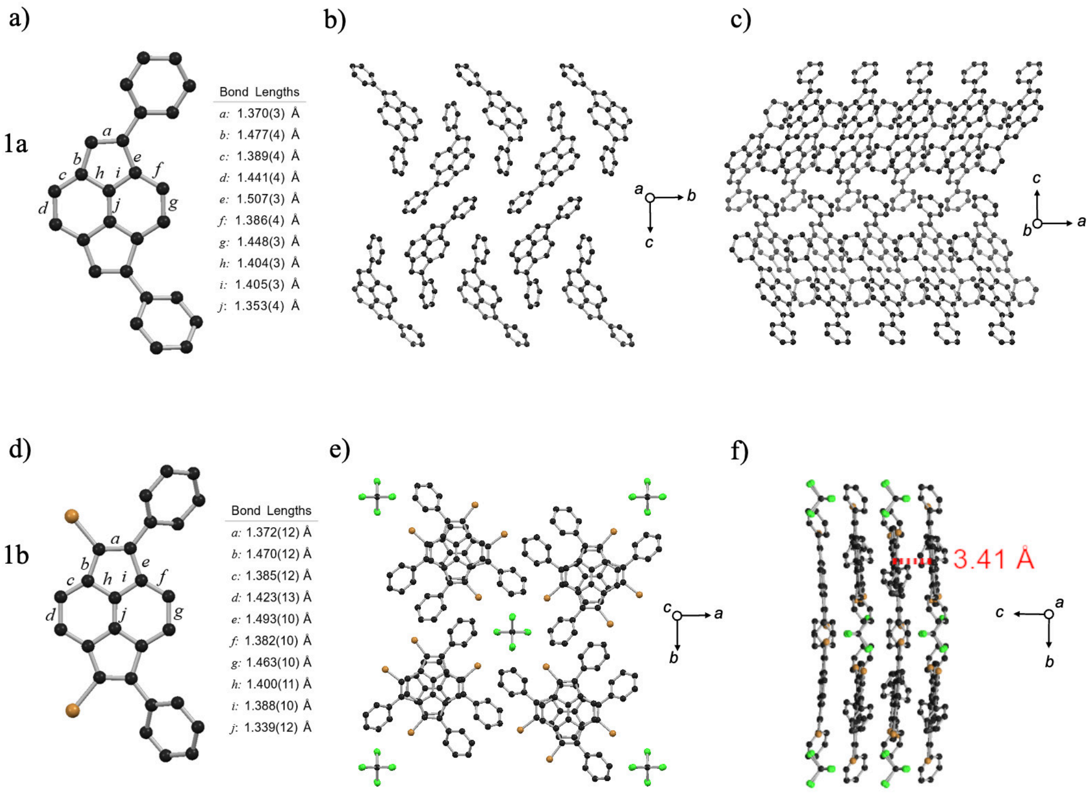

Figure S53. Solid state structures of pyracylenes **1a** and **1b** highlighting molecular structures and bond lengths (a: **1a**, d: **1b**) as well as solid-state packing arrangements (b-c: **1a**, e-f: **1b**). C: black, Br: orange, Cl: green, H atoms omitted for clarity.

8. DFT calculations

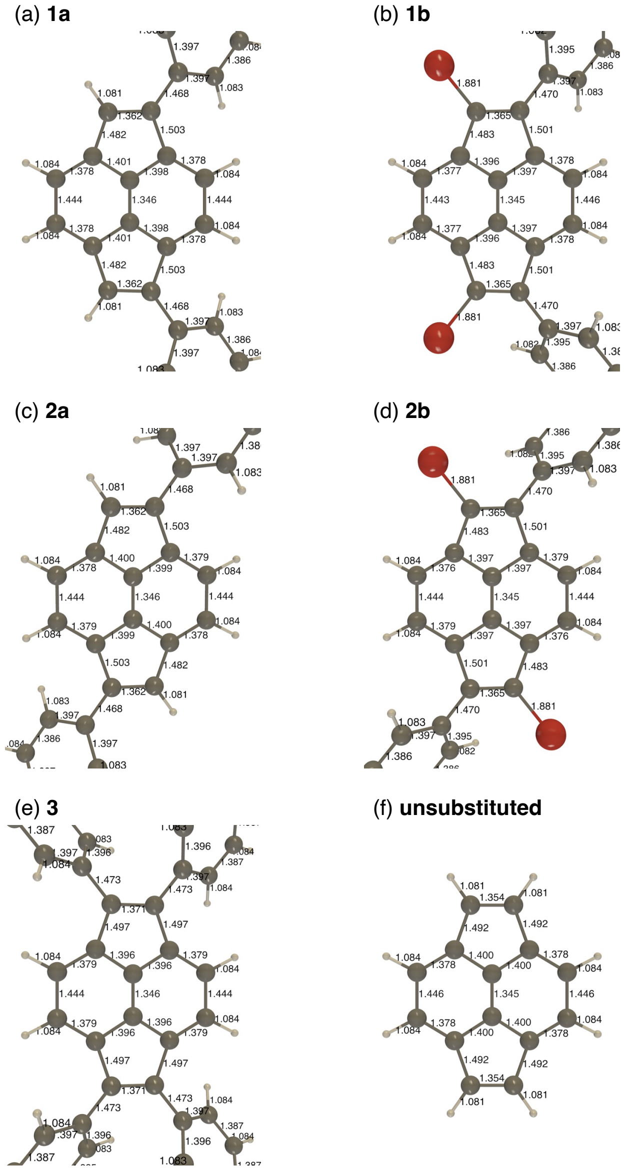

Figure S54. Bond lengths [Å] of the optimized geometries of **1a, 1b, 2a, 2b, 3**, and unsubstituted pyracylene calculated at the RCAM-B3LYP/6-311G(d,p) level.

Simulated UV-Vis spectra

UV-Vis spectra of **1-3** were simulated by the time-dependent (TD-)DFT method using the RB3LYP functional and 6-31+G(d) basis set (**Figure S54** and **Table S5**). Since the structures of **2** and **3** are centrosymmetric, the S_0-S_1 transitions of **2a** and **2b**, described by the HOMO-LUMO single excitation, were forbidden (i.e., the oscillator strength $f = 0$). In the experiment, a weak absorption band at ~500-800 nm was observed even in **2** and **3** due to the thermal structural fluctuations in the solution phase. On the other hand, the structure of **1** is non-centrosymmetric, and thus, the corresponding oscillator strengths of **1a** and **2a** were non-zero. The TD-DFT calculations also reproduced the experimental result that the absorption intensity of **1a** at ~500-800 nm was stronger than that of other molecules.

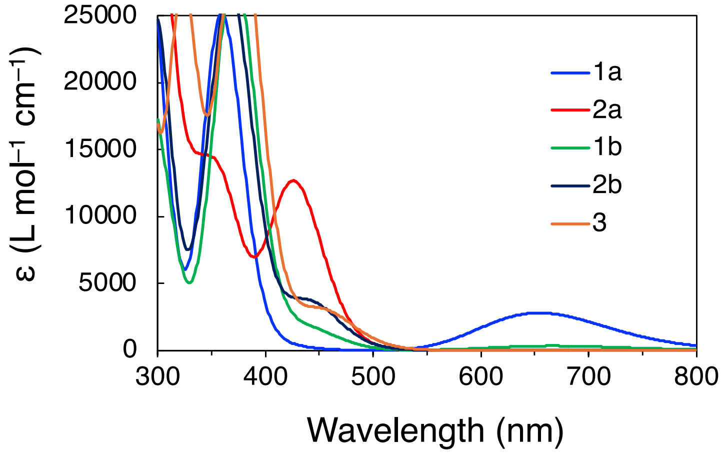

Figure S55 UV-Vis spectra of **1-3** simulated at the TD-B3LYP/6-31+G(d) level. The half-width at the half-height (HWHH) of the Gaussian line-shape function was set to 0.2 eV.

Table S5. Excitation properties calculated at the TD-RB3LYP/6-31+G(d) level.

**1a**						
Excited State	1:	Singlet-A	1.8952 eV	654.21 nm	f=0.0414	<S**2>=0.000
86 -> 87		0.70446				
Excited State	2:	Singlet-A	2.9893 eV	414.76 nm	f=0.0053	<S**2>=0.000
83 -> 87		-0.13921				
85 -> 87		0.68790				
Excited State	3:	Singlet-A	3.4387 eV	360.56 nm	f=0.3459	<S**2>=0.000
84 -> 87		0.68851				
Excited State	4:	Singlet-A	3.5490 eV	349.35 nm	f=0.0323	<S**2>=0.000
83 -> 87		0.60458				

85 -> 87	0.12650					
86 -> 88	0.31764					
Excited State 5:	Singlet-A	3.7841 eV	327.65 nm	f=0.0021	<S**2>=0.000	
82 -> 87	0.70052					
Excited State 6:	Singlet-A	3.7864 eV	327.45 nm	f=0.0021	<S**2>=0.000	
81 -> 87	0.70076					
Excited State 7:	Singlet-A	4.0485 eV	306.25 nm	f=0.0000	<S**2>=0.000	
80 -> 87	0.68564					
Excited State 8:	Singlet-A	4.1298 eV	300.22 nm	f=0.2517	<S**2>=0.000	
79 -> 87	0.13963					
83 -> 87	-0.27018					
84 -> 89	0.13201					
86 -> 88	0.60927					
Excited State 9:	Singlet-A	4.2506 eV	291.69 nm	f=0.0001	<S**2>=0.000	
86 -> 89	0.69116					
Excited State 10:	Singlet-A	4.3938 eV	282.18 nm	f=0.3447	<S**2>=0.000	
86 -> 90	0.68489					
86 -> 91	-0.10876					

1b

Excited State 1:	Singlet-A	1.8606 eV	666.37 nm	f=0.0049	<S**2>=0.000
120 ->121	0.70568				
Excited State 2:	Singlet-A	2.8221 eV	439.33 nm	f=0.0239	<S**2>=0.000
119 ->121	0.69379				
Excited State 3:	Singlet-A	3.3282 eV	372.53 nm	f=0.3959	<S**2>=0.000
118 ->121	0.69115				
Excited State 4:	Singlet-A	3.5331 eV	350.92 nm	f=0.0427	<S**2>=0.000
115 ->121	0.47409				
117 ->121	0.41916				
119 ->121	-0.11427				
120 ->122	-0.24859				
Excited State 5:	Singlet-A	3.5629 eV	347.98 nm	f=0.0015	<S**2>=0.000
116 ->121	0.70351				
Excited State 6:	Singlet-A	3.5634 eV	347.94 nm	f=0.0010	<S**2>=0.000
115 ->121	-0.47304				
117 ->121	0.52035				
Excited State 7:	Singlet-A	3.8539 eV	321.71 nm	f=0.0129	<S**2>=0.000

114 ->121 0.69049

Excited State 8: Singlet-A 4.0354 eV 307.24 nm f=0.0556 <S**2>=0.000
113 ->121 0.53444
120 ->122 -0.43753

Excited State 9: Singlet-A 4.1159 eV 301.23 nm f=0.1253 <S**2>=0.000
113 ->121 0.38833
115 ->121 0.11025
117 ->121 0.11328
118 ->123 -0.11007
120 ->122 0.41774
120 ->123 0.33644

Excited State 10: Singlet-A 4.2504 eV 291.70 nm f=0.0000 <S**2>=0.000
112 ->121 0.70085

2a

Excited State 1: Singlet-AG 1.9151 eV 647.41 nm f=0.0000 <S**2>=0.000
86 -> 87 0.70517

Excited State 2: Singlet-AU 2.9077 eV 426.40 nm f=0.1866 <S**2>=0.000
84 -> 87 -0.20583
85 -> 87 0.67241

Excited State 3: Singlet-AU 3.4377 eV 360.66 nm f=0.1613 <S**2>=0.000
83 -> 87 -0.12584
84 -> 87 0.62488
85 -> 87 0.19735
86 -> 88 -0.17901

Excited State 4: Singlet-AU 3.7261 eV 332.74 nm f=0.1354 <S**2>=0.000
83 -> 87 0.58946
84 -> 87 0.18922
86 -> 88 0.30745

Excited State 5: Singlet-AG 3.7859 eV 327.49 nm f=0.0000 <S**2>=0.000
82 -> 87 0.70128

Excited State 6: Singlet-AU 3.7895 eV 327.18 nm f=0.0109 <S**2>=0.000
81 -> 87 0.69759

Excited State 7: Singlet-AG 3.8893 eV 318.78 nm f=0.0000 <S**2>=0.000
80 -> 87 0.68965
86 -> 89 0.12506

Excited State 8: Singlet-AU 4.1001 eV 302.39 nm f=0.4455 <S**2>=0.000
79 -> 87 -0.11335
83 -> 87 -0.31354

86 -> 88 0.59118

Excited State 9: Singlet-AG 4.2236 eV 293.55 nm f=0.0000 <S**2>=0.000
80 -> 87 -0.11290
86 -> 89 0.66979
86 -> 92 -0.14775

Excited State 10: Singlet-AG 4.4454 eV 278.90 nm f=0.0000 <S**2>=0.000
86 -> 91 0.68472

2b

Excited State 1: Singlet-A 1.8607 eV 666.33 nm f=0.0000 <S**2>=0.000
120 ->121 0.70560

Excited State 2: Singlet-B 2.8159 eV 440.30 nm f=0.0536 <S**2>=0.000
115 ->121 -0.10272
119 ->121 0.69171

Excited State 3: Singlet-B 3.3493 eV 370.18 nm f=0.3540 <S**2>=0.000
115 ->121 -0.13007
118 ->121 0.66854

Excited State 4: Singlet-A 3.5577 eV 348.50 nm f=0.0011 <S**2>=0.000
116 ->121 0.70374

Excited State 5: Singlet-B 3.5612 eV 348.15 nm f=0.0047 <S**2>=0.000
115 ->121 0.19955
117 ->121 0.67193

Excited State 6: Singlet-B 3.5764 eV 346.67 nm f=0.0899 <S**2>=0.000
115 ->121 0.60042
117 ->121 -0.14793
118 ->121 0.17112
118 ->123 0.10113
120 ->122 0.24028

Excited State 7: Singlet-A 3.7277 eV 332.61 nm f=0.0000 <S**2>=0.000
114 ->121 0.69894

Excited State 8: Singlet-B 4.0856 eV 303.47 nm f=0.2409 <S**2>=0.000
113 ->121 -0.37842
115 ->121 -0.15045
120 ->122 0.55725

Excited State 9: Singlet-A 4.1728 eV 297.13 nm f=0.0002 <S**2>=0.000
120 ->123 0.68410

Excited State 10: Singlet-B 4.2080 eV 294.64 nm f=0.0015 <S**2>=0.000
112 ->121 0.69942

3

Excited State	1:	Singlet-B	1.8018 eV	688.12 nm	f=0.0000	<S**2>=0.000
126 ->127		0.70527				
Excited State	2:	Singlet-B	2.7414 eV	452.27 nm	f=0.0445	<S**2>=0.000
123 ->127		0.11314				
125 ->127		0.69535				
Excited State	3:	Singlet-A	3.2980 eV	375.94 nm	f=0.4782	<S**2>=0.000
124 ->127		0.69484				
Excited State	4:	Singlet-B	3.4524 eV	359.13 nm	f=0.0031	<S**2>=0.000
123 ->127		0.57442				
126 ->128		-0.38267				
Excited State	5:	Singlet-B	3.5837 eV	345.97 nm	f=0.0000	<S**2>=0.000
118 ->127		-0.12343				
122 ->127		0.69206				
Excited State	6:	Singlet-B	3.6450 eV	340.15 nm	f=0.0072	<S**2>=0.000
121 ->127		0.67015				
123 ->127		0.10890				
126 ->128		0.17795				
Excited State	7:	Singlet-A	3.7251 eV	332.83 nm	f=0.0000	<S**2>=0.000
120 ->127		0.69136				
126 ->129		0.10167				
Excited State	8:	Singlet-B	3.7881 eV	327.30 nm	f=0.0000	<S**2>=0.000
118 ->127		0.68756				
122 ->127		0.12152				
Excited State	9:	Singlet-A	3.8046 eV	325.88 nm	f=0.0065	<S**2>=0.000
119 ->127		0.70050				
Excited State	10:	Singlet-B	3.8325 eV	323.51 nm	f=0.3851	<S**2>=0.000
115 ->127		0.10758				
121 ->127		-0.20508				
123 ->127		0.32681				
124 ->129		-0.12941				
126 ->128		0.55349				

Intermolecular Interactions

Intermolecular interactions were analyzed by the DFT calculations. An energy decomposition analysis (EDA) can separate a calculated interaction energy into several contributions: Permanent and induced electrostatics (ELEC and POL), Pauli repulsions (PAULI), dispersion (DISP), and charge transfer (CT). Although there have been several different ways of EDA, the second-generation absolutely-localized MO (ALMO)-EDA developed by Head-Gordon and coworkers [5] was employed in this study. The ALMO-EDA and related calculations were performed for a stacked dimer model of **2b**, denoted as **2b**$_2$. The geometry of **2b**$_2$ was taken from the dimeric part of the solid-state structure, and then, only the positions of hydrogen atoms were optimized at the RCAM-B3LYP/6-311G(d,p) level. By replacing the Br atoms of **2b**$_2$ with hydrogens, keeping other geometrical parameters unchanged, we constructed a virtual stacked dimer model of **2a** (denoted as **2a**$_2$). By comparing the results of **2b**$_2$ and **2a**$_2$, the effect of (the number of) substituents on the stabilization of the stacked structure can be examined. Then, the ωB97M-V functional [6] and def2-SVPD basis set were used to evaluate the intermolecular interactions. These ALMO-EDA calculations were performed using Q-Chem 6.1 program package. [7] The ALMO-EDA results (**Table S6**) indicate that the dispersion term (DISP) is dominant in stabilizing the stacked dimer structure, with the magnitude of the DISP being ~14 kJ mol^{-1} larger in **2b**$_2$ than in **2a**$_2$.

Table S6. Results of ALMO-EDA [kJ mol^{-1}] for the stacked dimer models **2b**$_2$ and **2a**$_2$ calculated at the ωB97M-V/def2-SVPD level.

	ELEC+PAULI [a]	DISP [a]	POR [a]	CT [a]	Total [a]
**2b**$_2$	+79.68	–178.77	–11.83	–2.01	–112.92
**2a**$_2$	+84.81	–164.70	–10.54	–1.94	–92.36

[a] ELEC, PAULI, DISP, POR, and CT indicate the electrostatics, Pauli exchange, dispersion, polarization (induced electrostatics), charge transfer contributions, respectively.

Intermolecular interactions between pyracylenes were also analyzed for dimer models consisting of unsubstituted pyracylenes. Based on the optimized geometry of pyracylene monomer, we constructed face-to-face stacking dimer models with different stacking distance d and twist angle θ (**Figure S55a**). Then, the interaction energies E_{int} = E(dimer) – 2E(monomer) were computed at the ωB97M-V/def2-SVPD level. From the calculation results of d- and θ-dependences of E_{int} (**Figure S55b-c**), there is a local

minimum of the potential energy surface of the face-to-face dimer at $\theta = 90^\circ$ and $d \sim 3.4$ Å. The HOMO and HOMO–1 (LUMO and LUMO+1) are degenerated at $\theta = 90^\circ$ (**Figure S55d**), indicating that the orbital interaction between the HOMOs (LUMOs) of monomers vanishes. The parallel stacking configuration ($\theta = 0^\circ$) was relatively unstable, probably due to the exchange repulsion.

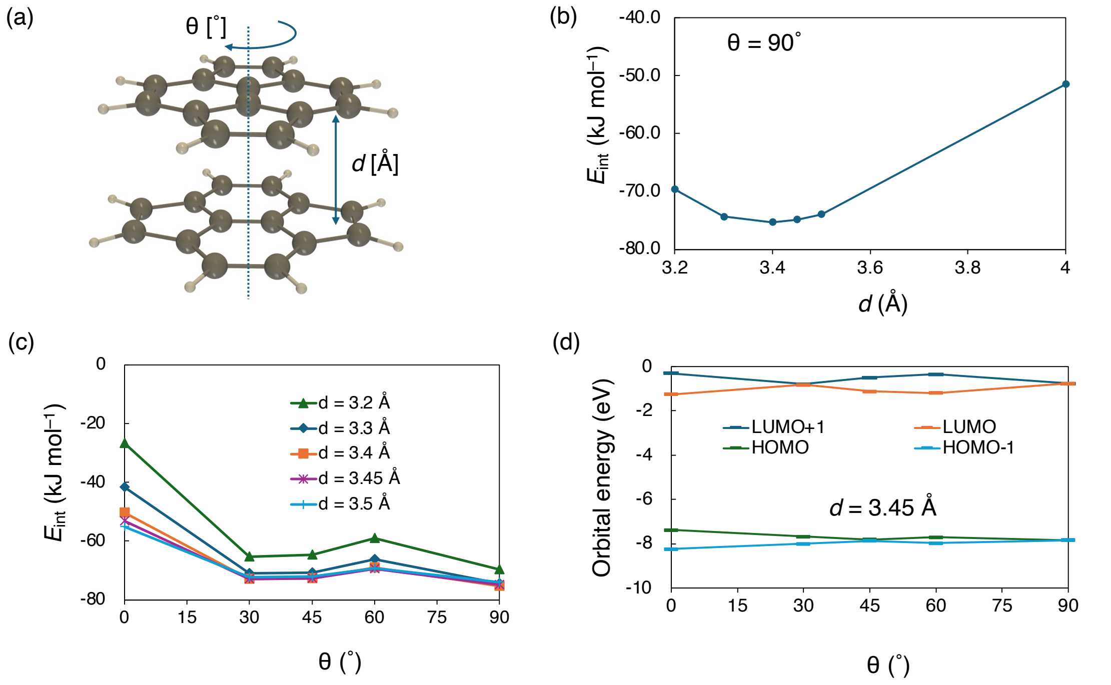

Figure S56. Structure of unsubstituted pyracylene dimer model (a), d-dependence of the interaction energy E_{int} for models with $\theta = 90^\circ$ (b), θ-dependence of E_{int} (c), and θ-dependence of the orbital energies for models with $d = 3.45$ Å (d), calculated at the ωB97M-V/def2-SVPD level.

Although the orbital interaction between the HOMOs (LUMOs) of monomers vanishes at $\theta = 90^\circ$, that between the HOMO of one monomer and the LUMO of the other monomer does not vanish (**Figure S56**). Since the strength of orbital interaction depends on the degree of orbital overlap, this type of orbital interaction is weak in **1b**, **2b**, and **3** in the solid-state structure ($d \sim 3.45$ Å), but may slightly affect the electronic structures.

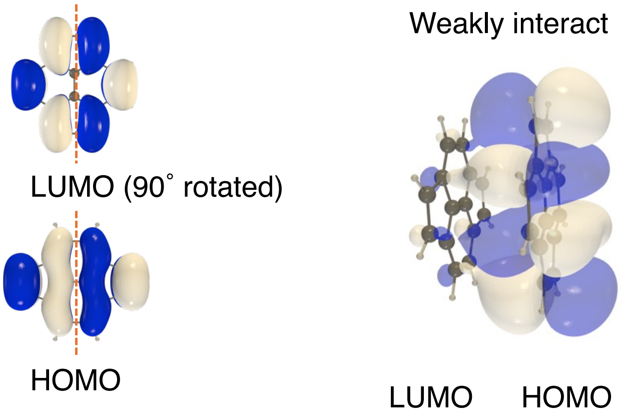

Figure S57. Orbital interaction between the HOMO of one monomer and the LUMO of the other monomer at θ = 90˚.

Magnetic response properties

Magnetic response properties were evaluated at the GIAO-RB3LYP/6-311++G(d,p) level. The NICS-XY scan [8] was performed for the monomer and dimer structures of **1**-**3** using the Aroma-2.0 package. The geometries were taken from the solid-state structure. The $NICS_{\pi zz,\sigma\text{-only}}$ [9] was evaluated 1.7 Å above the molecular plane. For the dimers, the $NICS(1.7)_{\pi zz,\sigma\text{-only}}$ was scanned outside the dimer structure (**Figure S57**). From the calculation results, the $NICS(1.7)_{\pi zz,\sigma\text{-only}}$ values on the five-membered rings in the dimer structure were decreased compared to those in the monomer, i.e., the intermolecular interactions could reduce the paratropicity of five-membered rings to some extent.

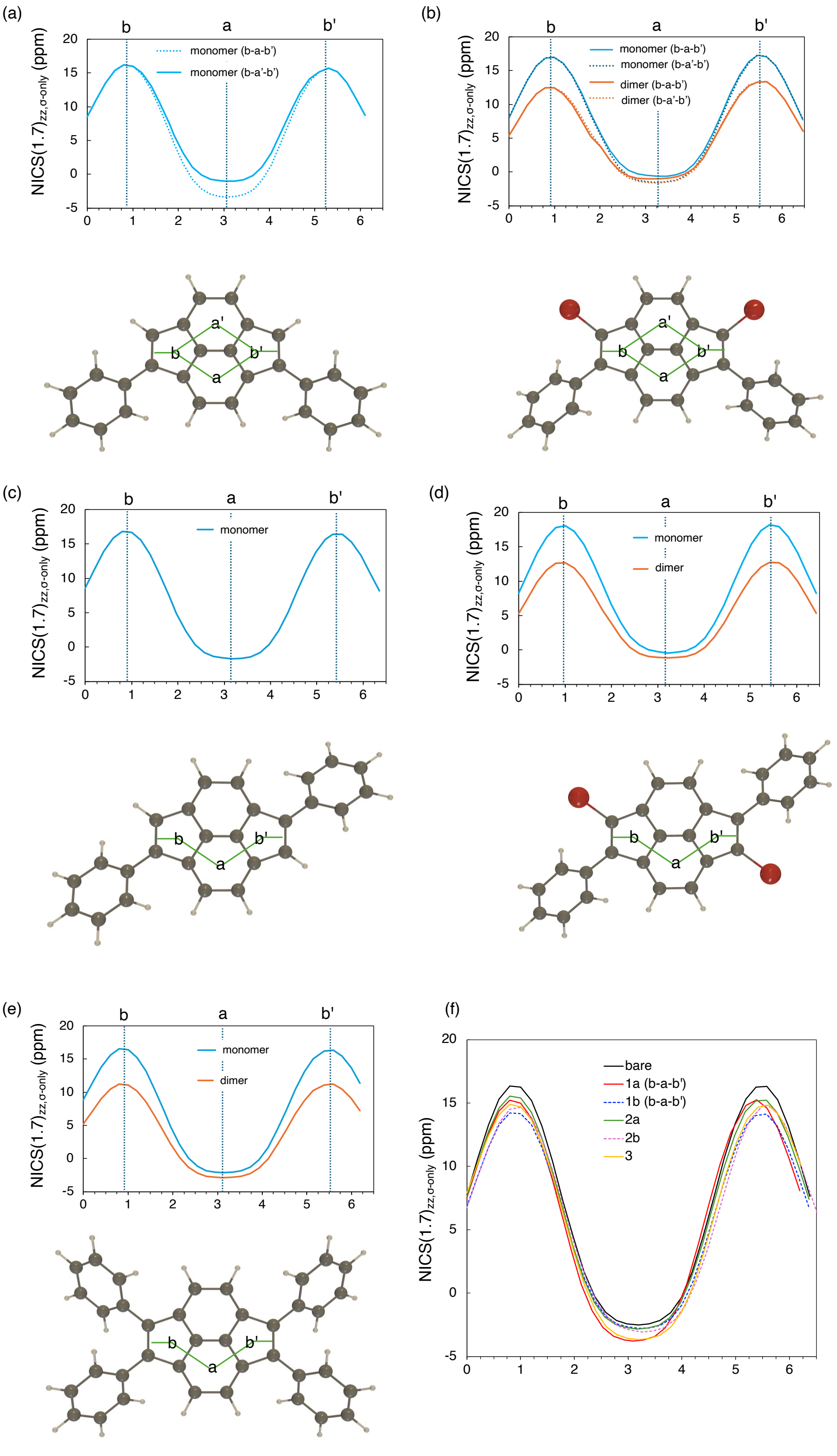

Figure S58. Results of NICS-XY scan of the monomer (and the π-stacking dimer) of **1a** (a), **1b** (b), **2a** (a), **2b** (b), and **3** (e) calculated at the GIAO-RB3LYP/6-311++G(d,p) level based on the solid-state structures, and those of the monomers based on the optimized structures (at the GIAO-RB3LYP/6-311++G(d,p) // RCAM-B3LYP/6-

311G(d,p) level) (f). Note that **1a** and **2a** do not form the stacking structure in the solid-state structure.

On the other hand, **1b**, **2b**, and **3** are shown to form infinite one-dimensional π-stacking structures with uniform stacking distances (*d*) in the solid state. To see how the magnetic properties change with increasing the number of monomers, we performed the NICS calculations for a 10-mer model consisting of unsubstituted pyracylenes with $d = 3.45$ Å and $\theta = 90^{\circ}$ at the same level of approximation. Here, NICS(0)$_{zz}$ values were evaluated at the centers of the five- and six-membered rings of each monomer (**Figure S58**). The NICS(0)$_{zz}$ values in the middle region of the 10-mer were almost converged. The converged value on the five-membered ring (~42.6 ppm) was significantly decreased from the NICS(0)$_{zz}$ value of the monomer (68.5 ppm) and even smaller than that of the dimer (56.0 ppm).

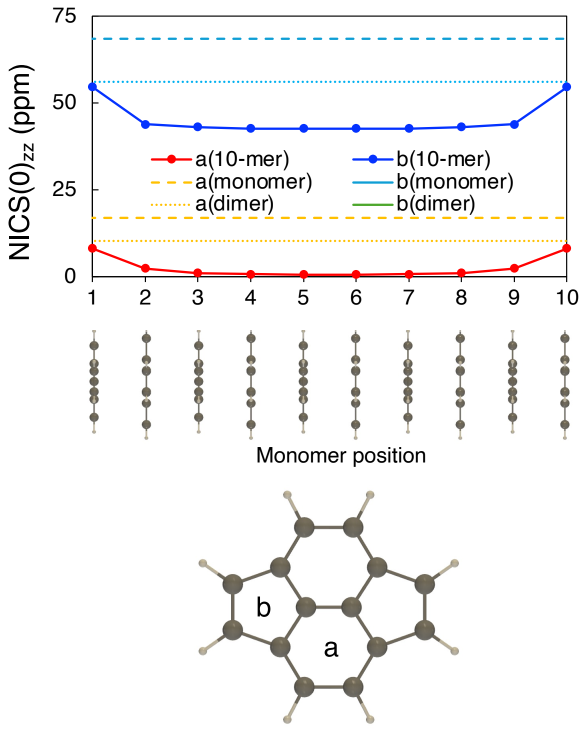

Figure S59. NICS(0)$_{zz}$ [ppm] of the 10-mer model consisting of unsubstituted pyracylenes with $d = 3.45$ Å and $\theta = 90^{\circ}$ (b), calculated at the GIAO-RB3LYP/6-311++G(d,p) level.

To understand why the reduction in paratropicity occurs in the 90°-twisted π-stacking structures, we have examined the energies and topologies of frontier MOs in the

presence of intermolecular interaction. As explained before, in the case of 90°-twisted stacking, HOMOs (LUMOs) of adjacent monomers do not interact with each other, whereas the HOMO of one monomer and the LUMO of the other monomer can interact. The orbital interaction diagram for this situation is illustrated in **Figure S59**. As a result of HOMO-LUMO interaction, the HOMO-LUMO gap is expected to increase in the dimer. Although the orbital interaction may not be strong at *d* ~ 3.45 Å, it can influence the HOMO-LUMO gap.

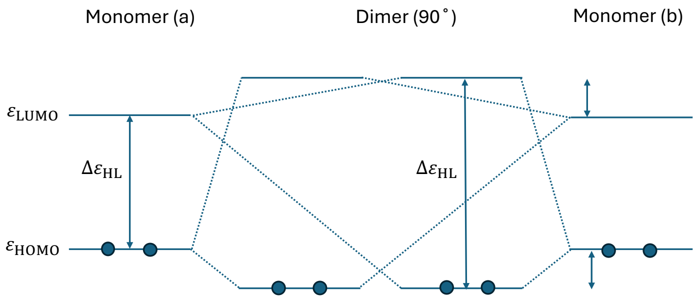

Figure S60. Orbital interaction diagram of the 90°-twisted dimer case.

Based on the perturbative treatment of the magnetic response property of closed-shell molecules, the paratropic contribution of the magnetically-induced current (MIC) density, $\boldsymbol{j}_p(\boldsymbol{r})$, in the presence of a magnetic field along the *z*-axis is approximated by

$$\boldsymbol{j}_p \sim -2\sum_{k=1}^{n/2} \boldsymbol{j}_{p,k}$$

where

$$\boldsymbol{j}_{p,k} = -\frac{i}{2}\sum_{a} \frac{\langle \psi_a | \hat{l}_z | \psi_k \rangle}{\varepsilon_a - \varepsilon_k} (\psi_k \boldsymbol{\nabla} \psi_a - \psi_a \boldsymbol{\nabla} \psi_k)$$

indicates the contribution from the occupied MO ψ_k. $\langle \psi_a | \hat{l}_z | \psi_k \rangle$ is the matrix element describing the magnetic transition from the occupied MO ψ_k to the unoccupied MO ψ_a. The paratropic contribution becomes significant when the orbital energy gap $\varepsilon_a - \varepsilon_k$ is small, as long as $\langle \psi_a | \hat{l}_z | \psi_k \rangle$ remains almost unchanged with respect to the change in *d*.

Table S7 compares the HOMO-LUMO gap ($\Delta\varepsilon_{\mathrm{HL}} = \varepsilon_{\mathrm{LUMO}} - \varepsilon_{\mathrm{HOMO}}$) and NICS_{zz} values on the five-membered ring of unsubstituted pyracylene monomer and 90°-twisted π-stacking dimers at *d* = 3.45 Å and 10.0 Å. $\Delta\varepsilon_{\mathrm{HL}}$ of the dimer at *d* = 3.45 Å is larger than that of the monomer. Correspondingly, the NICS_{zz} values of the dimer at *d* = 3.45 Å are smaller than those of the monomer. We have performed a canonical MO-based

decomposition analysis of the NICS (CMO-NICS) using the NBO program package,[9] which enables us to decompose the NICS values into contributions from the occupied CMOs (values in parentheses in **Table S7**). As shown in **Table S7**, the trend of total $NICS_{zz}$ is described primarily by the trend of the contribution from the degenerate HOMO and HOMO–1, although the contributions from other MOs seem to influence the results to some extent. This result indicates that the unique manner of intermolecular HOMO-LUMO orbital interactions, leading to the increase of $\Delta\varepsilon_{HL}$, is the key for understanding the reduction in paratropicity in the 90°-twisted π-stacking structures. The energy gap between the occupied and unoccupied MOs determining the paratropic contributions is expected to increase as the number of monomers increases and then converge to a certain value, which is reflected in the converged NICS value in the middle region of the 10-mer model.

Table S7. HOMO-LUMO gap ($\Delta\varepsilon_{HL}$) [eV] and $NICS_{zz}$ values on the five-membered ring [ppm] of unsubstituted pyracylene monomer and 90°-twisted π-stacking dimers at *d* = 3.45 Å and 10.0 Å. Orbital energies and magnetic properties were calculated at the RB3LYP/6-311++G(d,p) level.

	monomer	dimer	
		***d* = 3.45 Å**	*d* = 10.0 Å
ε_{HOMO} (eV)	–5.733	**–5.610**	–5.701
ε_{LUMO} (eV)	–2.813	**–2.614**	–2.781
$\Delta\varepsilon_{HL}$ (eV)	2.920	**2.996**	2.920
$NICS(0)_{zz}$ (ppm)	68.47 (70.56)[a]	**56.01 (66.80)**[b]	68.18 (71.09)[b]
$NICS(1.7)_{zz}$ (ppm)	8.22 (30.84)[a]	**2.84 (29.15)**[b]	8.04 (31.19)[b]

a The value in round parentheses indicates the contribution from the HOMO obtained by the CMO-NICS analysis.
b The value in round parentheses indicates the sum of the contributions from the degenerate HOMO and HOMO–1 obtained by the CMO-NICS analysis.

Multi-configurational wavefunction analysis

A multi-configurational wavefunction analysis combined with the diabatization technique was conducted to provide insight into the mechanism of paratropicity reduction. Sugimori *et al.*[10] proposed that the appearance of the double-triplet $^1(T_1T_1)$ configuration, where each monomer is in the Baird-aromatic T_1-like state, in the ground state of closely stacked π-dimers of antiaromatic molecules, such as cyclobutadienes

and Ni(II) norcorroles, is the key to understanding the reduced paratropicy. In addition, mixing the intermolecular CT configurations contributed to the formation of intermolecular covalent-like bonds between the T_1-like monomers. Here, we have performed a similar analysis for the unsubstituted pyracylene dimer models.

Face-to-face stacked dimer models of unsubstituted pyracylenes with 0° and 90° twist angles, along with various distance *d* were constructed. Here, each monomer geometry, optimized at the RCAM-B3LYP/6-311G(d,p) level, was kept fixed. The ground state of the dimer models was calculated by the state-averaged complete-active-space self-consistent field (SA-CASSCF) calculation. The CAS(4e,4o) was used as the active space, and the state-average method was employed for the 20 singlet states. Then, the four diabatic MOs (DMOs) localized to reference molecular HOMOs and LUMOs were obtained by Nakamura-Truhlar's 4-fold way scheme.[11] Based on the DMOs, we represented the ground state wavefunctions of the dimers in the linear combinations of diabatic electron configurations (**Figure S60**). The CASSCF calculations were performed using OpenMolcas[12] program package.

The calculation results of the parallel stacked dimer model ($\theta = 0°$) were similar to those of cyclobudadiene and Ni(II) norcorroles, i.e., the double-triplet (TT) and charge-transfer (CT) configurations became dominant in the ground state wavefunction at small *d*. On the other hand, for $\theta = 90°$, the weight of the TT configuration was small even at small *d*. The weight of the CT-type configuration (**ca** in **Figure S60**), in which one electron is transferred from the HOMO of one monomer to the LUMO of the other monomer, increased to some extent as *d* decreased. This is related to the intermolecular orbital interaction between these MOs (**Figure S56**), as explained before. However, the weight of such CT configurations was negligible at $d > 3.4$ Å, where the intermolecular orbital overlap is small.

These results indicate that the paratropic character on the five-membered rings of pyracylene was reduced in the solid-state structures. However, the mechanism of reduction of the paratropicity was different from those of parallel-stacked cyclobudadiene and Ni(ii) norcorrole dimers.

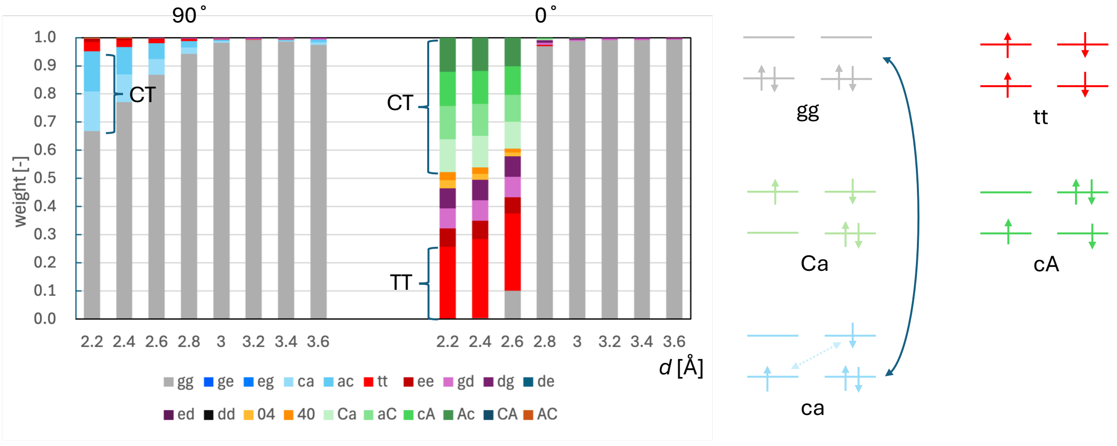

Figure S61. Weight of each diabatic electron configuration in the ground state wavefunction of the unsubstituted pyracylene dimer models with θ = 0˚ and 90˚ as a function of *d*.

Theoretical analysis of electronic coupling

Although there is no orbital interaction between the HOMOs (LUMOs) of adjacent monomers in the 90˚-twisted stacking cases, the TRMC measurements suggest reasonable conducting properties of **1b**, **2b**, and **3** in the solid state. We therefore estimated the electronic coupling between neighboring molecules (the stacked dimer part) in the solid-state structures of **1b**, **2b**, and **3**, which exhibit 90˚-twisted stacking structures. Note that several conducting pathways other than the stacking direction can contribute to the experimental results. We have evaluated the electronic couplings between the diabats based on the multi-state DFT approach combined with the ALMO method [ALMO(MSDFT2)][13] implemented in Q-Chem 6.1 program package.[7] Single point calculations were performed at the spin-unrestricted ωB97X-D/def2-SVP level. We took the geometries of the stacking dimer part from the solid-state structure. Note that the coupling calculation failed for the 90˚-twisted dimer model consisting of unsubstituted pyracylenes, because the effective interstate overlap was zero (i.e., the coupling values were zero). **Table S8** presents the calculation results for electronic couplings in the hole and electron transfers. The couplings of **1b**/**2b** are much larger than those of **3**. This is likely due to the difference in the degree of symmetry reduction, which depends on the substitution position. Two Br and two phenyl groups are introduced in **1b** and **2b**, which disturb the symmetries of HOMO and LUMO of unsubstituted pyracylene to some extent. Consequently, HOMO-HOMO (LUMO-LUMO) couplings between the neighboring molecules in the twisted stacking structures

do not vanish. Even in **3,** where four phenyl groups are introduced at the symmetrically equivalent positions of pyracylene, the structural symmetry of the dimer somewhat reduces from the D_{2h}, because of the relative twist angles of phenyl groups.

From these results, although the intermolecular orbital interactions in the 90°-rotated stacking structures are not strong, various interesting changes in electronic structures and properties are derived.

Table S8. Calculation results of the electronic couplings between neighboring molecules in the solid-state structures of **1b**, **2b**, and **3**, and the orbital energies of dimers calculated at the spin-unrestricted ωB97X-D/def2-SVP level.

	**1b**	**2b**	**3**
hole (meV)	41.9	32.6	11.3
electron (meV)	65.5	42.3	11.1

Cartesian coordinates [Å] of the optimized geometry

1a: RCAM-B3LYP/6-311G(d,p), Etot= -1000.020777 au., no imaginary frequency

C	2.8498978324	0.4341896420	-0.0434218099
C	2.8289219727	1.7937985476	-0.1261096329
H	3.7110105501	2.4179647873	-0.1559915905
C	1.4311814833	2.2803388761	-0.2036557228
C	0.7218850982	3.4613254219	-0.2443685842
H	1.2246875080	4.4218943610	-0.2557561249
C	-0.7218015305	3.4613518331	-0.2443468081
H	-1.2245733486	4.4219376795	-0.2557133308
C	-1.4311261685	2.2803807551	-0.2037810113
C	-0.6730786290	1.1023549293	-0.1796449641
C	0.6730777082	1.1023455682	-0.1794936427
C	1.4321702827	-0.0657717874	-0.0634592200
C	0.7217914180	-1.2393297356	0.0721239274
H	1.2211482174	-2.1914559593	0.2074821772
C	-0.7219251993	-1.2393083992	0.0720912751
H	-1.2213383984	-2.1913994802	0.2074653420
C	-1.4322253330	-0.0657163079	-0.0635710965
C	-2.8499690649	0.4343334575	-0.0435538144
C	-2.8289079389	1.7939162736	-0.1261984563
H	-3.7109609027	2.4181482636	-0.1560196856
C	4.0469017363	-0.4109423336	0.0481591375
C	5.1777896733	0.0158520023	0.7488529690
H	5.1524811805	0.9675238835	1.2657591845
C	6.3135940899	-0.7743609722	0.8122171933
H	7.1788491539	-0.4274869130	1.3646305063
C	6.3412097847	-2.0113321173	0.1823198004
H	7.2283179095	-2.6308104872	0.2356558926
C	5.2230069187	-2.4505767669	-0.5117553389
H	5.2368404312	-3.4127049320	-1.0100117651
C	4.0862058656	-1.6599900255	-0.5764016040
H	3.2262893847	-2.0002110772	-1.1399453712
C	-4.0469002122	-0.4109154979	0.0480544789
C	-5.1776342380	0.0157221639	0.7491570448
H	-5.1521217845	0.9673133344	1.2662083554
C	-6.3133837948	-0.7745118776	0.8126607284
H	-7.1785260912	-0.4278142303	1.3653631098
C	-6.3411544857	-2.0113430660	0.1824318182
H	-7.2282564721	-2.6308130696	0.2358965666
C	-5.2231802824	-2.4503726474	-0.5120782635
H	-5.2370964947	-3.4123506690	-1.0106196680
C	-4.0863696247	-1.6597506628	-0.5768444036
H	-3.2266150139	-1.9998591215	-1.1407076139

1b: RCAM-B3LYP/6-311G(d,p), Etot= -6147.304806 au., no imaginary frequency

Br	-4.2877707192	-2.1629344877	-0.0568294588

Br	4.2876903425	-2.1630585109	0.0566580033
C	-2.8527066256	0.3723456327	-0.0035334778
C	-2.8171659517	-0.9918869535	0.0022255299
C	-1.4221416231	-1.4959400260	-0.0001679664
C	-0.7212321041	-2.6809092019	0.0018975545
H	-1.2324508764	-3.6363130910	0.0060228929
C	0.7213247366	-2.6808891090	-0.0019305243
H	1.2325749737	-3.6362849546	-0.0063518920
C	1.4222023092	-1.4959007293	0.0004747482
C	0.6722846938	-0.3181928089	0.0067049060
C	-0.6722509218	-0.3182106252	-0.0060389427
C	-1.4315441655	0.8541993985	-0.0112666728
C	-0.7229474056	2.0357822476	-0.0006983459
H	-1.2257838150	2.9956392000	0.0078187356
C	0.7229252066	2.0357917358	0.0012416138
H	1.2257205837	2.9956619652	-0.0073894795
C	1.4315574532	0.8542333081	0.0118463476
C	2.8527476633	0.3724095764	0.0037818807
C	2.8171948924	-0.9918370839	-0.0020531768
C	-4.0181139245	1.2685457787	-0.0167811133
C	-5.0933633917	1.0863267231	0.8534424369
H	-5.0758056675	0.2657081141	1.5584216039
C	-6.1717687236	1.9566263753	0.8329502241
H	-6.9964755309	1.8036337188	1.5187030494
C	-6.1956063048	3.0213267106	-0.0566168878
H	-7.0405286870	3.6991887894	-0.0721666252
C	-5.1301008369	3.2148393763	-0.9244584260
H	-5.1422173546	4.0416935424	-1.6244874736
C	-4.0479384262	2.3495727022	-0.9012061307
H	-3.2241894190	2.4971317882	-1.5891876600
C	4.0181186924	1.2686161540	0.0168555390
C	5.0935129848	1.0860608751	-0.8532214419
H	5.0759435711	0.2651921483	-1.5578894103
C	6.1718570433	1.9563375345	-0.8329921984
H	6.9966781528	1.8031123681	-1.5185587005
C	6.1956194284	3.0214526813	0.0561666051
H	7.0405724499	3.6992832370	0.0714346995
C	5.1300763148	3.2152936696	0.9238001349
H	5.1420164683	4.0424212722	1.6235038760
C	4.0478862599	2.3499826557	0.9008017176
H	3.2241942398	2.4978272488	1.5887837284

2a: RCAM-B3LYP/6-311G(d,p), Etot= -1000.020645 au., no imaginary frequency

C	0.8987568922	2.0794689115	4.4045921342
H	1.5928411682	1.3070134059	4.0978079277
C	0.9823465763	2.6112780431	5.6820433145
H	1.7294052906	2.2363566826	6.3715186211
C	0.1184238170	3.6229198222	6.0758748279
H	0.1815168147	4.0359829953	7.0752875862

C	-0.8309892203	4.0990246914	5.1816729344
H	-1.5158903350	4.8824171680	5.4837685514
C	-0.9156871391	3.5677432447	3.9053268243
H	-1.6763345051	3.9236660161	3.2210928909
C	-0.0481428466	2.5530171033	3.4932571231
C	-0.1190848827	2.0049790856	2.1327659309
C	-0.4035531766	2.7056112100	0.9995087617
H	-0.5893812414	3.7699654317	0.9695991789
C	-0.3724451958	1.8061006900	-0.1784456686
C	-0.0545115367	0.5583257836	0.3715527067
C	0.1219347216	0.5691254332	1.7588359575
C	0.4113096926	-0.6456310696	2.3427437179
H	0.5422348487	-0.7421961208	3.4140577365
C	0.5454370641	-1.8419650934	1.5453027028
H	0.7862895473	-2.7645361454	2.0612637813
C	-0.8987568922	-2.0794689115	-4.4045921342
H	-1.5928411682	-1.3070134059	-4.0978079277
C	-0.9823465763	-2.6112780431	-5.6820433145
H	-1.7294052906	-2.2363566826	-6.3715186211
C	-0.1184238170	-3.6229198222	-6.0758748279
H	-0.1815168147	-4.0359829953	-7.0752875862
C	0.8309892203	-4.0990246914	-5.1816729344
H	1.5158903350	-4.8824171680	-5.4837685514
C	0.9156871391	-3.5677432447	-3.9053268243
H	1.6763345051	-3.9236660161	-3.2210928909
C	0.0481428466	-2.5530171033	-3.4932571231
C	0.1190848827	-2.0049790856	-2.1327659309
C	0.4035531766	-2.7056112100	-0.9995087617
H	0.5893812414	-3.7699654317	-0.9695991789
C	0.3724451958	-1.8061006900	0.1784456686
C	0.0545115367	-0.5583257836	-0.3715527067
C	-0.1219347216	-0.5691254332	-1.7588359575
C	-0.4113096926	0.6456310696	-2.3427437179
H	-0.5422348487	0.7421961208	-3.4140577365
C	-0.5454370641	1.8419650934	-1.5453027028
H	-0.7862895473	2.7645361454	-2.0612637813

2b: RCAM-B3LYP/6-311G(d,p), Etot= -6147.304893 au., no imaginary frequency

Br	2.6369741158	3.8515785384	-0.0125452797
C	-2.4530774573	-0.2507687514	-0.0011034046
H	-3.4889505784	-0.5657761965	0.0438937512
C	-2.1724445007	1.1657807445	-0.0041027279
H	-3.0142338998	1.8462587844	0.0445831678
C	-0.8751480372	1.6306249714	-0.0443770883
C	0.1286124072	0.6599835727	-0.0743562970
C	1.4286371782	1.1686396191	-0.0333896005
C	1.2032424269	2.6340864963	0.0032979847
C	-0.1286124072	2.9317657432	-0.0015130829
C	-0.7856836817	4.2466969366	0.0187574635

C	-1.8455474908	4.5013982679	-0.8550836324
H	-2.1512071904	3.7359668384	-1.5581763144
C	-2.4887678831	5.7288181348	-0.8483612720
H	-3.3015706797	5.9134386972	-1.5405122044
C	-2.0916956250	6.7184934504	0.0396911940
H	-2.5961957676	7.6771402486	0.0478251096
C	-1.0471088286	6.4725143672	0.9194459642
H	-0.7369836459	7.2374844759	1.6211565041
C	-0.3981322884	5.2480320265	0.9098826049
H	0.4068510222	5.0589630206	1.6075861356
Br	-2.6369741158	-3.8515785384	-0.0125452797
C	2.4530774573	0.2507687514	-0.0011034046
H	3.4889505784	0.5657761965	0.0438937512
C	2.1724445007	-1.1657807445	-0.0041027279
H	3.0142338998	-1.8462587844	0.0445831678
C	0.8751480372	-1.6306249714	-0.0443770883
C	-0.1286124072	-0.6599835727	-0.0743562970
C	-1.4286371782	-1.1686396191	-0.0333896005
C	-1.2032424269	-2.6340864963	0.0032979847
C	0.1286124072	-2.9317657432	-0.0015130829
C	0.7856836817	-4.2466969366	0.0187574635
C	1.8455474908	-4.5013982679	-0.8550836324
H	2.1512071904	-3.7359668384	-1.5581763144
C	2.4887678831	-5.7288181348	-0.8483612720
H	3.3015706797	-5.9134386972	-1.5405122044
C	2.0916956250	-6.7184934504	0.0396911940
H	2.5961957676	-7.6771402486	0.0478251096
C	1.0471088286	-6.4725143672	0.9194459642
H	0.7369836459	-7.2374844759	1.6211565041
C	0.3981322884	-5.2480320265	0.9098826049
H	-0.4068510222	-5.0589630206	1.6075861356

3: RCAM-B3LYP/6-311G(d,p), Etot= -1461.96970262 au., no imaginary frequency

C	0.72201300	2.33275008	0.32197499
C	1.43109298	1.15955496	0.17334799
C	0.67294198	0.00000000	0.00000000
C	-0.67294198	0.00000000	0.00000000
C	-1.43109298	1.15955496	0.17334799
C	-0.72201300	2.33275008	0.32197499
H	1.22513700	3.28552294	0.43932599
C	1.43109298	-1.15955496	-0.17334799
C	-1.43109298	-1.15955496	-0.17334799
H	-1.22513700	3.28552389	0.43932599
C	-0.72201300	-2.33275008	-0.32197499
C	0.72201300	-2.33275008	-0.32197499
H	-1.22513700	-3.28552389	-0.43932599
H	1.22513700	-3.28552294	-0.43932599
C	-2.84706998	0.67865503	0.09782800
C	2.84706998	0.67865503	0.09782800

C	2.84706998	-0.67865503	-0.09782800
C	-2.84706998	-0.67865503	-0.09782800
C	4.00671816	1.58332205	0.18290100
C	4.06042480	2.56181502	1.17827404
C	5.05595207	1.51058698	-0.73553801
C	5.13784122	3.43095589	1.26242697
H	3.25726199	2.62705994	1.90266705
C	6.12962198	2.38204002	-0.65319300
H	5.02067423	0.76794100	-1.52244902
C	6.17648888	3.34412694	0.34687901
H	5.16586399	4.17778301	2.04709101
H	6.93278599	2.31256390	-1.37721097
H	7.01692820	4.02490997	0.40951201
C	-4.00671816	-1.58332205	-0.18290100
C	-5.05595207	-1.51058698	0.73553801
C	-4.06042480	-2.56181598	-1.17827404
C	-6.12962198	-2.38204002	0.65319300
H	-5.02067423	-0.76794100	1.52244902
C	-5.13783979	-3.43095589	-1.26242697
H	-3.25726199	-2.62706089	-1.90266705
C	-6.17648888	-3.34412694	-0.34687901
H	-6.93278599	-2.31256390	1.37721097
H	-5.16586399	-4.17778301	-2.04709101
H	-7.01692820	-4.02490997	-0.40951201
C	-4.00671816	1.58332205	0.18290100
C	-5.05595207	1.51058698	-0.73553801
C	-4.06042480	2.56181598	1.17827404
C	-6.12962198	2.38204002	-0.65319300
H	-5.02067518	0.76794100	-1.52244902
C	-5.13783979	3.43095589	1.26242697
H	-3.25726199	2.62706089	1.90266597
C	-6.17648888	3.34412694	0.34687901
H	-6.93278599	2.31256294	-1.37721002
H	-5.16586399	4.17778397	2.04709101
H	-7.01692820	4.02490997	0.40951201
C	4.00671816	-1.58332205	-0.18290100
C	4.06042480	-2.56181502	-1.17827404
C	5.05595207	-1.51058698	0.73553801
C	5.13784122	-3.43095589	-1.26242697
H	3.25726199	-2.62705994	-1.90266705
C	6.12962198	-2.38204002	0.65319300
H	5.02067423	-0.76794100	1.52244902
C	6.17648888	-3.34412694	-0.34687901
H	5.16586399	-4.17778301	-2.04709101
H	6.93278599	-2.31256390	1.37721097
H	7.01692820	-4.02490997	-0.40951201

Unsubstituted pyracylene: RCAM-B3LYP/6-311G(d,p), Etot= -538.065429 au., no imaginary frequency

C	0.0000000000	2.3575359990	0.7229310011
C	0.0000000000	1.1760739980	1.4314099979
C	0.0000000000	0.0000000000	0.6726160011
C	0.0000000000	0.0000000000	-0.6726160011
C	0.0000000000	1.1760739980	-1.4314099979
C	0.0000000000	2.3575359990	-0.7229310011
H	0.0000000000	3.3189469966	1.2241810008
C	0.0000000000	-1.1760739980	1.4314099979
C	0.0000000000	-1.1760739980	-1.4314099979
H	0.0000000000	3.3189469966	-1.2241810008
C	0.0000000000	-2.3575359990	-0.7229310011
C	0.0000000000	-2.3575359990	0.7229310011
H	0.0000000000	-3.3189469966	-1.2241810008
H	0.0000000000	-3.3189469966	1.2241810008
C	0.0000000000	0.6770479978	-2.8369519988
C	0.0000000000	0.6770479978	2.8369519988
C	0.0000000000	-0.6770479978	2.8369519988
C	0.0000000000	-0.6770479978	-2.8369519988
H	0.0000000000	1.2980229973	-3.7218309963
H	0.0000000000	1.2980229973	3.7218309963
H	0.0000000000	-1.2980229973	3.7218309963
H	0.0000000000	-1.2980229973	-3.7218309963

9. References

(1) Farrell, J. M.; Mützel, C.; Bialas, D.; Rudolf, M.; Menekse, K.; Krause, A.-M.; Stolte, M.; Würthner, F. Tunable Low-LUMO Boron-Doped Polycyclic Aromatic Hydrocarbons by General One-Pot C–H Borylations. *J. Am. Chem. Soc.* **2019**, *141*, 9096–9104.

(2) Sheldrick, G. A short history of *SHELX Acta Cryst. A*, **2008**, *64*, 112-122.

(3) Frisch, M. J.; Trucks, G. W.; Schlegel, H. B.; Scuseria, G. E.; Robb, M. A.; Cheeseman, J. R.; Scalmani, G.; Barone, V.; Petersson, G. A.; Nakatsuji, H.; Li, X.; Caricato, M.; Marenich, A. V.; Bloino, J.; Janesko, B. G.; Gomperts, R.; Mennucci, B.; Hratchian, H. P.; Ortiz, J. V.; Izmaylov, A. F.; Sonnenberg, J. L.; Williams-Young, D.; Ding, F.; Lipparini, F.; Egidi, F.; Goings, J.; Peng, B.; Petrone, A.; Henderson, T.; Ranasinghe, D.; Zakrzewski, V. G.; Gao, J.; Rega, N.; Zheng, G.; Liang, W.; Hada, M.; Ehara, M.; Toyota, K.; Fukuda, R.; Hasegawa, J.; Ishida, M.; Nakajima, T.; Honda, Y.; Kitao, O.; Nakai, H.; Vreven, T.; Throssell, K.; Montgomery, J. A., Jr.; Peralta, J. E.; Ogliaro, F.; Bearpark, M. J.; Heyd, J. J.; Brothers, E. N.; Kudin, K. N.; Staroverov, V. N.; Keith, T. A.; Kobayashi, R.; Normand, J.; Raghavachari, K.; Rendell, A. P.; Burant, J. C.; Iyengar, S. S.; Tomasi, J.; Cossi, M.; Millam, J. M.; Klene, M.; Adamo, C.; Cammi, R.; Ochterski, J. W.; Martin, R. L.; Morokuma, K.; Farkas, O.; Foresman, J. B.; Fox, D. J. Gaussian 16, Revision C.01, Gaussian, Inc., Wallingford CT, **2016**.

(4) Yanai, T.; Tew, D.; N. Handy, A New Hybrid Exchange–Correlation Functional Using the Coulomb-Attenuating Method (CAM-B3LYP). *Chem. Phys. Lett.* **2004**, *393*, 51–57.

(5) Horn, P.R.; Mao, Y.; Head-Gordon, M. Probing Non-Covalent Interactions with a Second Generation Energy Decomposition Analysis Using Absolutely Localized Molecular Orbitals. *Phys. Chem. Chem. Phys.* **2016**, *18*, 23067–23079.

(6) Mardirossian, N.; Head-Gordon, M. ωB97M-V: A Combinatorially Optimized, Range-Separated Hybrid, Meta-GGA Density Functional with VV10 Nonlocal Correlation, *J. Chem. Phys.* **2016**, *144*, 214110.

(7) Epifanovsky, E.; Gilbert, A. T. B.; Feng, X.; Lee, J.; Mao, Y.; Mardirossian, N.; Pokhilko, P.; White, A. F.; Coons, M. P.; Dempwolff, A. L.; Gan, Z.; Hait, D.; Horn, P. R.; Jacobson, L. D.; Kaliman, I.; Kussmann, J.; Lange, A. W.; Lao, K. U.; Levine, D. S.; Liu, J.; McKenzie, S. C.; Morrison, A. F.; Nanda, K. D.; Plasser, F.; Rehn, D. R.; Vidal, M. L.; You, Z.-Q.; Zhu, Y.; Alam, B.; Albrecht, B. J.; Aldossary, A.; Alguire, E.; Andersen, J. H.; Athavale, V.; Barton, D.; Begam, K.; Behn, A.; Bellonzi, N.; Bernard, Y. A.; Berquist, E. J.; Burton, H. G. A.; Carreras, A.; Carter-Fenk, K.; Chakraborty, R.; Chien, A. D.; Closser, K. D.; Cofer-Shabica, V.;

Dasgupta, S.; de Wergifosse, M.; Deng, J.; Diedenhofen, M.; Do, H.; Ehlert, S.; Fang, P.-T.; Fatehi, S.; Feng, Q.; Friedhoff, T.; Gayvert, J.; Ge, Q.; Gidofalvi, G.; Goldey, M.; Gomes, J.; González-Espinoza, C. E.; Gulania, S.; Gunina, A. O.; Hanson-Heine, M. W. D.; Harbach, P. H. P.; Hauser, A.; Herbst, M. F.; Hernández Vera, M.; Hodecker, M.; Holden, Z. C.; Houck, S.; Huang, X.; Hui, K.; Huynh, B. C.; Ivanov, M.; Jász, Á.; Ji, H.; Jiang, H.; Kaduk, B.; Kähler, S.; Khistyaev, K.; Kim, J.; Kis, G.; Klunzinger, P.; Koczor-Benda, Z.; Koh, J. H.; Kosenkov, D.; Koulias, L.; Kowalczyk, T.; Krauter, C. M.; Kue, K.; Kunitsa, A.; Kus, T.; Ladjánszki, I.; Landau, A.; Lawler, K. V.; Lefrancois, D.; Lehtola, S.; Li, R. R.; Li, Y.-P.; Liang, J.; Liebenthal, M.; Lin, H.-H.; Lin, Y.-S.; Liu, F.; Liu, K.-Y.; Loipersberger, M.; Luenser, A.; Manjanath, A.; Manohar, P.; Mansoor, E.; Manzer, S. F.; Mao, S.-P.; Marenich, A. V.; Markovich, T.; Mason, S.; Maurer, S. A.; McLaughlin, P. F.; Menger, M. F. S. J.; Mewes, J.-M.; Mewes, S. A.; Morgante, P.; Mullinax, J. W.; Oosterbaan, K. J.; Paran, G.; Paul, A. C.; Paul, S. K.; Pavošević, F.; Pei, Z.; Prager, S.; Proynov, E. I.; Rák, Á.; Ramos-Cordoba, E.; Rana, B.; Rask, A. E.; Rettig, A.; Richard, R. M.; Rob, F.; Rossomme, E.; Scheele, T.; Scheurer, M.; Schneider, M.; Sergueev, N.; Sharada, S. M.; Skomorowski, W.; Small, D. W.; Stein, C. J.; Su, Y.-C.; Sundstrom, E. J.; Tao, Z.; Thirman, J.; Tornai, G. J.; Tsuchimochi, T.; Tubman, N. M.; Veccham, S. P.; Vydrov, O.; Wenzel, J.; Witte, J.; Yamada, A.; Yao, K.; Yeganeh, S.; Yost, S. R.; Zech, A.; Zhang, I. Y.; Zhang, X.; Zhang, Y.; Zuev, D.; Aspuru-Guzik, A.; Bell, A. T.; Besley, N. A.; Bravaya, K. B.; Brooks, B. R.; Casanova, D.; Chai, J.-D.; Coriani, S.; Cramer, C. J.; Cserey, G.; DePrince, A. E.; DiStasio, R. A.; Dreuw, A.; Dunietz, B. D.; Furlani, T. R.; Goddard, W. A.; Hammes-Schiffer, S.; Head-Gordon, T.; Hehre, W. J.; Hsu, C.-P.; Jagau, T.-C.; Jung, Y.; Klamt, A.; Kong, J.; Lambrecht, D. S.; Liang, W.; Mayhall, N. J.; McCurdy, C. W.; Neaton, J. B.; Ochsenfeld, C.; Parkhill, J. A.; Peverati, R.; Rassolov, V. A.; Shao, Y.; Slipchenko, L. V.; Stauch, T.; Steele, R. P.; Subotnik, J. E.; Thom, A. J. W.; Tkatchenko, A.; Truhlar, D. G.; Van Voorhis, T.; Wesolowski, T. A.; Whaley, K. B.; Woodcock, H. L.; Zimmerman, P. M.; Faraji, S.; Gill, P. M. W.; Head-Gordon, M.; Herbert, J. M.; Krylov, A. I. Software for the Frontiers of Quantum Chemistry: An Overview of Developments in the Q-Chem 5 Package. *J. Chem. Phys.* **2021**, *155*.

(8) Gershoni-Poranne, R.; Stanger, A. The NICS-XY-Scan: Identification of Local and Global Ring Currents in Multi-Ring Systems. *Chem.–Eur. J.* **2014**, *20*, 5673–5688.

(9) Stanger, A. Obtaining Relative Induced Ring Currents Quantitatively from NICS. *J. Org. Chem.* **2010**, *75*, 2281–2288.

(10) Sugimori, R.; Okada, K.; Kishi, R.; Kitagawa, Y. Stacked-Ring Aromaticity From the Viewpoint of the Effective Number of π-Electrons. *Chem. Sci.* **2025**, *16*, 1707–

1715.

(11) Nakamura, H.; Truhlar, D.G. Direct Diabatization of Electronic States by the Fourfold Way. II. Dynamical Correlation and Rearrangement Processes. *J. Chem. Phys.* **2002**, *117*, 5576–5593.

(12) Li Manni, G.; Fdez. Galván, I.; Alavi, A.; Aleotti, F.; Aquilante, F.; Autschbach, J.; Avagliano, D.; Baiardi, A.; Bao, J. J.; Battaglia, S.; Birnoschi, L.; Blanco-González, A.; Bokarev, S. I.; Broer, R.; Cacciari, R.; Calio, P. B.; Carlson, R. K.; Carvalho Couto, R.; Cerdán, L.; Chibotaru, L. F.; Chilton, N. F.; Church, J. R.; Conti, I.; Coriani, S.; Cuéllar-Zuquin, J.; Daoud, R. E.; Dattani, N.; Decleva, P.; de Graaf, C.; Delcey, M. G.; De Vico, L.; Dobrautz, W.; Dong, S. S.; Feng, R.; Ferré, N.; Filatov(Gulak), M.; Gagliardi, L.; Garavelli, M.; González, L.; Guan, Y.; Guo, M.; Hennefarth, M. R.; Hermes, M. R.; Hoyer, C. E.; Huix-Rotllant, M.; Jaiswal, V. K.; Kaiser, A.; Kaliakin, D. S.; Khamesian, M.; King, D. S.; Kochetov, V.; Krośnicki, M.; Kumaar, A. A.; Larsson, E. D.; Lehtola, S.; Lepetit, M.-B.; Lischka, H.; López Ríos, P.; Lundberg, M.; Ma, D.; Mai, S.; Marquetand, P.; Merritt, I. C. D.; Montorsi, F.; Mörchen, M.; Nenov, A.; Nguyen, V. H. A.; Nishimoto, Y.; Oakley, M. S.; Olivucci, M.; Oppel, M.; Padula, D.; Pandharkar, R.; Phung, Q. M.; Plasser, F.; Raggi, G.; Rebolini, E.; Reiher, M.; Rivalta, I.; Roca-Sanjuán, D.; Romig, T.; Safari, A. A.; Sánchez-Mansilla, A.; Sand, A. M.; Schapiro, I.; Scott, T. R.; Segarra-Martí, J.; Segatta, F.; Sergentu, D.-C.; Sharma, P.; Shepard, R.; Shu, Y.; Staab, J. K.; Straatsma, T. P.; Sørensen, L. K.; Tenorio, B. N. C.; Truhlar, D. G.; Ungur, L.; Vacher, M.; Veryazov, V.; Voß, T. A.; Weser, O.; Wu, D.; Yang, X.; Yarkony, D.; Zhou, C.; Zobel, J. P.; Lindh, R. The OpenMolcas Web: A Community-Driven Approach to Advancing Computational Chemistry. *J. Chem. Theory Comput.* **2023**, *19*, 6933–6991.

(13) Mao, Y.; Montoya-Castillo, A.; Markland, T.E., Accurate and efficient DFT-based diabatization for hole and electron transfer using absolutely localized molecular orbitals. *J. Chem. Phys.* **2019**, *151*, 164114.
